# Stakeholder experiences, attitudes and perspectives on inclusive education for children with developmental disabilities in sub-Saharan Africa: A systematic review of qualitative studies

**DOI:** 10.1177/13623613221096208

**Published:** 2022-05-30

**Authors:** Elisa Genovesi, Cecilia Jakobsson, Lena Nugent, Charlotte Hanlon, Rosa A Hoekstra

**Affiliations:** 1 King’s College London, UK; 2 Sussex Partnership NHS Foundation Trust, UK; 3 University Medical Center Hamburg-Eppendorf, Germany; 4 Addis Ababa University, Ethiopia

**Keywords:** Africa South of the Sahara, autism, developmental disabilities, education services, mainstream schools

## Abstract

Inclusive education is a key strategy in addressing the needs of children with autism and other developmental disabilities in sub-Saharan Africa, who rarely access specialist care or quality education. We aimed to systematically review qualitative research on stakeholder experiences, attitudes and perspectives on inclusive education for pupils with developmental disabilities in mainstream schools in sub-Saharan Africa. We searched five databases and selected relevant studies through a two-stage screening process. We synthesised the papers identified through template analysis of the Results and Discussion sections, guided by the *Consolidated Framework for Implementation Research*. Thirty-two publications met the inclusion criteria. The studies were conducted in seven countries and explored the experiences of pupils with developmental disabilities, parents, peers without developmental disabilities and teachers. Multiple barriers (e.g. unclear policies, insufficient training and support for teachers) and opportunities (e.g. teachers’ commitment to inclusion, collaboration between teachers, the work of non-governmental organisations (NGOs)) for implementing inclusive education for pupils with developmental disabilities in sub-Saharan Africa were identified, occurring across national and community contexts and school, classroom and individual teacher levels. To effectively implement inclusive education for pupils with developmental disabilities, teachers need access to appropriate training, resources and support. Governments can capitalise on motivated teachers and the relevant work of NGOs.

**Lay abstract:**

In sub-Saharan Africa, there are few services for children with developmental disabilities such as autism and intellectual disability. One way to support these children is to include them in mainstream schools. However, currently, African children with developmental disabilities are often excluded from mainstream education opportunities. People involved (e.g. teachers, families and children) can offer information on factors that could ease or interfere with inclusion. This article discusses the findings of published studies that explored the views of relevant groups on including children with developmental disabilities in mainstream schools in sub-Saharan Africa. We systematically searched the literature and identified 32 relevant articles from seven countries in sub-Saharan Africa. We found that unclear policies and insufficient training, resources and support for teachers often blocked the implementation of inclusive education. Factors in favour of inclusive education were the commitment of many teachers to include pupils with developmental disabilities and the work of non-governmental organisations (NGOs), which provided resources and training. This review suggests that motivated teachers should be provided with appropriate training, resources and support for inclusive education, directly and by promoting the work of NGOs.

## Introduction

Around 95% of children with autism and other developmental disabilities (DD) live in low- and middle-income countries (Global Research on Developmental Disabilities Collaborators, 2018). In sub-Saharan Africa, the number of children with DD including autism has increased by 71% over the past 25 years (Global Research on Developmental Disabilities Collaborators, 2018). However, research on autism is limited in this region (Abubakar et al., 2016; Bakare et al., 2022; Divan et al., 2021; Franz et al., 2017). Similar gaps exist in service development and knowledge on other DD, which often co-occur with diagnosed autism, especially in low-resource contexts where only more severe cases come to clinical attention.

Autism and other DD are a major global source of disability and healthcare needs (Colombi & Ghaziuddin, 2017; Global Research on Developmental Disabilities Collaborators, 2018). In low-income settings, families of children with DD tend to receive little or no formal support and are financially strained by healthcare costs and the inability to work due to caring responsibilities (Colombi & Ghaziuddin, 2017; Global Research on Developmental Disabilities Collaborators, 2018). Similar challenges have been reported across sub-Saharan Africa (Ambikile & Outwater, 2012; Dambi et al., 2015; Gona et al., 2016; Schlebusch & Dada, 2018; Tilahun et al., 2016) often with detrimental effects on the quality of life of children with DD and their families (Ambikile & Outwater, 2012; Schlebusch et al., 2017), aggravated by stigma and lack of support by community members, healthcare workers and education professionals (Ajuwon & Brown, 2012; Bakare et al., 2009; Gona et al., 2016; Tekola et al., 2016; Tilahun et al., 2016). The inadequacy or absence of specialist child mental healthcare has been noted in several African countries, including Uganda, Ethiopia, Kenya, Ghana, Zambia and to some extent South Africa (Akol et al., 2015; Getanda et al., 2017; Kleintjes et al., 2010; Tekola et al., 2020; Tilahun et al., 2016; Van Schalkwyk et al., 2016). Limited access to social services and education represents a further challenge for children with DD and their families (Ambikile & Outwater, 2012; Ireri et al., 2019; Tekola et al., 2016, 2020; Tilahun et al., 2016).

The childhood manifestation of DD calls for services and inclusion in relevant platforms of care that can support development throughout childhood and adolescence (Kieling et al., 2011). Schools, when offering sufficient assistance, can be ideal platforms for such services, also ensuring that caring responsibilities are not placed exclusively on families (Uba & Nwoga, 2016). Nonetheless, pupils with DD in sub-Saharan Africa remain mostly unschooled (McKenzie et al., 2013), as they are often excluded from mainstream education, and face availability, accessibility and affordability barriers to enrolment in special schools: these are usually few and expensive institutions, located in the capital and with low capacity (Tekola et al., 2016; Van der Linde et al., 2019).

While specific data on schooling for children with DD are lacking, World Bank data from 13 sub-Saharan African countries indicated that 12-year-old children with any disabilities are substantially less likely to have ever enrolled in school than their peers (World Bank, 2018). Notably, pupils with DD are likely to experience higher exclusion rates than children with sensory or physical impairments. For example, in a recent Ugandan population-based study (Andrews et al., 2020) on cerebral palsy, a third of children with the condition aged 6–17 years attended school, but this proportion decreased to only 8% for those with a co-morbid diagnosis of intellectual disabilities. While not specifically categorised within the diagnostic labels of autism or other DD, global data from the United Nations Children’s Fund (UNICEF, 2021) show that, among children with disabilities, the most likely to be out of school and to have never attended school are those with difficulties in communication and/or self-care. In addition, children who struggle to make friends are more likely to be out of school than those with physical or vision disabilities (UNICEF, 2021).

### Striving for inclusive education

Inclusive education (IE), the practice of addressing the diverse needs of all learners in mainstream classrooms, is recognised by international standards as key to enabling children with disabilities to realise their right to full participation in the community, as per the United Nations’ *Convention of the Rights of Persons with Disabilities* (UN General Assembly, 2007). Ministries and Departments of Education across sub-Saharan Africa strive to promote IE, through education policies (Nigeria Federal Ministry of Education, 2015; South Africa Federal Ministry of Education, 2015) and action plans (Federal Democratic Republic of Ethiopia Ministry of Education, 2020; United Republic of Tanzania Ministry of Education, 2017), although only 42% of Sub-Saharan African countries have IE policies (UNESCO Global Education Monitoring Report Team, 2020).

Beyond policies, IE has yet to be fully implemented in sub-Saharan Africa (Chataika et al., 2012; UNESCO Global Education Monitoring Report Team, 2020). While some strategies relevant to pupils with DD are proposed in action plans (e.g. Federal Democratic Republic of Ethiopia Ministry of Education, 2015), so far, implementation efforts are mainly focussed on providing equipment and infrastructure accommodations for children with physical and sensory disabilities, such as books in Braille (Ajuwon & Chitiyo, 2016). Resources for severe DD, which lead to more complex needs, are limited, as reported for example in South Africa (UNESCO Global Education Monitoring Report Team, 2020) and Nigeria (Ajuwon & Chitiyo, 2016).

### Capturing stakeholder voices

Implementation science evidence shows that the successful implementation of any innovation is influenced by factors intrinsic to the innovation and the implementation process and by contextual factors at the individual, organisation and broader socio-political levels (Damschroder et al., 2009). The direct experiences of relevant stakeholders can provide important information on factors that facilitate or hinder innovations in general (Damschroder et al., 2009), and IE specifically (UNESCO Global Education Monitoring Report Team, 2020). In addition, understanding the potential opposition of some stakeholders to IE is needed to develop implementation strategies to overcome this potential barrier. For instance, DD in Africa are often considered a curse or punishment, leading community members and school staff to hold negative attitudes towards pupils with DD (Abosi, 2007; Stone-MacDonald, 2012; Uba & Nwoga, 2016). In turn, these can hinder effective inclusion (Abosi, 2007; Adera & Asimeng-Boahene, 2011), while its success requires the commitment of all stakeholders (Omede, 2016). A strong plan for implementing IE for pupils with DD in sub-Saharan Africa, therefore, requires appropriate consideration of the experiences, views and attitudes of diverse stakeholder groups.

A qualitative analysis of data from stakeholders in six sub-Saharan African countries reported favourable attitudes towards IE for children with disabilities more generally (Hui et al., 2018). However, overall a multifaceted picture emerges from the evidence on this topic. Children with disabilities and their parents in multiple countries appreciated the opportunities for inclusion and learning provided by IE, but also reported instances of peers’ bullying and teachers’ hostile attitudes (Asamoah et al., 2018; Bannink, Nalugya, & Van Hove, 2020; Brydges & Mkandawire, 2017, 2020; Leseyane et al., 2018; Magumise & Sefotho, 2020). Mainstream teachers, at times keen to promote education for all pupils (Asamoah et al., 2018; Franck & Joshi, 2017; Magumise & Sefotho, 2020; Mukhopadhyay, 2014), often felt they were not sufficiently trained for IE, or feared their teaching could be slowed down by children with disabilities (Asamoah et al., 2018; Chhabra et al., 2010; Franck & Joshi, 2017; Kuyini et al., 2020; Mukhopadhyay, 2014). Understanding whether a similar ambivalence exists when focusing on the inclusion of children with DD in specific can lead to better promotion of IE for this group.

### Aims and objectives

Attitudes, beliefs and experiences are captured in depth through qualitative research, which can provide novel information reflecting stakeholder perspectives. Reviewing and synthesising multiple qualitative studies can provide insights into themes that are recurrent across various samples from different countries and stakeholder groups (Thomas & Harden, 2008), setting the basis for context-appropriate recommendations to implement inclusive practices in sub-Saharan Africa. This systematic review aims to synthesise qualitative research on stakeholder experiences, attitudes and perspectives on the inclusion of pupils with DD in mainstream schools in sub-Saharan Africa. Specifically, the focus is on primary and secondary schools, typically grouped as a distinct category from other educational levels in global reports and targets on education access and inclusion (UNESCO Global Education Monitoring Report Team, 2020). The specific objectives of this review are:

1. To systematically identify qualitative studies on stakeholder experiences of and attitudes towards IE of pupils with DD and perspectives on implementation feasibility and barriers in primary and secondary schools in sub-Saharan Africa.
2. To critically appraise and synthesise results from the above studies, describing factors that can influence the successful implementation of IE for the target group.
3. Based on this synthesis, to provide recommendations for the promotion and implementation of inclusion of the target group in mainstream classrooms across sub-Saharan Africa.

## Method

The present review was conducted through systematic steps and reported following the *Enhancing transparency in reporting the synthesis of qualitative research (ENTREQ)* statement (Tong et al., 2012). Following the planning phase, including trial searches to define a comprehensive search strategy, a protocol was submitted to *PROSPERO* (National Institute for Health Research, n.d.) on 5 June 2020 and published online on 9 July 2020 (CRD42020185486).

### Systematic search

The search was run in health and education databases – PsycInfo (Ovid), MEDLINE (Ovid), Embase (Ovid), Global Health (Ovid), ERIC (Ebsco) – on 3 July 2020, then updated on 14 September 2021. Only peer-reviewed journals were searched in the databases that allowed this option (PsycInfo, ERIC) and any non-peer-reviewed publications were removed in the first screening stage when needed. No language or date restrictions were imposed.

The search strategy aimed to identify papers that combined four concepts, for which relevant keywords were identified through extensive brainstorming and consultation of similar reviews:

1. experiences, attitudes, perspectives and generally qualitative data;
2. education;
3. DD and related concepts;
4. sub-Saharan African countries.

In this review, ‘DD’ refers to intellectual disabilities, autism, attention-deficit hyperactivity disorder, language and social communication disorders, and extends to developmental delays, in which substantial problems in cognitive and/or behavioural development have been identified although not formally diagnosed. These definitions are in line with the forthcoming WHO UNICEF Global Report on Developmental Delays and Disabilities. It was decided to include ‘disability’ and ‘special needs’ among the keywords, to identify all potentially relevant studies, as general terms are often preferred to naming specific conditions in education research abstracts.

The full search strategy is available in *Supplementary Material A*. After screening (detailed below), forwards and backwards citation checks were carried out for all studies selected, to identify studies that were missed in the database search.

### Selection of studies

Authors EG and LN each independently reviewed titles and abstracts of all studies identified through the search, to select potentially relevant ones. Disagreement was resolved through discussions between the reviewers. Both reviewers then evaluated the full text of all articles selected, to identify those that met all inclusion criteria. Results were discussed with a third researcher (RAH), who resolved disagreements on three studies (2.2%).

We included primary research studies conducted at least in part in sub-Saharan Africa, which used qualitative methods to investigate stakeholder experiences, perspectives and attitudes towards the IE of pupils with DD in primary and secondary schools. We excluded studies centred around physical or sensory disabilities, or specific learning disabilities, such as dyslexia, as these require different provisions compared to DD. We included studies about IE of pupils with SEN in general, where participants or setting information indicated that at least 40% of participants had the experience of DD (directly, or indirectly through teaching or caregiving responsibilities). Full inclusion and exclusion criteria are reported in *Supplementary Material B*.

### Quality appraisal

EG and CJ appraised the quality of the studies selected using the *Critical Appraisal Skills Programme* (CASP, 2018) checklist for qualitative studies. Disagreement and uncertainty on 4% of decisions were resolved through discussions between the raters and CH. Quality appraisal was not used to exclude studies, but rather for assessment of the results presented and comparisons across studies.

### Data extraction

For each study, EG extracted, through a bespoke form, information on authors, publication year, aims and methodology, recruitment, sample size and characteristics, disorders considered, country, setting details, data collection and analysis methods and themes discussed. The Results and Discussion sections of each report were extracted and uploaded to the qualitative data analysis application NVivo-12 to be analysed.

### Synthesis

Thematic synthesis (Thomas & Harden, 2008) was applied to the studies, as this method is appropriate for synthesising qualitative studies which employed various methodologies. The analysis was rooted in critical realism, under the assumption of an existing reality, of which researchers aimed to analytically describe subjective stakeholder experiences. The approach chosen to develop analytical themes was template thematic analysis (Brooks et al., 2015).

EG first familiarised herself with the data, then coded meaningful sections with descriptive codes. Codes were iteratively revised and interpreted reflexively throughout. A coding template was generated deductively organising initial codes according to the *Consolidated Framework for Implementation Research* (CFIR; Damschroder et al., 2009). The CFIR aims to organise data on attitudinal and contextual factors surrounding the implementation of diverse innovations, to guide implementation efforts. It includes five domains relative to the intervention, outer setting, inner setting, individuals involved and implementation process (Damschroder et al., 2009). This framework was selected to organise stakeholder experiences and perspectives on factors influencing IE in a comprehensive way that would elicit targeted recommendations for implementing IE for pupils with DD.

Although the analysis was theory-driven and conducted systematically, there was no intention to eliminate subjectivity. In line with principles of reflexive thematic analysis (Braun & Clarke, 2019), applicable to the flexible template analysis (Brooks et al., 2015), subjective interpretation, appropriately recognised and documented, was considered a strength of the synthesis. Throughout the analysis, the main investigator was conscious of her own assumptions: a belief in the rights of persons with disabilities and positive attitudes towards IE and its implementation in low-resource settings.

After analysing the content of themes, the studies were grouped according to various features, including quality, stakeholder group and country, to compare them and to provide a richer account of factors influencing stakeholders’ views.

### Community involvement statement

Community stakeholders were not directly involved in this work. This review was nonetheless informed by needs expressed by parents of children with DD in our previous studies in sub-Saharan Africa (Tekola et al., 2020; Tilahun et al., 2016), and in interactive stakeholder meetings organised in Addis Ababa in 2019 and 2021. Lack of access to appropriate education (if not necessarily IE) is a key service gap according to parents (Tekola et al., 2020; Tilahun et al., 2016), and was one of the most prominent concerns expressed by parents of children with DD at two meetings on autism in Africa (Stellenbosch 2017 and Durban 2019) attended by the last author of this review.

## Results

### Study selection process and results

**Figure 1.**
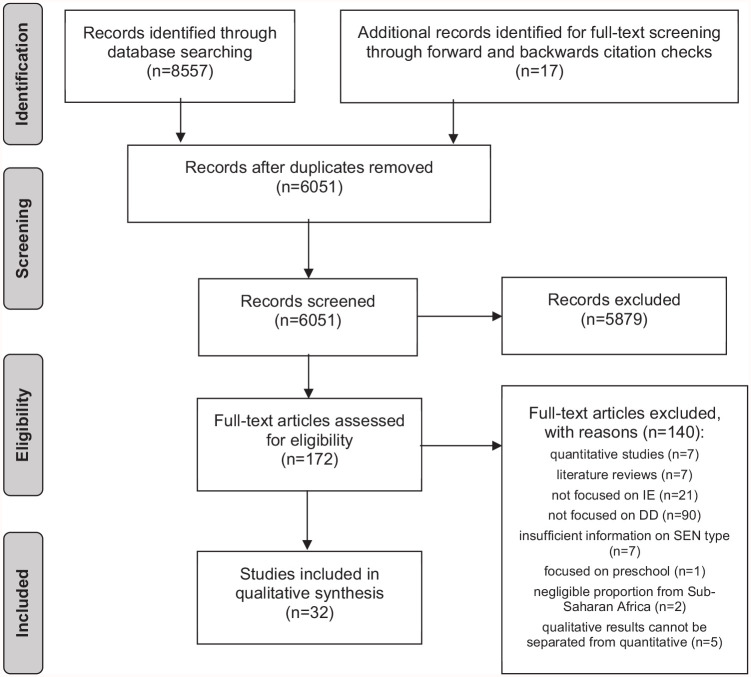
*PRISMA* flow diagram (Moher et al., 2009) of the study selection process.

A total of 32 articles, including 29 qualitative and 3 mixed-methods studies, were selected (see *PRISMA* flow diagram Moher et al., 2009, in Figure 1).

Of the 32 studies selected, 14 were conducted in South Africa, 5 in Zimbabwe, 4 in Botswana, 3 in Ghana, 3 in Uganda, 2 in Nigeria and 1 in Eswatini, all between 2001 and 2020. With some studies including multiple stakeholders, teachers’ experiences were explored in 26 studies, parents’ views in 7, pupils’ experiences in 6 directly and 2 through observation and informants’ reports.

In general, the research selected was deemed good quality in both methods and reporting. Common weaknesses in the studies included a lack of reflection on the relationship between researchers and participants and limited descriptions of the analysis approach. In a few instances, small samples sizes were not justified, or the analysis did not appear rigorous or was based entirely on super-imposed categories (e.g. interview questions) or quantitative entities (e.g. percentages of positive and negative responses).

Table 1 details the main study features and quality judgements. Demographic information was not recorded, due to the scarcity and variability of information reported in the studies. Full data extraction forms and a summary table of critical appraisal are available in *Supplementary Materials C* and *D*, respectively.

**Table 1.** Characteristics of included studies.

First Author	Country	Topic of Interest Relative to IE	Disorder of Interest	Methodology	Recruitment	Participants	School Level	Data Collection	Data Analysis	Quality
Alhassan and Abosi (2017)	Ghana	Teachers’ competence in curriculum adaptations	LD	Mixed-method, Phenomenology	Purposive	10 general teachers	Primary	Interviews, FGDs	IPA	Average
Bannink et al. (2016)	Uganda	Accessibility and inclusion	Spina bifida, including ID	Mixed-method	Purposive	30 general teachers (22 female), 30 pupils with SEN (12 female), 30 caregivers (27 mothers)	Primary (and nursery)	Observation, interviews	Thematic	Good
Bannink, Nalugya, Kawesa, et al. (2020)	Uganda	Intervention development and testing	SEN, including DD	Participatory action research, case studies, visual and oral narratives	Purposive	64 parents, 33 general teachers, 32 pupils with SEN, 32 peers	Primary	Interviews, FGDs, observation, PAR meetings	Thematic, Framework	Excellent
Bannink, Nalugya, and Van Hove (2020)	Uganda	Indigenous explanations and frameworks	DD	Qualitative, exploratory	Purposive, Typical cases	9 parents (8 mothers)	Primary	Interviews (based on cases observation, workshops)	Thematic	Very Good
Brydges and Mkandawire (2020)	Nigeria	Parents’ experiences and perceptions	SEN, including DD	Phenomenological	Purposive, Snowballing, Convenience	12 parents (10 mothers)	Primary	Interviews	Thematic, Content	Good
De Jager (2011)	South Africa	Pupils’ sensory processing difficulties	ASD	Case studies	Purposive	2 pupils with ASD (male; 5-7 years), 2 general teachers, 1 psychologist	Primary	Case observation, informant interviews	Thematic	Average
De Jager and Condy (2017)	South Africa	Pupil’s executive function and behavioural adaptation	ASD	Interpretative case study	Purposive	1 pupil with ASD (male; 9 years), 1 general teacher	Primary	Case observation, informant interview	Thematic, Interpretative	Good
Engelbrecht et al. (2001)	South Africa	Teachers’ stressors and coping skills	ID	Qualitative (NS)	Purposive	10 general teachers (female)	Primary	Questionnaire, observation, interviews	Thematic	Very Good
Engelbrecht et al. (2003)	South Africa	Teachers’ stressors	ID	Mixed method	Purposive	10 general teachers	Primary, Secondary	Interviews	Thematic, Framework	Good
Lopes et al. (2009)	South Africa	Teachers’ experiences	ADHD	Narrative design	Purposive, Convenience	17 general teachers (female)	N/A	Interviews	Thematic, Narrative	Excellent
Majoko (2016)	Zimbabwe	Barriers and enablers	ASD	Qualitative (NS)	Purposive	21 general teachers (12 female; 27–65 years)	Primary	Interviews	Thematic, Cross-case	Average
Majoko (2017)	Zimbabwe	Teachers’ support practices	ASD	Phenomenological case studies	Purposive	18 general teachers (11 female; 31–57 years)	Primary	Observation, document analysis, interviews	Thematic	Very Good
Majoko (2018)	Zimbabwe	Teachers’ experiences and competence	ASD	Phenomenology	Purposive	24 general teachers (9 female)	Primary	Interviews	Thematic, Constant-comparison	Very Good
Majoko (2019)	Zimbabwe	Teachers’ views on key competences	SEN, including ADHD, LD, EBD	Interpretative design	Purposive	24 general teachers (6 female)	Primary	Interviews	Thematic, Cross-case	Very Good
Mangope (2017)	Botswana	Teaching strategies	ID	Interpretative multiple case study design	Purposive	8 special teachers	Primary	Observation, interviews, document analysis	Thematic, Content Constant-comparison	Average
Mangope et al. (2018)	Botswana	Teaching practice experiences	22% ID, 48% LD	Phenomenology	Purposive	23 pre-service special teachers	Secondary	Interviews, FGDs	Thematic	Very Good
Mapuranga et al. (2015)	Zimbabwe	Impact on rights	ID	Qualitative (NS)	Random	10 general teachers, 10 parents, 10 pupils with ID	Secondary	Interviews, questionnaires	N/A	Very Poor
Matsenjwa et al. (2020)	Eswatini	Teachers’ experiences collaborating with parents	ID	Phenomenology	Purposive, rich cases	24 special teachers (15 female)	Primary	Interviews, FGDs	Thematic	Average
Mohamed and Laher (2012)	South Africa	Teachers’ understanding of LD	LD	Qualitative (NS)	Convenience	8 general teachers (female)	Primary	Interviews	Thematic, Reflexive	Average
Mokobane (2011)	South Africa	Pupils’ engagement	ID	Case study	Purposive	10 general teachers, 10 pupils with ID	Secondary	Interviews, FGDs	Spiral	Poor
Mukhopadhyay et al. (2019)	Botswana	Pupils’ experiences	19.4% ID, 25% LSI	Qualitative (NS)	Purposive	36 pupils with SEN (8–14 years), 36 without SEN	Primary	Observation, FGDs	Thematic, Constant-comparison	Good
Ngcobo and Muthukrishna (2011)	South Africa	Pupils’ experiences	78.5% LD, 1% ASD, 3% ID	Qualitative (NS)	N/A	1 principal, 5 general teachers	Primary	Observation, document analysis, interviews	Thematic	Average
Okyere et al. (2019a)	Ghana	Pupils’ experiences	DD	Descriptive design	Purposive	16 pupils with DD (8 female)	N/A	Observation, draw-and-write, interviews	Thematic	Very Good
Okyere et al. (2019b)	Ghana	Teachers’ experiences	DD	Descriptive design	Purposive	16 general teachers, 2 special teachers	Primary	Interviews	Thematic, Reflexive	Very Good
Otukile-Mongwaketse et al. (2016)	Botswana	Teachers’ understanding of curriculum adaptation	LD	Qualitative (NS)	Purposive	12 general teachers (30–48 years)	Primary	Observation, interviews	Thematic	Good
Potgieter-Groot et al. (2012)	South Africa	Training programme development	EBD	Action research	Purposive	35 general teachers, 14 school staff (40 female)	Primary	FGDs, observation, feedback forms	Thematic, Reflexive	Very Good
Seabi (2010)	South Africa	Teachers’ perceptions of ADHD	ADHD	Qualitative (NS)	Convenience	5 general teachers	Primary	Interviews	Thematic, Small q	Poor
Uba and Nwoga (2016)	Nigeria	Parents’ interpretation of stigma	ASD, ADHD, ID, LSI	Narrative design	Convenience, Snowballing	8 mothers	Primary, Secondary	Interviews	Thematic, Reflexive	Good
Van Schalkwyk and Marais (2017)	South Africa	Teacher-pupil relations	FASD	Qualitative (NS)	Purposive	14 general teachers (11 female)	Primary	Interviews, FGDs	Thematic, Reflexive	Good
Walton and Rusznyak (2014)	South Africa	Special-school teaching practice learning	SEN, including LD, ID	Interpretative design	Convenience	15 pre-service teachers (14 female)	Primary, Secondary	FGDs	Thematic, Framework	Excellent
Yoro et al. (2020)	South Africa	Teachers’ understanding	DD (mostly ADHD)	Interpretative design	Purposive	6 general teachers (5 female, 22–28 years)	Secondary	Observation, interviews, incident reports	Thematic, Reflexive	Good
Yssel et al. (2007)	South Africa (and USA)	Parents’ perceptions	SEN, including ASD, ID, LD	Qualitative (NS)	Convenience	32 South African parents (25 mothers), 10 American mothers	Primary, Secondary (and 5% Preschool)	FGDs	Thematic, Constant-comparison	Poor

Abbreviations: ADHD: attention-deficit hyperactivity disorder; ASD: autism spectrum disorder; DD: developmental disorder (not specified); EBD: emotional/behavioural difficulty/disorder; FASD: foetal alcohol spectrum disorders; FGDs: focus group discussions; ID: intellectual disability; IPA: interpretative phenomenological analysis; LD: learning difficulty/disability; LSI: language/speech impairment; N/A: not available; NS: not specified; SEN: special education needs.

### Synthesis

The codes generated through the thematic synthesis of the Results and Discussion sections of the selected studies mostly fitted in with CFIR domains (Damschroder et al., 2009). Aligning this review with the terminology used in CFIR, ‘inclusive education’ is the innovation, and mainstream schools are the implementation setting of interest. Following the CFIR, five themes were developed (see Table 2); a codebook summary is provided in *Supplementary Material E* and all coded extracts are available upon request. Each of the themes will be discussed in turn. Example quotes from the studies are reported in inverted commas, with words from participants or documents in italics.

**Table 2.** Overview of relevant constructs of the *Consolidated Framework for Implementation Research* (Damschroder et al., 2009) and corresponding themes and subthemes.

CFIR constructs	Short description of CFIR constructs relevant to the analysis	Corresponding themes and subthemes developed in this study
Intervention characteristics	Features of the innovation to be implemented	1 Framing of IE
Intervention source	Perspectives on the internal or external nature of source of the innovation	1.1 IE as a mandated practice
Evidence strength and quality		
Relative advantage	Perceived advantage compared to alternatives	1.2 Benefits of IE
Adaptability		
Trialability		
Complexity		
Design Quality and Packaging		
Outer setting	Socio-political factors and external inputs	2 Context for inclusion
Patient needs and resources	The target group’s needs and whether they are known within the implementation setting	2.1 Needs of children with DD
Cosmopolitanism		
Peer pressure		
External policy and incentives	Resources, including policy, guidance, supervision and incentives, provided by governments or other external organisations to facilitate implementing the innovation	2.2 Policy & incentives for IE
Inner setting	Structural, cultural and organisational factors internal to the implementation setting	3 Barriers and facilitators for inclusion within mainstream schools
Structural characteristics		
Networks and communications	Quality and extent of formal and informal internal communications	3.1 Within-school interactions
Culture	Norms, values and common perspectives within the implementation setting	3.2 School culture
Implementation climate	Internal factors that directly facilitate or hinder the implementation process	3.3 School climate for IE implementation:
*Tension for change*	Staff’s perceptions that the current situation needs improvement	*3.3.1 Teachers’ longing for improvement*
*Compatibility*	The extent to which the innovation is perceived as compatible with current organisational norms, workflow and staff’s needs	*3.3.2 IE compatibility with mainstream school practice*
*Relative priority*		
*Organisational incentives and rewards*		
*Goals and feedback*		
*Learning climate*		
Readiness for implementation	Tangible indicators of preparedness to implement	3.4 Availability of resources & training
*Leadership engagement*		
*Available resources*		
*Access to knowledge and information*		
		3.5 Inner-inner setting: inclusion in the classroom^a^
Characteristics of individuals	Organisational features measured at the individual level	4 Relevant features of teachers
Knowledge and beliefs about the intervention	Individuals’ attitudes towards and understanding of the innovation	4.1 Teachers’ knowledge & beliefs about inclusion
Self-efficacy	Individuals’ confidence in their ability to implement the innovation	4.2 Teachers’ skills and confidence for IE
Individual stage of change	Progression towards full ability for implementation	
Individual identification with organisation		
Other personal attributes		4.3 Teachers’ relational patterns with pupils
Process	Key activities for implementation	
Planning		
Engaging	Engaging stakeholders	5 Engaging stakeholders to implement IE
*Opinion leaders*		
*Formally appointed internal implementation leaders*		
*Champions*		
*External change agents*		
Executing		
Reflecting and evaluating		

CFIR: Consolidated Framework for Implementation Research.

a Given the nature of the school environment and through the analysis of data, a further layer of contextual information, referring to the classroom setting, has been added here compared to the *Consolidated Framework for Implementation Research*.

### Framing of IE

This theme refers to the core characteristics of IE as reported in the studies reviewed. IE was framed in the studies reviewed as ‘commitment to enhance the achievement of all children while safeguarding the inclusion of those who are vulnerable’ (Majoko, 2018). The goal ‘to take into account the differences and needs of all the learners’ (Mukhopadhyay et al., 2019) was mentioned across studies and supported by relevant education literature cited in the studies reviewed, which was used as theoretical contextualisation of stakeholders’ views. Discourses around IE focussed on its compliance with human rights and policy and its benefits to pupils with DD.

#### IE as a mandated practice

IE could be perceived as developed within the schools or imposed from an external source. Participants in, and authors of, the studies reviewed primarily considered the source of IE external to schools, mandated by policy.

‘Since Zimbabwe adopted inclusive education in 1994 in alignment with the global arena, learners with special needs, including autism, learn in regular classes’ (Majoko, 2017)

‘*Due to my social, cultural, moral and legal obligation to include all children in my classroom, I finally accepted him*’ (teacher; Majoko, 2019)

The co-existing internal, ‘moral’, source of IE, identified in the above quote, was also reported by other members of various stakeholder groups, who acknowledged that IE is a right for children with SEN (Mapuranga et al., 2015; Mukhopadhyay et al., 2019).

Two recent studies expressed that, according to indigenous frameworks of IE, children with disabilities had to deserve inclusion by showing they had the ‘ability to manage’ (Bannink, Nalugya, & Van Hove, 2020) and to ‘adapt to the challenges of learning alongside other students’ (Brydges & Mkandawire, 2020). However, one of them (Bannink, Nalugya, & Van Hove, 2020) highlighted that stakeholders resorted to human rights frameworks to understand the need to include the children whose disabilities did not allow them to fit in the indigenous framework.

#### Benefits of IE

Stakeholders discussed the advantages of IE compared to its alternatives – mainly segregated special education. Critically, studies exploring the views of pupils with DD suggested their preference for learning with typically developing peers in mainstream classrooms (Bannink et al., 2016; Mukhopadhyay et al., 2019; Okyere et al., 2019a), where they could progress academically:

‘*I am now going to be able to move to secondary school like my friends, unlike growing old in the* [special] *unit classroom*’ (learner with SEN; Mukhopadhyay et al., 2019)

South African parents explained children’s preference with their ‘determination to be like everybody else’ (Yssel et al., 2007). Parents equally ‘desired to raise their children as *normally* as possible’ (Yssel et al., 2007), although their preferences seemed more nuanced and dependent on the quality of inclusion. Multiple studies discussed how poor inclusion, when children with DD are stigmatised and their needs ignored, leads to experiences of discrimination, underachievement and a ‘variety of negative outcomes such as loss of self-esteem and poor motivation’ (Alhassan & Abosi, 2017).

Nonetheless, successful IE was consistently reported to promote children’s integration in the community, socialisation with their peers and reduced discrimination, compared to segregated education:

‘Teacher application of a universal approach to the management of obsessions and compulsions potentially eliminated typically developing learners’ stigmatization of their peers with autism’. (Majoko, 2017)

### Context for inclusion

#### Needs of children with DD

Teachers, learners and observers reported various needs of children with DD, particularly relating to learning and attention challenges, poor functional development, emotional and social difficulties, sensory challenges and rigidity. The most frequently reported challenges for children with any DD were learning and attention difficulties. About these pupils, South African teachers said:

‘*There are some kids who have difficulty understanding basic concepts, you know it takes long to understand a simple concept that you teach them.’..* (Mohamed & Laher, 2012)

‘*He cannot pay attention in class or finish his work*’. (Engelbrecht et al., 2003)

Pupils with DD themselves reported struggling with their attention and learning:

‘*When teacher is talking it is difficult to listen and understand so I look in the window*’ (pupil with DD; Okyere et al., 2019a)

Five studies on IE of pupils with ASD (De Jager, 2011; De Jager & Condy, 2017; Majoko, 2016, 2017, 2018, 2019) reported barriers to their inclusion that were not discussed for other disorders, specifically challenging sensory experiences and rigidity of thought, interests and routines. For example, in one study, the majority of teachers recognised that:

‘*Children with ASD have complication transitioning from one academic or social activity or environment to another*’ (Majoko, 2016)

As in the above excerpt, some South African and Zimbabwean teachers’ demonstrated understanding of autism and other DD. However, more often, their knowledge was limited and their beliefs diverged from scientific evidence, particularly on aetiology and treatment needs. For example, a ‘*poor diet*’ (Seabi, 2010) was considered a cause of ADHD.

Conversely, teachers were aware of additional needs that pupils with DD face in the community, which impose further barriers to their learning. Environmental factors such as poverty and unsupportive parenting were mentioned as concurrent causes of learning challenges.

#### Policy and incentives for IE

Barriers to implementing IE policies were reported in all seven countries of the synthesised studies. Governments were accused of not prioritising IE (Mapuranga et al., 2015; Okyere et al., 2019b) and of producing poorly defined IE policies. In South Africa, various policies aim at providing specific indications for effective inclusion, such as Outcomes-Based Education, the creation of School-Based Support Teams and District-Based Support Teams (Mohamed & Laher, 2012; Ngcobo & Muthukrishna, 2011). However, they appear scarcely implemented: teachers in several South African studies seemed unaware that ‘Department of Education (2005: 15) states: *taking learners out of classes should be reduced to a minimum*’ (Mohamed & Laher, 2012); moreover, relevant support teams did not assist teachers:

‘*PGSES [Psychological, Guidance and Special Education Services] does not visit our school … they have never visited our school … you call them, they do not come*’ (teacher; Ngcobo & Muthukrishna, 2011)

Insufficient support was also reported in a Ghanaian rural school, where education officers ‘*don’t observe how the teachers teach*’ as ‘*they don’t care*’ (Alhassan & Abosi, 2017). Combined with low wages and incentives, the lack of supervision in the school leads to uncommitted teachers working short hours and taking ‘*the freedom to arrange or teach school subjects in a manner that suit them*’ (Alhassan & Abosi, 2017).

The only support and in-service training for IE mentioned in studies based in Ghana were offered by NGOs (Alhassan & Abosi, 2017; Okyere et al., 2019b). Some references to training provided by the Department of Education were made in Zimbabwe (Majoko, 2018) and South Africa (Lopes et al., 2009; Potgieter-Groot et al., 2012). However, although these studies were of high quality and based in schools with diverse socio-economic backgrounds, even in the above countries the training availability mentioned is not universal: in some South African semi-rural communities, teachers are ‘in dire need of professional development’ (Ngcobo & Muthukrishna, 2011).

### Barriers and facilitators for inclusion within mainstream schools

#### Within-school interactions

Collaboration and reciprocal support between teachers were presented as IE facilitators in 11 studies. Ten of these described positive interactions, which helped teachers overcome psychological and practical challenges faced in inclusive classrooms:

‘They appreciated peer support and shared instructional strategies they employed to make inclusion work for students with IDD [DD]’ (Okyere et al., 2019b)

Two studies in South Africa (Mohamed & Laher, 2012; Potgieter-Groot et al., 2012) and one in Zimbabwe (Majoko, 2017) reported formal sharing of knowledge and strategies across all teachers, promoting a whole-school approach to inclusion:

‘The teachers started a working group to develop specific support plans for each learner experiencing barriers to learning’ (Potgieter-Groot et al., 2012)

Five studies indicated that support by special needs educators and teaching assistants in the classroom was particularly valued:

‘When asked about a time when delivering education to students with IDD [DD] in the general education classroom together with non-disabled peers went really well, some participants (n=5) discussed support from special educators’. (Okyere et al., 2019b)

However, study results also suggest that collaboration with special educators is generally insufficient, as these are scarcely available (Bannink et al., 2016; Engelbrecht et al., 2001, 2003; Mangope, 2017; Okyere et al., 2019b). Poor collaboration among teachers was also reported in one South African school, where ‘teachers in whose classes disabled children were integrated received very little support from their colleagues’ (Ngcobo & Muthukrishna, 2011).

#### School culture

Inclusive school cultures were restricted to few cases, such as the whole-school approaches described above and ‘schools with a history of practicing inclusive education’ (Mukhopadhyay et al., 2019). Most often, school environments reflected histories of segregation and negative attitudes towards disability. Due to stigma towards people with disabilities in their communities, parents in South Africa, Uganda and Nigeria reported mainstream schools denying inclusion of their children with SEN (Bannink et al., 2016; Brydges & Mkandawire, 2020; Uba & Nwoga, 2016; Yssel et al., 2007). Moreover, teachers and pre-service teachers in various studies displayed fear and negative effects towards pupils with DD. In one primary school in Botswana, teachers influenced typically developing pupils to bully peers with SEN:

‘Some teachers [. . .] do not call learners with SEN by their names; they call them names such as *old woman, old man, uncle, and aunt*. Such attitude then goes to some learners, as they also would think that such names were acceptable’. (Mukhopadhyay et al., 2019)

Discourses of teachers exhibiting positive attitudes were usually still permeated with the cultural dichotomy between normal and abnormal, in contrast with the social model of disability foundational to IE (UN General Assembly, 2007). They discussed being ‘*only trained to teach normal children*’ (Engelbrecht et al., 2003), and suggested that children with SEN should be ‘*improved*’ (Ngcobo & Muthukrishna, 2011). Similar school cultures offer ‘limited opportunities for the development of a positive disabled identity’ (Ngcobo & Muthukrishna, 2011).

Many studies also described exclusionary practices towards children with DD in mainstream schools, often enacted out of lack of experience:

‘Many of the teachers did not know how to deal with learners’ problem behaviour and often send them out of the class or to the principal’s office’. (Potgieter-Groot et al., 2012)

Two studies in Ghana (Okyere et al., 2019a, 2019b), one in Nigeria (Brydges & Mkandawire, 2020) and one in Zimbabwe (Mapuranga et al., 2015) reported the use of corporal punishment, ‘disproportionally targeted’ (Okyere et al., 2019a) at children with DD due to behavioural and learning challenges:

*‘When am not able to answer question madam will cane us or when someone answers madam will say the boy who got the answer correct should take the cane and cane the people who are standing’* (pupil with DD; Okyere et al., 2019a)

#### School climate for IE implementation

##### Teachers’ longing for improvement

Opposing the negative school cultures presented, 13 studies revealed that teachers perceived a need for change in the current education provision, as they recognised ‘the importance of including diverse learners in their classrooms’ (Yoro et al., 2020). South African student teachers who attended teaching practice in both special and mainstream schools were critical towards segregated education, as well as exclusionary practices within mainstream schools:

‘Surita bemoaned her observation of how teachers in a mainstream school sent a student out of the class for poor academic performance by asking *What is leaving him outside going to do to [help] him?*’ (Walton & Rusznyak, 2014)

Teachers in mainstream schools expressed and demonstrated their efforts to include pupils with DD, recognising that learning is important for the future of pupils with DD as of their peers, as pupils themselves (Mukhopadhyay et al., 2019; Okyere et al., 2019a) and their parents (Bannink, Nalugya, & Van Hove, 2020; Brydges & Mkandawire, 2020; Uba & Nwoga, 2016; Yssel et al., 2007) highlighted:

‘Teachers are trying hard to come to terms with the inclusion of intellectually challenged learners in inclusive schools and to support them’. (Mokobane, 2011)

##### IE compatibility with mainstream school practice

Across studies, accommodating the needs of pupils with DD was seen as requiring additional time and effort, incompatibly with the typical workflow of mainstream schools. Teachers lamented the lack of human resources, such as the limited availability of special educators who could assist them in teaching (Bannink et al., 2016; Engelbrecht et al., 2001, 2003; Mangope, 2017; Okyere et al., 2019b). In a South African school, ‘a parent hired a classroom assistant for her child at her own cost’ (Yssel et al., 2007). In several studies, participants explained that learners with DD require extra time and attention, for which teachers either work outside the regular timetable or neglect other pupils and sacrifice meeting teaching schedules:

‘The significance of this study was to show the severe stress the teachers experience in classes with FASD [foetal alcohol spectrum disorders] learners, due to the frustration of not having enough time to spend with the other children’ (Van Schalkwyk & Marais, 2017)

The above challenges were frequently discussed in relation to overcrowded classes, in which teachers are not able to effectively address individual needs:

‘*We are teaching 40 in a class, it is very difficult to teach an inclusive education class. I don’t think we are doing justice to the learner, I think we probably be choosing between other learners and this learner*’. (teacher; Mohamed & Laher, 2012)

Classroom setup and infrastructure challenges are additional barriers to teaching in general and IE in particular, as was the case of two classes being taught in the same room due to scarce availability of classrooms in a Ghanaian school (Alhassan & Abosi, 2017). In a study in Zimbabwe, all participating pupils, parents and teachers ‘indicated that there were no suitable facilities’ for children with intellectual disabilities (Mapuranga et al., 2015). However, such infrastructure challenges were not highlighted in South Africa.

#### Availability of resources and training

Mainstream schools appear poorly equipped with financial, material and training resources to implement effective IE. As well as inclusive infrastructure, they were reported to lack teaching equipment in all countries studied. In some instances, teachers and pupils bought materials at their expense:

‘The special educators reported feeling frustrated as they often invested personal resources into specific teaching and learning aids to support their teaching of students with IDD [DD]’ (Okyere et al., 2019b)

Despite a few mentions of beneficial training, the most frequently reported barrier to effective inclusion was unmet training needs, discussed in almost all studies. Some studies reported that pre-service training had not prepared teachers for IE of children with DD.

Two rigorous studies (Mangope et al., 2018; Walton & Rusznyak, 2014) specifically on teaching practice for student teachers, reported limitations of pre-service programmes, including inadequate mentorship. Student teachers specialising in SEN at the University of Botswana struggled in IE teaching practice, as the university programme, which focussed on single disabilities, had failed to prepare them for the variety of needs in inclusive classrooms (Mangope et al., 2018).

In-service training on IE is equally lacking in all countries where the studies were conducted and, when available, it often fails to provide specific skills and knowledge on DD.

#### Inner-inner setting: inclusion in the classroom

Given the nature of the school environment and through the analysis of data, a further layer of contextual information has been added here compared to the CFIR (Damschroder et al., 2009). While the school as a whole is the *Inner Setting*, this theme explores the classroom environment in more depth.

In line with school cultures presented earlier, 11 studies discussed exclusionary practices within classrooms. In some instances, teachers did not attempt to involve pupils with DD, when they spontaneously disengaged from classroom activities and ‘missed the learning experience’ (De Jager, 2011). Furthermore, two studies in South Africa and one in Botswana described teachers grouping children according to ability, creating stratifications that discriminated against weaker learners, including pupils with DD (Mangope, 2017; Ngcobo & Muthukrishna, 2011; Yoro et al., 2020).

Despite these examples, teachers’ attempts and strategies to promote a positive and inclusive environment were reported in 14 studies. They included de-cluttering the physical environment and reducing noise to address sensory needs and fostering inclusive relationships among pupils:

‘*Teachers need to educate other children and encourage them to communicate effectively with those who are different*’ (teacher; Mohamed & Laher, 2012)

Typically developing pupils supported their peers with DD spontaneously too, by providing emotional support and in one case even financial assistance to pay school fees (Okyere et al., 2019a), and by helping them to learn:

‘*My friends also help me to read and understand school work*’ (learner with DD; Okyere et al., 2019a)

As in the above quote, pupils with and without SEN reported being friends (Mukhopadhyay et al., 2019; Okyere et al., 2019a). Opposite views were expressed by South African parents stating that their children with SEN did not have real friendships:

‘*She is friendly with everybody* […] *But to have an intimate friend, build an intimate friendship, is very difficult for her*’ (Yssel et al., 2007)

Despite positive peer interactions, 10 studies also discussed how pupils with DD were at times bullied, teased and excluded by their peers:

‘The respondents further argued that the other learners in the school did not want to associate with children with ID as they sometimes give them degrading names such as [. . .] *morons or imbecile*’ (Mapuranga et al., 2015)

In addition, more than half studies discussed class disruption, aggressiveness and negative reactions to events displayed by some children with DD. Together with peer victimisation, these behaviours often made teachers uncomfortable and represented another threat to positive classroom environments and the inclusion of pupils with DD.

### Relevant features of teachers

#### Teachers’ knowledge and beliefs about inclusion

In several cases, teachers in South Africa, Zimbabwe and Botswana understood IE as the promotion of a positive environment for all learners and targeting individual needs, in line with definitions given in this review and in the studies synthesised. Teachers were able to identify key skills needed for inclusion, particularly the identification of disorders and needs, curriculum and task adaptations and behaviour management. However, studies in Ghana did not report similar reflections, and it was not unusual for teachers also in other countries to consider inclusion achieved whenever children with DD attended mainstream schools, regardless of how well their needs were met:

‘The majority of respondents [parents of children with DD] reported that [. . .] often, no allowances or considerations were made for the children’s varying needs’. (Uba & Nwoga, 2016)

In the studies synthesised, teachers presented an equal split between positive and negative attitudes towards IE. In general, it appears that while ‘many teachers are keen on the concept of inclusion’ (Okyere et al., 2019b), poor understanding of disability, limited time and resources and lack of skills and confidence hinder positive attitudes:

‘In many cases teachers indicated that learners who experience emotional and behaviour barriers are a burden and should not be part of mainstream schools: *This is not my job. I am a trained teacher and I am not qualified to identify and deal with problems of an emotional nature*’. (Potgieter-Groot et al., 2012)

#### Teachers’ skills and confidence for IE

Teachers exhibited different levels of skilfulness in IE and confidence in their ability to effectively include pupils with DD, often depending on their experience and the availability of training programmes.

Inadequate teachers’ skills and confidence for IE were reported in 15 studies across countries:

‘Teachers, on the other hand, felt they required more knowledge and skills to be able to include children with disabilities in school’ (Bannink, Nalugya, Kawesa, et al., 2020)

Nonetheless, there was a similar number of indications that teachers’ skills, confidence and attitudes towards inclusion could improve through training and, more frequently, experience.

‘Five of the teachers that had over 10 years of teaching experience felt that it was their experience rather than their training that helped them cope with learners with LD [learning disability/ difficulty]’ (Mohamed & Laher, 2012)

Most studies also described teachers’ acquired knowledge and use of strategies to include learners with DD and meet their individual needs, such as curriculum and task adaptation, slower teaching pace, collaborative learning, peer tutoring, visual aids, reinforcement and direct teaching of social skills. Examining the specifics of such strategies is beyond the scope of this review; these will be the focus of another article.

#### Teachers’ relational patterns with pupils

Among teachers’ *Other Personal Attributes* (Damschroder et al., 2009), their relational styles towards pupils with DD were deemed especially relevant to IE. Relationships described by teachers and pupils were variable in quality: positive in eight studies, negative in six, more ambivalent in four.

Negative relationships were usually considered a consequence of teachers’ reactions to pupils’ disruptiveness or aggressiveness:

‘Continuing negative experiences and associated hindrances are a serious impediment to educators forming quality bonds with these learners. PP: *Children give so many problems that it is difficult to bond with them*’ (Van Schalkwyk & Marais, 2017)

Positive relationships, key to promoting inclusion, appear to be fostered by teachers’ empathy and compassion:

‘As the teachers become more empathetic and caring, they developed a willingness to build trusting and caring relationships with these learners’ (Potgieter-Groot et al., 2012)

### Engaging stakeholders to implement IE

Engaging stakeholders was discussed in almost all studies. While most studies focussed on teachers’ experiences, their collaboration across schools and with other stakeholders was considered crucial for successful IE.

The involvement of three main stakeholders was discussed: authorities, therapists and parents. It was recommended that governments supported schools and formulated appropriate policies where lacking, and that teachers gained skills to collaborate with authorities, to effectively implement IE. In addition, teachers and pre-service teachers valued and advocated for therapists’ collaboration, as a facilitator for inclusion:

‘The point of “outside help” was extended by Alice who suggested that a school could ask *a psychologist to come in once a week* and Sadie who felt that the role of the therapists in the special school she attended meant that *You don’t have to struggle as a teacher by yourself*’ (Walton & Rusznyak, 2014)

Across studies and countries, parents were presented as the most important teachers’ collaborators for successful IE. Teachers often reported helpful interactions with families, such as employing ‘*their* [parents’] *strategies to manage and communicate with children with autism*’ (Majoko, 2017). However, in one study, South African parents hinted at teachers’ hostility towards parents’ attempts to collaborate (Yssel et al., 2007). In South Africa (Engelbrecht et al., 2001, 2003; Mohamed & Laher, 2012), Botswana (Mangope, 2017), Eswatini (Matsenjwa et al., 2020) and Ghana (Okyere et al., 2019b), teachers reflected on their own negative experiences, whereby parents did not reach out to teachers or respond to their collaborative efforts:

‘*The parents just send their children to school and say its free education as if government will provide everything for the learner. [. . .] They do not work with us in supporting the learner to overcome his/her challenges*’ (teacher; Matsenjwa et al., 2020)

In three instances, teachers complained that sometimes parents reacted in denial to suggestions that their child might have SEN (Engelbrecht et al., 2001; Matsenjwa et al., 2020; Mohamed & Laher, 2012). Similarly, in Nigeria, ‘denial constituted a dominant way of coping for most mothers’ (Uba & Nwoga, 2016).

## Discussion

This systematic review aimed to synthesise stakeholder experiences, attitudes and perspectives on IE for pupils with DD in mainstream schools in sub-Saharan Africa, to explore factors that facilitate or hinder its effective implementation. The research area under review is a relatively recent field in sub-Saharan Africa, as all 32 studies identified were published after 2001, several years after 1994, when the UNESCO *Salamanca Statement* first advocated for IE (UNESCO, 1994). Generally, the reviewed studies presented rigorous methods and reporting. Results of poorer quality studies did not differ substantially from the former.

Through thematic synthesis, themes relative to all CFIR (Damschroder et al., 2009) domains were generated. In this review, *Intervention Characteristics* outlined how previous research in sub-Saharan Africa framed IE and identified its source in human rights and policy. The *Outer Setting* presented the needs of pupils with DD that schools must address for successful inclusion, and external support and guidance for IE. The *Inner Setting* described school environments, including within-school interactions, stigmatising and inclusive attitudes and practice and logistical barriers to IE under resources, training and work conditions. The analysis suggested that an additional level of contextual information within the *Inner Setting* is needed for school-based innovations: the inner-inner setting, describing the classroom environment and pupils’ interactions. *Characteristics of Individuals* outlined teachers’ understanding of and attitudes towards IE, their relations with pupils with DD and their skills and confidence in meeting pupils’ needs. In the *Process* domain, stakeholder engagement for implementing IE was discussed.

Regardless of contextual variations in countries and communities, the notable similarity of experiences was reported across studies in all domains. Despite frequently unclear and insufficient IE policy, stakeholders’ awareness of IE benefits, and particularly rights protection, provides a favourable basis for the inclusion of pupils with DD. However, barriers were reported at the outer-setting, inner-setting and individual levels. Crucially, as suggested by the theorisation of the CFIR (Damschroder et al., 2009), the analysis indicated that barriers interact across levels.

Pupils identified and diagnosed with DD in sub-Saharan Africa tend to be those whose disabilities have substantial impacts on their daily functioning. Pupils in the schools studied presented complex emotional, social and learning needs which mainstream teachers often felt unable to address. Consequently, even in the rare instances when they are included in mainstream classrooms, pupils with DD in sub-Saharan Africa still face some exclusion when they are not able to fully participate in the lesson with their peers. A review of studies conducted in other continents, mostly in high-income countries, elicited similar conclusions specifically concerning IE for pupils with autism: stakeholders reported as discriminatory the excessive reliance on assistants for instructing learners with autism, caused by teachers’ real or perceived lack of knowledge and skills to include them in their lesson (Roberts & Simpson, 2016). However, while in sub-Saharan Africa these experiences are common across DD, they may be more disorder-specific in high-income countries: for instance, a representative sample of Danish teachers demonstrated good knowledge of inclusive strategies for pupils with ADHD (Mohr-Jensen et al., 2019).

At the classroom level, reviewed studies reported pupils’ disruptiveness or aggressiveness as a cause for individual-level barriers to inclusion, namely negative teachers’ attitudes, low self-confidence and poorer teacher-pupil relationships. Similarly, quantitative studies in South Africa (Engelbrecht et al., 2003) and Botswana (Chhabra et al., 2010) found inappropriate and uncontrolled behaviour to be associated with teachers’ negative attitudes towards IE. In high-income countries, where similar challenges were reported, behaviour management responsibilities fall onto special educators (Roberts & Simpson, 2016), who are rarely available in sub-Saharan Africa (Engelbrecht et al., 2003; Okyere et al., 2019b).

At the school level, negative attitudes are fostered by cultural values based on community stigma towards DD, and a tradition of corporal punishment (Mapuranga et al., 2015; Okyere et al., 2019a; 2019b). The UNESCO *Global Education Monitoring Report 2020* identifies corporal punishment in schools as a global problem, of which vulnerable children are victims more often than their peers (UNESCO Global Education Monitoring Report Team, 2020). In line with the report, this review suggests that traditional behaviour management methods are due to large class sizes, scarce teachers’ skills, limited support from specialists and a lack of adequate infrastructure and resources, demonstrating low support at the outer-setting level, specifically from authorities.

Making sufficient human, financial and infrastructure resources available is a crucial long-term goal for effective and sustainable IE (UNESCO Global Education Monitoring Report Team, 2020). However, in line with previous research on including children with any disabilities (Asamoah et al., 2018; Chhabra et al., 2010; Franck & Joshi, 2017; Kuyini et al., 2020; Mukhopadhyay, 2014), a key first step identified in this review is addressing teachers’ training and supervision needs while also improving their work conditions and providing incentives. This was attempted in recent intervention studies in sub-Saharan Africa aimed at enhancing teachers’ practice (Conn, 2017; Evans & Acosta, 2020). As previously suggested (Okyere et al., 2019c), this review indicates that teachers frequently lack understanding of IE principles, such as adaptations to individual learning needs. Appointed supervisors are reportedly unsupportive and equally untrained (Alhassan & Abosi, 2017; Ngcobo & Muthukrishna, 2011; Okyere et al., 2019b). While pre-service training programmes need a clearer focus on inclusion and experiential knowledge of DD (Mangope et al., 2018; Walton & Rusznyak, 2014), IE in-service training programmes are largely unavailable, as confirmed by World Bank data (Wodon et al., 2018): IE skills are among the least taught in professional development in sub-Saharan Africa, where less than 10% of teachers have attended an IE training programme. This review further suggests that, when available, IE in-service training programmes fail to convey DD-specific knowledge and skills (Majoko, 2018; Seabi, 2010).

Conversely, a powerful opportunity to implement IE arises due to the willingness of teachers to include pupils with DD, previously highlighted for children with disabilities more generally (Asamoah et al., 2018; Franck & Joshi, 2017; Magumise & Sefotho, 2020; Mukhopadhyay, 2014), and to meet their learning, social and emotional needs. As a scoping review suggested (Okyere et al., 2019c), many teachers demonstrate some understanding of DD and IE strategies and the ability to foster inclusive environments, acquired mostly through experience. Collaboration between teachers, who share knowledge and help each other implement such strategies, can further promote inclusion.

The work of NGOs in sub-Saharan Africa is another opportunity for addressing training and support needs (Alhassan & Abosi, 2017; Okyere et al., 2019b). In line with implementation science (Damschroder et al., 2009; Powell et al., 2015), this review suggests that engaging all stakeholders is a key strategy for implementing IE. For instance, NGOs can provide in-service training programmes and resources. However, to ensure sustainability of IE, the main source of support should be national authorities. An analysis of education policies in Europe indicated that specific policies on IE and those including a clear definition of SEN and guidance on individualised learning outcomes, support for teachers and parental engagement, are key to promote IE for pupils with DD (van Kessel et al., 2021). This review suggests that sub-Saharan African governments are yet to formulate similar policies to support IE implementation.

### Limitations and future research

The main limitation to the results presented is the limited variety and comprehensiveness of settings and stakeholders. Most studies were conducted in the southern region of sub-Saharan Africa, with South Africa accounting for half of the research included, possibly limiting the transferability of results to other countries and communities. However, eastern and western regions were somewhat represented, and similarities across the studies synthesised suggest that comparable experiences may be reported in other sub-Saharan African countries.

Compared to teachers’ perspectives, experiences of learners with DD, their parents and other stakeholders were under-represented, leading to a partial account of IE for pupils with DD. This may be an issue in interpreting the synthesis, as teachers’ reported experiences and their observed practice may be subject to social desirability bias. In fact, some studies with teacher participants described a much more inclusive environment than those with pupil and parent participants. However, this difference could also be due to contextual changes across studies and, in general, teachers’ views appear nuanced and honest about IE challenges. Notably, when children and parents’ views were explored, participants were usually recruited from schools. Future research should explore attitudes towards mainstream education of the majority of children with DD in sub-Saharan Africa, who cannot access school or drop out, and their parents.

As only 25% of the studies reviewed included data on secondary schools, the account presented is likely to be more reflective of primary school settings. Globally, children with difficulties in cognition, communication and self-care are the least represented children with disabilities in secondary schools (UNICEF, 2021). Therefore, while some of the findings of this review will be relevant to secondary education, more research is needed to explore such disparity in sub-Saharan Africa and any potential additional barriers that may account for it.

While no language excluding criteria were applied in study selection, a limitation of our review methodology is the inability of the database search to comprehensively retrieve studies without an English title and/or abstract, as all keywords were in English. However, this limitation was mitigated at least in part using subject headings in Ovid databases and through forward and backward citation checks.

## Conclusion

The findings from this systematic review indicate that the context for IE of children with DD in sub-Saharan Africa presents multiple barriers and facilitators at the community, school, class and individual levels. To effectively implement IE for pupils with DD in the region, the authors recommend capitalising on facilitators, such as teachers’ will to promote inclusion and the efforts of NGOs. Key barriers that need to be addressed are the scarcity of equipment and training and supervision programmes within schools, as well as stigma towards DD in the community more broadly. Despite the limited financial resources available in the region, these implementation efforts should be prioritised, for compliance with international policy and protection of the rights of children with DD.

## Supplemental Material

sj-docx-1-aut-10.1177_13623613221096208 – Supplemental material for Stakeholder experiences, attitudes and perspectives on inclusive education for children with developmental disabilities in sub-Saharan Africa: A systematic review of qualitative studies Supplemental material, sj-docx-1-aut-10.1177_13623613221096208 for Stakeholder experiences, attitudes and perspectives on inclusive education for children with developmental disabilities in sub-Saharan Africa: A systematic review of qualitative studies by Elisa Genovesi, Cecilia Jakobsson, Lena Nugent, Charlotte Hanlon and Rosa A Hoekstra in Autism

sj-docx-2-aut-10.1177_13623613221096208 – Supplemental material for Stakeholder experiences, attitudes and perspectives on inclusive education for children with developmental disabilities in sub-Saharan Africa: A systematic review of qualitative studies Supplemental material, sj-docx-2-aut-10.1177_13623613221096208 for Stakeholder experiences, attitudes and perspectives on inclusive education for children with developmental disabilities in sub-Saharan Africa: A systematic review of qualitative studies by Elisa Genovesi, Cecilia Jakobsson, Lena Nugent, Charlotte Hanlon and Rosa A Hoekstra in Autism

sj-docx-3-aut-10.1177_13623613221096208 – Supplemental material for Stakeholder experiences, attitudes and perspectives on inclusive education for children with developmental disabilities in sub-Saharan Africa: A systematic review of qualitative studies Supplemental material, sj-docx-3-aut-10.1177_13623613221096208 for Stakeholder experiences, attitudes and perspectives on inclusive education for children with developmental disabilities in sub-Saharan Africa: A systematic review of qualitative studies by Elisa Genovesi, Cecilia Jakobsson, Lena Nugent, Charlotte Hanlon and Rosa A Hoekstra in Autism

sj-docx-4-aut-10.1177_13623613221096208 – Supplemental material for Stakeholder experiences, attitudes and perspectives on inclusive education for children with developmental disabilities in sub-Saharan Africa: A systematic review of qualitative studies Supplemental material, sj-docx-4-aut-10.1177_13623613221096208 for Stakeholder experiences, attitudes and perspectives on inclusive education for children with developmental disabilities in sub-Saharan Africa: A systematic review of qualitative studies by Elisa Genovesi, Cecilia Jakobsson, Lena Nugent, Charlotte Hanlon and Rosa A Hoekstra in Autism

sj-docx-5-aut-10.1177_13623613221096208 – Supplemental material for Stakeholder experiences, attitudes and perspectives on inclusive education for children with developmental disabilities in sub-Saharan Africa: A systematic review of qualitative studies Supplemental material, sj-docx-5-aut-10.1177_13623613221096208 for Stakeholder experiences, attitudes and perspectives on inclusive education for children with developmental disabilities in sub-Saharan Africa: A systematic review of qualitative studies by Elisa Genovesi, Cecilia Jakobsson, Lena Nugent, Charlotte Hanlon and Rosa A Hoekstra in Autism
