# Supplementary material for: Stakeholder experiences, attitudes and perspectives on inclusive education for children with developmental disabilities in sub-Saharan Africa: A systematic review of qualitative studies
Source: Autism. 2022 May 30;26(7):1606–25. doi: 10.1177/13623613221096208 (PMC9483198; doi:10.1177/13623613221096208)
Supplement: sj-docx-1-aut-10.1177_13623613221096208 – Supplemental material for Stakeholder experiences, attitudes and perspectives on inclusive education for children with developmental disabilities in sub-Saharan Africa: A systematic review of qualitative studies [file sj-docx-1-aut-10.1177_13623613221096208.docx]

## Supplementary Material A: Complete search strategy

Supplementary Materia A, Table 1. **Complete keyword search strategy in ERIC(Ebsco)**

| **Concepts** | **Keywords** |
| --- | --- |
| Experiences, attitudes, perspectives and generally qualitative data | qualitative or "mixed method" or view or views or perception or perceptions or perceive or perceived or voice or voices or experience or experiences or perspective or perspectives or attitude or attitudes or opinion or opinions or belief or beliefs or understanding or knowledge or awareness or stigma or prejudice or prejudices or stereotype or stereotypes or misconception or misconceptions or barrier or barriers or facilitator or facilitators or opportunity or opportunities |
| AND | |
| Education | school or schools or schooling or education or educational or class or classroom or classrooms or teacher or teachers or teaching |
| AND | |
| DD and related concepts | "developmental disorder" or "developmental disorders" or disability or disabilities or disabled or handicap or handicapped or "special education" or "special needs" or sen or "inclusive education" or "developmental delay" or "developmental delays" or "neurodevelopmental disorder" or "neurodevelopmental disorders" or autism or autistic or Asperger or "childhood disintegrative" or Rett or PDD or ASD or ADHD or "attention deficit" or hyperactive or hyperactivity or "language disorder" or "language disorders" or "language impairment" or "intellectual disorder" or "intellectual disorders" or "intellectual deficit" or "intellectual impairment" or "mental retard" or "mental retardation" or "mental deficiency" or "mental subnormality" or "mentally retarded" or "Down syndrome" or "trisomy 21" or mongolism or "fragile X" or "fetal alcohol" or "foetal alcohol" or foetalalcohol or FAS or FASD or "cerebral palsy" or "zika syndrome" or "Willliams syndrome" or "Tourette syndrome" or "Prader-Willi syndrome" or anencephaly or phenylketonuria or microcephaly |
| AND | |
| Sub-Saharan African countries^1^ | Africa or African or Sahara or Saharan or Angola or Benin or Botswana or "Burkina Faso" or Burundi or Cameroon or "Cape Verde" or "Cabo Verde" or Chad or Comoros or Congo or "Côte d'Ivoire" or "Ivory Coast" or Djibouti or Eritrea or Ethiopia or Gabon or Gabonese or Gambia or Ghana or Guinea or Kenya or Lesotho or Liberia or Madagascar or Malagasy or Malawi or Mali or Mauritania or Mauritius or Mozambique or Namibia or Niger or Nigeria or Réunion or Rwanda or Ruanda or "Sao Tome" or Senegal or Seychelles or "Sierra Leone" or Somalia or Somaliland or Sudan or Swaziland or Eswatini or Tanzania or Togo or Togolese or Uganda or Zambia or Zimbabwe or Rhodesia |

1. Keywords relative to Sub-Saharan Africa include a list of terms referring to all countries in the region. This was simplified by removing non-identifying words (e.g. “Republic of”) and avoiding repetitions of country names which included the same word (e.g. only “Sudan” was used for South Sudan and North Sudan). The list includes colonial names used into the 1970s, to allow for selection of older reports.

Supplementary Materia A, Table 2. **Complete keyword search strategy in Ovid databases (Embase, PsycInfo, MEDLINE, Global Health)**

| **Concepts** | **Keywords** |
| --- | --- |
| Experiences, attitudes, perspectives and generally qualitative data | qualitative or mixed methods or view* or perce* or voice* or experience* or perspective* or attitude* or opinion* or belie* or understand* or knowledge or awareness or stigma or prejudice* or stereotyp* or misconception* or barrier* or facilitator* or opportunit* |
| AND | |
| Education | school* or education* or class* or teach* |
| AND | |
| DD and related concepts | developmental disorder* or disab* or handicap* or special education* or special need* or sen or inclusive education or developmental delay* or neurodevelopmental disorder* or autis* or Asperger or childhood disintegrative or Rett or PDD or ASD or ADHD or attention deficit or hyperactiv* or language disorder* or language impairment* or intellectual disorder* or intellectual deficit* or intellectual impairment* or mental* retard* or mental deficien* or mental handicap* or mental subnormalit* or Down syndrome or mongolism or trisomy 21 or fragile X or f?etal alcohol or f?etalalcohol or FAS or FASD or cerebral pals* or zika syndrome or Willliams syndrome or Tourette syndrome or Prader-Willi syndrome or anencephaly or phenylketonuria or microcephaly |
| AND | |
| Sub-Saharan African countries^1^ | Africa* or Sahara* or Angola or Benin or Botswana or Burkina Faso or Burundi or Cameroon or Cape Verde or Cabo Verde or Chad or Comoros or Congo or Cote d'Ivoire or Ivory Coast or Djibouti or Eritrea or Ethiopia or Gabon or Gabonese or Gambia or Ghana or Guinea or Kenya or Lesotho or Liberia or Madagascar or Malagasy or Malawi or Mali or Mauritania or Mauritius or Mozambique or Namibia or Niger or Nigeria or Reunion or Rwanda or Ruanda or Sao Tome or Senegal or Seychelles or Sierra Leone or Somalia or Somaliland or Sudan or Swaziland or Eswatini or Tanzania or Togo or Togolese or Uganda or Zambia or Zimbabwe or Rhodesia |

1. See footnote 1 in Supplementary Materia A, Table 1

Supplementary Materia A, Table 3. **Subject headings (SH) used in Ovid databases (Embase, PsycInfo, MEDLINE, Global Health)**

| **Concepts** | **Embase SH** | **PsycInfo SH** | **MEDLINE SH** | **Global Health SH** |
| --- | --- | --- | --- | --- |
| Qualitative data | Exp qualitative research | Exp qualitative research | Exp qualitative research | N/A |
| Education | Exp education | Exp education | Exp education | Exp education  Exp schools |
| DD and related concepts | Exp autism  Exp attention deficit disorder  Exp developmental disorder  Exp mental deficiency  Exp language disability  Exp special education  Exp disability | Exp neurodevelopmental disorders  Exp language delay  Exp language disorder Exp special education Exp school integration Exp special education students  Exp disabilities | Exp neurodevelopmental disorders  Exp intellectual disability Exp language disorder Exp special education | Exp autism  Exp attention deficit hyperactivity disorder  Exp mental retardation  Exp people with speech impairments  Exp special education  Exp disabilities |
| Sub-Saharan African countries | Exp Africa  Exp “Africa south of the Sahara” | N/A | Exp Africa  Exp “Africa south of the Sahara” | Exp Africa |
