# Supplementary material for: Stakeholder experiences, attitudes and perspectives on inclusive education for children with developmental disabilities in sub-Saharan Africa: A systematic review of qualitative studies
Source: Autism. 2022 May 30;26(7):1606–25. doi: 10.1177/13623613221096208 (PMC9483198; doi:10.1177/13623613221096208)
Supplement: sj-docx-2-aut-10.1177_13623613221096208 – Supplemental material for Stakeholder experiences, attitudes and perspectives on inclusive education for children with developmental disabilities in sub-Saharan Africa: A systematic review of qualitative studies [file sj-docx-2-aut-10.1177_13623613221096208.docx]

## **Supplementary Material B: Inclusion and exclusion criteria**

| **Included** | **Excluded** |
| --- | --- |
| Primary research studies | Literature reviews, protocols, editorials, book chapters, documents not directly reporting primary research studies |
| Qualitative studies or studies with a discernible qualitative component | Mixed methods studies where the qualitative component could not be discerned from quantitative results and extracted |
| Studies conducted at least in part in Sub-Saharan Africa | Multi-country studies where data from Sub-Saharan Africa constituted a negligible proportion and could not be extracted separately |
| Studies on experiences of decision-makers, child mental health and rehabilitation specialists, special needs education experts, general and special education teachers, university students training for the above roles, school principals, disability organisations stakeholders, pupils with developmental disabilities (DD), typically-developing pupils, caregivers, the community | Studies not directly investigating stakeholders’ experiences, perspectives and attitudes |
| Studies about inclusive education (IE), or where a distinct section of findings on IE could be extracted | Studies about segregated education in special schools or classrooms |
| Studies about IE of pupils with DD or “learning disability”^1^ in primary and secondary schools | Studies focused on IE of preschool children or higher education students |
|  | Studies centred around physical or sensory disabilities, or specific learning disabilities, such as dyslexia |
| Studies about IE of pupils with special education needs (SEN) in general, where participant or setting information indicated that at least 40% of participants had experience of DD (directly, or indirectly through teaching or caregiving responsibilities). | Studies about IE of pupils with SEN in general, where relevance to DD could not be determined, even after contacting authors |

1 in Africa “learning disability” refers to any difficulties in learning which are independent of visible disabilities, a broad definition relevant to DD
