# Supplementary material for: Stakeholder experiences, attitudes and perspectives on inclusive education for children with developmental disabilities in sub-Saharan Africa: A systematic review of qualitative studies
Source: Autism. 2022 May 30;26(7):1606–25. doi: 10.1177/13623613221096208 (PMC9483198; doi:10.1177/13623613221096208)
Supplement: sj-docx-3-aut-10.1177_13623613221096208 – Supplemental material for Stakeholder experiences, attitudes and perspectives on inclusive education for children with developmental disabilities in sub-Saharan Africa: A systematic review of qualitative studies [file sj-docx-3-aut-10.1177_13623613221096208.docx]

## Supplementary Material C: Full data extraction forms

### **Note: List of abbreviations used in the extracts**

ADHD: Attention-Deficit Hyperactivity Disorder

AS: Asperger Syndrome

ASD: Autism Spectrum Disorder

DD: Developmental Disabilities

FASD: Foetal Alcohol Spectrum Disorder

ID/ IDs: Intellectual Disability

IDD: Intellectual and Developmental Disabilities

IE: Inclusive Education

NDD: Neurodevelopmental Disorders

SEN/ SENs: Special Education Needs

### **Alhassan & Abosi (2017)**

| **Full Reference** | Alhassan, A. R. K., & Abosi, C. O. (2017). Teachers' Pedagogical Competence in Adapting Curricula for Children with Learning Difficulties (LD) in Primary Schools in Ghana. *Journal of the American Academy of Special Education Professionals*, *41*, 61. |
| --- | --- |
| **Authors** | Alhassan, Abdul-Razak Kuyini  Abosi, Chigorom Okechukwu |
| **Year** | 2017 |
| **Country/ies** | Ghana |
| **Aim** | “The aim of this study is to investigate teachers’ pedagogical competence (TPC) in curriculum adaptation to include children with LD in primary schools.” |
| **Objectives** | “This study aimed to address the following objectives:  a) To find out the level of primary school teachers’ pedagogical competences in adapting  curricula to meet the needs of learners with LD in primary school. [quantitative phase]  b) To find out whether or not teachers’ pedagogical competency in curricula adaption is  associated with their skills in teaching children with LD. [quantitative phase]  c) To explore teachers experiences of adapting curricula to meet the needs of learners with  LD. [qualitative phase]” |
| **Research question(s)** | N/A |
| **Methodology** | “Mixed-Method Design was used. The underlying philosophical assumption for using mixed- method design was its dialectical position (Rocco, et al., 2003). This approach was required for a holistic comprehension and meaningful investigation of teachers’ competences in curricula adaptation. Accordingly, mixed design strategies (descriptive and phenomenology) were combined and carefully applied in the study.” |
| **Data collection methods** | “The interview guide dealt with teachers’ experiences of teaching children with LD in the general education classroom. […] All questions in the interview guide were not strictly followed during the interview. Teachers could stray from one topic, question or subject to another without being forced to stick strictly to the original plan in the interview guide. What was important was that the researchers were able to track the topical trajectories that got lost during the interview whenever those trajectories were considered appropriate and central to the subject matter (Turner, 2010; Cohen & Crabtree, 2006). The interview guide provided a great freedom and flexibility in sequencing the questions and in the amount of time and attention given to different topics and interviewees.” […]  “The qualitative data were generated from 10 informants using in-depth and group interviews. Individual interview lasted 45 minutes to 1 hour for each informant, while 1 hour to 1hour 30 minutes was used for the group discussion. The groups were three consisting of four members each. In addition, archival sources such as lesson notes and class exercises were used to aid the selection of qualified candidate for the interviews and also to see how curriculum was adapted.” |
| **Data analysis methods** | “The qualitative data were analyzed using Interpretive Phenomenological Analysis (IPA).” |
| **Rigour** | N/A |
| **Stakeholder group(s)** | Teachers |
| **Recruitment** | “Participants (10 informants) for the qualitative phase of the study were selected [from the quantitative phase 387 participant teachers] based on the following criteria: a) The participants must have some experience of teaching children with LD in the primary school, b) The participant must have a child or children with LD in his or her class, c) participants must consent to be observed and interviewed, and d) have ample time to participate in the study.” |
| **Participant information** | **Total number**  10 |
|  | **Number per stakeholder group**  10 teachers |
|  | **Demographics**  N/A |
|  | **Other information**  N/A |
| **Relevance of DD and type** | Focus on inclusion for learning difficulties |
| **Setting information** | **School grade and type**  “general primary school teachers” |
|  | **Contextual information**  N/A |
| **Themes / topics** | “The qualitative phase of the study generated two categories of themes: meeting the learning needs of children with LD and schedules and instructional times” |
| **Extraction method for Results and Discussion** | Only qualitative results extracted for analysis. The part of the discussion focused exclusively on quantitative results is not extracted; the discussion of qualitative results and general conclusions are extracted for analysis. |
| **Quality** | Average |

### **Bannink et al. (2016)**

| **Full Reference** | Bannink, F., Idro, R., & Van Hove, G. (2016). Teachers’ and parents’ perspectives on inclusive education for children with spina bifida in Uganda. *Journal of Childhood & Developmental Disorders*, *2*(2), 2472-1786. |
| --- | --- |
| **Authors** | Bannink, Femke  Idro, Richard  van Hove, Geert |
| **Year** | 2016 |
| **Country/ies** | Uganda |
| **Aim** | “describe the primary school setting for children with spina bifida in the central region of Uganda and explore accessibility and inclusion with parents and teachers.” |
| **Objectives** | N/A |
| **Research question(s)** | N/A |
| **Methodology** | Mixed-methods |
| **Data collection methods** | “Qualitative semi structured interviews and school  observations were combined and conducted […] between June 2011 and December 2014.”  “The Index of Inclusion was used as a guide in the interviews with parents, students, and teachers. No full questionnaires were administered or scale scores calculated, as some of the items were not applicable in the Ugandan setting. Instead the overall themes of inclusive cultures, policies and practices were explored in semi-structured interviews appropriate for the school setting. Questions about relationships and respect between students and teachers were unmerged and rephrased to fit the cultural setting.” |
| **Data analysis methods** | “The semi structured interviews were transcribed, translated, coded and analysed using thematic analysis in NVivo version 10.” |
| **Rigour** | N/A |
| **Stakeholder group(s)** | Teachers, parents/caregivers, children with SEN |
| **Recruitment** | “The sample for this study was selected from a larger study population of 139 children with spina bifida in Uganda for which purposeful sampling was used in rehabilitation centres throughout the country to recruit families of children with spina bifida aged 4 to 14 years. For our study we selected all 63 children from the central region of Uganda from the database with 139 children. Of the 63 children, 41 children were schooling. […] The parents of the 41 children were approached for permission for a school visit, interview with the teacher, and classroom observation” |
| **Participant information** | **Total number**  90 |
|  | **Number per stakeholder group**  30 teachers  30 parents/caregivers  30 children with SEN |
|  | **Demographics**  “30 teachers (22 female, 8 male) of children with spina bifida”  “Children (18 male, 12 female) were observed in class, and parents/caregivers (27 mothers, 1 father, 1 foster parent, and 1 elder sister) were interviewed.” |
|  | **Other information**  “In total 16/30 children in school could walk without assistive devices; 6 children used crutches, and 8 used a wheelchair to ambulate. Fifteen had spina bifida, and 15 spina bifida and hydrocephalus. 24 practiced CIC, 1 used pampers, 5 were continent of urine.”  “The teachers were the child’s class teacher and interacted with the child on a daily basis. […] The class size varied between 28 and 69 children.” |
| **Relevance of DD and type** | Focus on inclusion of children with spina bifida in Uganda, where “66% of children with  spina bifida develop hydrocephalus”. “Fifteen had spina bifida, and 15 spina bifida and hydrocephalus.” |
| **Setting information** | **School grade and type**  Mainstream schools  “The class levels varied from lowest level nursery school (‘baby class’) to primary school level 5 (‘P5’).” |
|  | **Contextual information**  “The study population consisted of 63 parents of children with spina bifida; 30 teachers (22 female, 8 male) of children with spina bifida. The children’s average age was 5.97 (SD 2.20) years. In total, 41 children were schooling, and 22 were not. Of the schooling children, 26 were in nursery school, 11 in primary, and 4 in secondary school. […] The parent’s average age was 33.3 (SD 5.17) years. The majority (over 80%) of the families was Christian, 18% was Muslim.”  “Onlu 7 out of the 30 schools had classrooms which were accessible for wheelchair users and children using crutches using the Accessibility Standards. In two (2/30) of the schools cemented ramps were constructed, three (3/30) had installed wooden ramps, and two had a marram ramp (which was not functional in the rainy season). Parents paid  for the wooden ramps. None of the classrooms had space to manoeuver a wheelchair  in. The other school offices, libraries, and sports fields were not accessible according to the Accessibility Standards. In 5 out of the 30 schools children could practice continence management and had accessible toilets. […] None of the roads to school were accessible for children using assistive devices […] None of the children was in boarding school. The school with boarding sections had no accessible entrances to the dormitories, no accessible bathrooms, and insufficient space to manoeuver in.” |
| **Themes / topics** | School enrolment  Physical accessibility of school facilities  Classroom participation  Educational performance  Inclusive school policies |
| **Extraction method for Results and Discussion** | All extracted for analysis except for information on the study population. Presentations and discussions of quantitative findings ignored for analysis. |
| **Quality** | Good |

### **Bannink et al. (2020a)**

| **Full Reference** | Bannink F., Nalugya, R., Kawesa, E., Nambejja, H., Nizeyimana, P., Ojok, P., ... & Seeley, J. (2020a). ‘Obuntu Bulamu’–Development and Testing of an Indigenous Intervention for Disability Inclusion in Uganda. *Scandinavian Journal of Disability Research*, *22*(1), 403-416. <https://doi.org/10.16993/sjdr.697> |
| --- | --- |
| **Authors** | Bannink, Femke  Nalugya, Ruth  Kawesa, Elizabeth  Nambejja, Harriet  Nizeyimana, Pamela  Ojok, Patrick  van Hove, Geert  Seeley, Janet |
| **Year** | 2020 |
| **Country/ies** | Uganda |
| **Aim** | “In this article we describe the development and testing of an intervention based on the ‘Obuntu bulamu’ framework.” |
| **Objectives** | “give voice to unheard processes, forces, and families’ and children’s voices”  “The main objective of this study was to co-create a culturally appropriate intervention for disability inclusion based on the ‘Obuntu bulamu’ framework and test this intervention in 10 communities in Uganda.” |
| **Research question(s)** | N/A |
| **Methodology** | “we aimed to give voice to unheard processes, forces, and families’ and children’s voices using artistic and academic research processes” […]  “In the development and testing of the ‘Obuntu bulamu’ intervention, children and families used visual and oral narratives to describe their world and used these to challenge stereotypical views of disability in and outside Uganda.” […]  “The intervention was tested in 10 communities” [case studies] […]  “In this paper we describe the development and testing of the intervention using qualitative and ‘Afrocentric’ data collection to understand if the ‘Obuntu bulamu’ intervention has the potential to offer a culturally relevant alternative to imported disability inclusion interventions.” […]  “ Two ‘participatory action research meetings’ with all participants to co-create, test, and evaluate the ‘Obuntu bulamu’ intervention were held.” |
| **Data collection methods** | “Baseline, midline, and endline interview and focus group discussion data were collected from 64 parents and 33 teachers who participated in the testing of the intervention. Interview and focus group discussions focused on the perception and evaluation of the intervention. Observational data from the home and school setting, as well as visual narrative data, including drawings and photo narratives, were collected with the 32 children with different disabilities and 32 peers. Two ‘participatory action research meetings’ with all participants to co-create, test, and evaluate the ‘Obuntu bulamu’ intervention were held.” |
| **Data analysis methods** | “Data from the development and testing phases were reviewed following a thematic approach using framework analysis, a matrix-based system for organizing, reducing, and synthesizing data (Ritchie et al. 2013). A codebook was developed by three study team members and imported into NVivo 12. The thematically organized data were reviewed and synthesized. Meaningful themes and quotes were selected to highlight, explain or describe relevant themes.” |
| **Rigour** | “Rather than keeping a scientific distance, the research team immersed themselves culturally and socially with the children, parents, and teachers who were part of the project, something which has previously been described as Africentrism by Mkabela (2005) and Owusu and Mji (2013). For some team members this came naturally, others – especially the academics in the team – struggled with closing the gap in power relations between the ‘researcher’ and ‘the researched’ as they were often seen as more knowledgeable by participants and were sometimes expected to provide information on ‘how things should be done’. We attempted to minimize this distance by ensuring that most data collection was conducted by persons from the research team who participants felt closer to, in terms of educational and social background (including masters’ students with disabilities and parents of children with disabilities).” […]  “Identified themes were discussed and checked with the participants in follow up meetings.” |
| **Stakeholder group(s)** | Parents, teachers, children with SEN, peers without SEN |
| **Recruitment** | “We purposely selected children aged 8 to 14 years with different impairments, with different levels of household income and familial support, and who were enrolled in primary schools in the 10 communities with on average three children with disabilities per community. Participants were selected by the research team in collaboration with parents’ and district education office representatives.” |
| **Participant information** | **Total number**  161 |
|  | **Number per stakeholder group**  64 parents  33 teachers  32 children with SEN  32 peers |
|  | **Demographics**  N/A |
|  | **Other information** |
| **Relevance of DD and type** | 56.3% children with DD in study population, as reported by first author in private correspondence |
| **Setting information** | **School grade and type**  Mainstream primary schools |
|  | **Contextual information**  The intervention was tested in 10 communities in Wakiso district in Central Uganda. |
| **Themes / topics** | Developing, testing and evaluating the ‘Obuntu bulamu’ peer-to-peer support intervention |
| **Extraction method for Results and Discussion** | All Results section extracted for analysis. Initial part of Discussion which is focused on findings (rather than the research process, conceptual frameworks etc.) extracted for analysis. |
| **Quality** | Excellent |

### **Bannink et al. (2020b)**

| **Full Reference** | Bannink, F., Nalugya, R., & Van Hove, G. (2020b). ‘They Give Him a Chance’-Parents’ Perspectives on Disability and Inclusive Primary Education in Uganda. *International Journal of Disability, Development and Education*, *67*(4), 357-375. <https://doi.org/10.1080/1034912X.2019.1593326> |
| --- | --- |
| **Authors** | Bannink, Femke  Nalugya, Ruth  van Hove, Geert |
| **Year** | 2020 |
| **Country/ies** | Uganda |
| **Aim** | “In this paper, we question the usefulness of the application of globally assumed ‘international’ frameworks [for inclusive education guidelines] and look for a more indigenous explanation and framework, closer to the daily reality of persons with disabilities in Uganda.” |
| **Objectives** | N/A |
| **Research question(s)** | N/A |
| **Methodology** | Qualitative explorative study |
| **Data collection methods** | “In-depth interviews were conducted […]. In total two semi-structured interviews were conducted with each parent. After the initial semi-struc- tured interview, observations of the child and family were conducted at home, in school and the rehabilitation centre for each child. Based on the observations made questions for the second semi-structured interviews with parents were defined. Two interactive workshops were held with the children about their experiences in school and video case studies were developed together with the children.” […]  “This paper describes the parental perspectives collected in the case studies.” |
| **Data analysis methods** | “Qualitative data were organised using Nvivo. Data were analysed using the six thematic analysis phases of Braun and Clarke (Braun & Clarke, 2006): familiarising yourself with your data, generating initial codes, searching for themes, reviewing themes, defining and naming themes, and producing the report.” |
| **Rigour** | “Interviews were recorded and transcribed verbatim, translated in English, and back- translated by native Luganda speakers with English education.” […]  “The identified themes were verified with parent group leaders and community-based rehabilitation workers of disability-specific rehabilitation centres.” |
| **Stakeholder group(s)** | Parents of children with DD |
| **Recruitment** | “The eight children and their parents were purposively selected based on existing data about their family set-up, socio-economic status, school class, impairment, and impairment effects. All children in the cohort were grouped by the five areas above, and eight children were selected as ‘typical’ cases which would provide representative information about the cohort. The cases were pilot cases to inform a culturally appropriate, relevant, inclusive education intervention study starting from indigenous knowledge, education and support systems.” |
| **Participant information** | **Total number**  9 |
|  | **Number per stakeholder group**  9 parents |
|  | **Demographics**  “eight mothers and one father” |
|  | **Other information**  N/A |
| **Relevance of DD and type** | Cases were selected “rom a cohort of 78 children with neuro-disabilities” |
| **Setting information** | **School grade and type**  Mainstream primary schools  Grades 1-5 |
|  | **Contextual information**  N/A |
| **Themes / topics** | “The following themes were identified from the case studies and titled: ‘missing parts’, the ‘ability to manage’, ‘belonging and being recognised’, and ‘being given a chance’.” |
| **Extraction method for Results and Discussion** | All extracted for analysis, including additional section “Summary of Findings”. Sections of Results which are not relevant to education will not be considered for analysis. |
| **Quality** | Very Good |

### **Brydges & Mkandawire (2020)**

| **Full Reference** | Brydges, C., & Mkandawire, P. (2020). Perceptions and experiences of inclusive education among parents of children with disabilities in Lagos, Nigeria. *International Journal of Inclusive Education*, *24*(6), 645-659. <https://doi.org/10.1080/13603116.2018.1480669> |
| --- | --- |
| **Authors** | Brydges, Colton  Mkandawire, Paul |
| **Year** | 2020 |
| **Country/ies** | Nigeria |
| **Aim** | “This article examines the experiences of parents of CWD’s living in Lagos, Nigeria” |
| **Objectives** | N/A |
| **Research question(s)** | “First, we consider the perceptions of inclusive education among parents of children with disabilities, given their role as a key stakeholder, in a wider context marked by an acute shortage of resources and capacity to implement inclusive learning. Second, we seek to identify the lived experiences and socio-cultural conditions related to disability and parenthood that influence parents’ perceptions of the policy of inclusive education.” |
| **Methodology** | “A phenomenological design, involving semi-structured interviews to grasp common and divergent experiences, was used to engage with the lived experiences of parents of children with disabilities” |
| **Data collection methods** | “Interviews were conducted on the schools’ grounds, usually in a library or quiet room. Interviews were semi-structured in nature, relying on a common interview guide but leaving opportunity for elaborating on certain issues. The interviews focused on parents’ experiences accessing the education system, societal and insti- tutional obstacles they faced, and their impressions of inclusive and special schools. The discussions were conducted in English, and all interviews were audio-recorded using a tablet computer.” |
| **Data analysis methods** | “Data were analyzed through a process of thematic content analysis. Interviews were manually transcribed, and the transcripts were reviewed to identify recurring responses and common themes. Recurring responses and themes were tallied, allowing the authors to discuss and refine the coding of the data. Subsequently four central themes were identified, which serve to organise the research findings.” |
| **Rigour** | N/A |
| **Stakeholder group(s)** | Parents of children with SEN |
| **Recruitment** | “Purposive sampling was initially used to identify participants, and subsequently a snowball method was used to further expand the sample, as school administrators would refer the primary researcher to other institutions that were serving CWD’s. Prior connections with local stakeholders were also invaluable in gaining access to otherwise hidden populations.” |
| **Participant information** | **Total number**  12 |
|  | **Number per stakeholder group**  12 parents |
|  | **Demographics**  10 female |
|  | **Other information**  “The socio-economic status of the interviewees varied widely: some parents were unemployed, while others had formal employment in government or the private sector.” |
| **Relevance of DD and type** | Around 50% participants had a child with DD, as reported by first author in private correspondence |
| **Setting information** | **School grade and type**  “These parents’ children were in a variety of educational settings, ranging from public to private and inclusive to special schools.”  Primary education |
|  | **Contextual information**  “The primary research for this project was conducted in the city of Lagos, Nigeria in July, 2013 […] in a number of areas of Lagos, including Victoria Island and Yaba, with the majority taking place in Surulere, a local government jurisdiction located in metropolitan Lagos with relatively low population density and predominantly middle class population” |
| **Themes / topics** | “perceptions of inclusive and special schools, community perceptions of disability, gendered experiences, and the hierarchy of impairment.” |
| **Extraction method for Results and Discussion** | All extracted for analysis. Sections of Results which are not relevant to education will not be considered for analysis. |
| **Quality** | Good |

### **de Jager (2011)**

| **Full Reference** | de Jager, P. S. (2011). The identification of sensory processing difficulties of learners experiencing Asperger's Syndrome (AS) in two mainstream Grade R classes. *South African Journal of Childhood Education*, *1*(2), 16. |
| --- | --- |
| **Authors** | de Jager, Petronella |
| **Year** | 2011 |
| **Country/ies** | South Africa |
| **Aim** | “The purpose of this study is to explore the sensory processing difficulties of Grade R learners that are diagnosed with Asperger’s Syndrome (AS) in two schools in South Africa.” |
| **Objectives** | N/A |
| **Research question(s)** | “What are the sensory processing difficulties experienced by AS learners within a mainstream Grade R class?” |
| **Methodology** | “This research approach was purely qualitative” […]  “We attempted to map out, or explain more fully, the richness and complexity of human behaviour of learners with AS, by studying it from more than one standpoint. Henning (2007, p. 147) argues that if the outcome of the interview survey corresponded with the observation study of the same phenomenon, the researcher would be more confident of the findings. By using observations and interviews as our data instruments, we hoped that the two sets of data would help to ensure consistency, reliability and validity.” |
| **Data collection methods** | “The researchers employed interviews and observations in this study.” […]  “Individual interviews were scheduled with the two educators of the AS learners […] we scheduled an interview with a psychologist who works with learners that live with AS. These semi- structured, ‘face-to-face’ interviews were conducted […] The interviews with the educators were scheduled and conducted in the medium of English, as that was the language of instruction in their classrooms. Using open-ended questions, the respondents were asked to give their own answers to the questions. Verbal, probing questions were provided when necessary. These probing questions were intended to help the respondents think more deeply about the issue at hand, and helped guide them to stay focussed on the research topic (Hammell, Carpenter & Dyck, 2005, p. 32). The interview with the psychologist focussed on how she, in a school setting, works with teachers and assists in mediating sensory processing skills. […] Data from the observations were gathered through making continuous notes on a pre-planned observation schedule. Coding procedures, involved categories, labelling and naming of various aspects around the topic of sensory factors were used. […] Since both these learners experience AS, and since their performance fluctuated from day-to-day and minute-to-minute, we set aside the whole month of September 2008 to observe their sensory processing development.” |
| **Data analysis methods** | “During our analysis of the data, we transcribed all the interviews and looked for common themes that arose, and proceeded to compare them to observational data recorded. The information gathered were categorised and interpreted manually.” |
| **Rigour** | “To obtain corroboration and confirmation of our findings we received written consent letters from both parents, as these learners were too young to give consent themselves.” |
| **Stakeholder group(s)** | Learners with DD, teachers, psychologist |
| **Recruitment** | “We intentionally selected two individuals likely to provide is with a greater understanding of the concept under scrutiny. We located two learners, aged between five and seven years of age, who had been clinically diagnosed with AS. They were attending a Grade R class in and around the Cape Peninsula.” |
| **Participant information** | **Total number**  2 cases, 3 informants |
|  | **Number per stakeholder group**  2 learners with DD (cases), 2 teachers, 1psychologist |
|  | **Demographics**  “aged between five and seven years of age”, male (cases) |
|  | **Other information**  N/A |
| **Relevance of DD and type** | Case study of two children with ASD |
| **Setting information** | **School grade and type**  Grade R |
|  | **Contextual information**  N/A |
| **Themes / topics** | “The two learners were observed:  • Across settings (physical, cultural, social, and group and task);  • The way they used their senses of tactile, auditory, visual, gustatory, olfactory, vestibular and proprioception in their responses; and  • How they interacted and responded (interaction and communication).” |
| **Extraction method for Results and Discussion** | All extracted for analysis |
| **Quality** | Average |

### **de Jager and Condy (2017)**

| **Full Reference** | De Jager, P., & Condy, J. (2017). The influence of executive function challenges on the behavioural adaptation of one learner with autism spectrum disorder. *South African Journal of Childhood Education*, *7*(1), 1-11. <https://doi.org/10520/EJC-7e57ebea3> |
| --- | --- |
| **Authors** | de Jager, Petronella  Condy, Janet |
| **Year** | 2017 |
| **Country/ies** | South Africa |
| **Aim** | “This study explores the influence of executive function (EF) challenges experienced by one learner with ASD on his behavioural adaptation in an inclusive school environment.” |
| **Objectives** | N/A |
| **Research question(s)** | N/A |
| **Methodology** | “This study is grounded in a qualitative case study conducted within an interpretative research paradigm. Yin (2003:18) defines a case study research as comprising an all-encompassing method covering the logic design, data collection techniques and specific approaches to data analysis. It is a preferred strategy when 'how' and 'why' questions are posed, the researcher has little or no control over events and the focus is on a contemporary phenomenon within some real-life context. This inquiry was followed to help understand one unique ASD learner and explain the complexity of the behavioural adaptation challenges that he was experiencing in his classroom. An interpretive research approach is 'an approach that focusses on examining how individuals make meaning of their life experiences' (Pietkiewiz & Smith 2014:7). It is informed by the idea that everyone is unique and should be studied as such. This approach involves a combination of psychological, interpretative and ideological components (Gill 2014). Together these afford an in-depth understanding that incorporates attention to the participant's beliefs and subjective experiences, feelings and values (Nichols 2011).” |
| **Data collection methods** | “By employing observations and an interview for data collection, the researcher was able to study Learner T's behaviour from more than one standpoint (Henning Van Rensburg & Smit 2007). The focus was on his behaviour, but the researcher tried to interpret the reasons behind it - to establish the influences of EF challenges experienced by an ASD learner on his behavioural adaptation in an inclusive school environment. As the number of occurrences of certain phenomenon mounted, using both observations and an interview as data collection instruments enabled the researcher to triangulate the information. It was hoped that this would heighten the dependability and trustworthiness of the interpretation of the data collected (Zohrabi 2013). The researcher put aside five school days (between 08:30 and 14:30) for continuous observations […] During the observation period, minute-to-minute examples of behavioural adaptation challenges experienced by Learner T were noted on a preplanned observation schedule. The responses of Teacher SM to these behavioural challenges were included and correlated. […] By not only gathering information but also observing Learner T's interacting in his environment the researcher could experience the unique way in which the learner experienced life situations differently from his peers.” […]  “After the five observations, an interview was arranged with Teacher SM. The interview was tape-recorded to ensure descriptive validity (Isaac 2015). […] The interview questions were fit for purpose, designed with the research question in mind. The semi-structured interview was conducted after-hours in Teacher SM's classroom. This style of interviewing allowed for more flexibility and created space for the researcher to pursue lines of inquiry stimulated by the interview (Rule & John 2011). As Teacher SM was not formally trained in teaching ASD learners, probing questions were added where necessary. These probing questions assisted in helping her respond more fully and contributed more relevant information to the research results.” |
| **Data analysis methods** | “The researcher used standard procedures to organise, date and code the data from the observations and the one interview. This assisted her in establishing links between the various data collected (Henning et al. 2007). Once she had identified the themes within each section of the transcript, she looked for possible connections between them. The main theme comprised the identified 'EF challenges'. These five challenges included response inhibition, emotional control, task initiation, goal-directed persistence and sustained attention, and flexibility of thinking. The subtheme which was the 'behavioural adaptation challenges' juxtaposed all five themes. The next stage involved a more analytical or ordering process, as the researcher made sense of the connection between the main theme and subthemes as they emerged. The main five themes were categorised using grouping and colour-coding procedures. Every EF challenge was given a specific colour code, which included 'green' for response inhibition, 'blue' for emotional control, 'yellow' for task initiation, 'red' for goal-directed persistence and sustained attention, and 'purple' for flexibility of thinking challenges. The 'behavioural adaptation challenges' were individually identified and linked according to these EF challenges and colour-coded accordingly.” |
| **Rigour** | “So as not to fall prey to bias, the researcher made notes regarding the environment and external elements that may have had an impact on the research findings. Continuous writing incidents while observing Learner T were done while occurrences took place. As the themes emerged, the researcher linked the support-related data of the interview with Teacher SM and related research with the observation data. All these standard procedures helped to ensure that the data collected rendered a 'thick description' of all the occurrences taking place (Henning et al. 2007:85).” |
| **Stakeholder group(s)** | Learner with DD and teacher |
| **Recruitment** | “Finding a learner who can fulfil all the requirements needed to qualify for this study was difficult. The researcher found that the learners she identified, the schools and parents were unwilling to be involved in the study. Therefore, this learner was purposively 'hand-picked' on the basis that he was clinically diagnosed with ASD by a registered educational psychologist. At the time of data collection, he was a 9-year-old learner in Grade 3 in a private school in the Cape Peninsula.” |
| **Participant information** | **Total number**  2 |
|  | **Number per stakeholder group**  1 learner (case), 1 teacher (informant) |
|  | **Demographics**  9 years old, male (case) |
|  | **Other information**  N/A |
| **Relevance of DD and type** | Case study of a child with ASD |
| **Setting information** | **School grade and type**  Grade 3 |
|  | **Contextual information**  N/A |
| **Themes / topics** | Response inhibition challenge  Emotional control challenge  Task initiation challenge  Goal-directed persistence and sustained attention challenges  Flexibility of thinking challenge |
| **Extraction method for Results and Discussion** | All extracted for analysis |
| **Quality** | Good |

### **Engelbrecht et al. (2001)**

| **Full Reference** | Engelbrecht, P., Swart, E., & Eloff, I. (2001). Stress and coping skills of teachers with a learner with Down's syndrome in inclusive classrooms. *South African journal of education*, *21*(4), 256-259. |
| --- | --- |
| **Authors** | Engelbrecht, Petra  Swart, Estelle  Eloff, Irma |
| **Year** | 2001 |
| **Country/ies** | South Africa |
| **Aim** | “identify stressors for teachers in an inclusive educational approach and coping skills employed to ameliorate the negative effect of these stressors.” |
| **Objectives** | “The purpose of the present study was to  • Investigate the stressors teachers attributed to coping with a  learner with Down’s Syndrome in their mainstream classroom;  • Identify the range of coping strategies these teachers employ to  ameliorate stress during inclusion.” |
| **Research question(s)** | N/A |
| **Methodology** | “For the present study a qualitative methodology was chosen to gain a more in-depth under- standing of teachers' perception of the stressors and the coping strategies employed within an inclusive classroom.” |
| **Data collection methods** | “Teachers were interviewed after they completed the questionnaire. The interviews were more structured as the completed questionnaire formed the basis of the discussion.” […]  “The following sources of data were relevant in this study: a ques- tionnaire as well as richly descriptive field notes and transcripts based on interviews with members of the sample after they had completed the questionnaire. The questionnaire in question was a localised ver- sion of the Teacher Stress and Coping (TSC) questionnaire (Forlin, 1998).” […]  “The questionnaire and personal interviews focused on the participants' under- standing of, experience with and attitudes towards the inclusion of learners with Down’s syndrome.” |
| **Data analysis methods** | “Data were analysed qualitatively. The process involved the simul- taneous coding of raw data from the questionnaire, interviews and field notes, the construction of categories that capture the relevant characteristics of the data's content as well as the further clustering of categories into broad themes (Bos & Tarnai, 1999; Merriam, 1998).” |
| **Rigour** | “In order to enhance validity and reliability the following strategies were implemented:  • Peer examination: colleagues were consistently asked to comment on the findings as they emerged, in order to clarify possible inconsistencies.  • Collaborative mode of research: the research was conducted byna research team of three researchers who were involved in all the phases of the research from conceptualizing the study to writing up the research.  • Clarification of the three researchers' assumptions, worldview and theoretical orientation at the outset of the study (Merriam, 1998).” […]  “In order to enhance the generalisability of the research results the research was conducted in both the Gauteng and Western Cape provinces of South Africa to maximise diversity and to allow the results to be applied to a greater range of other situations.” |
| **Stakeholder group(s)** | Teachers |
| **Recruitment** | “In the focus on Down’s syndrome five teachers in the Western Cape province and five teachers in the Gauteng province of South Africa who each have a learner with Down’s syndrome in their mainstream classes were identified. Permission from either the provincial education departments or local school governing bodies was sought to interview the teachers after they have completed the questionnaire in the presence of a researcher. The sample therefore can be described as non-random, purposeful and small (Merriam, 1998).” |
| **Participant information** | **Total number**  10 |
|  | **Number per stakeholder group**  10 teachers |
|  | **Demographics**  “The 10 teachers were all female with ages ranging from 26 to 56 plus.” |
|  | **Other information**  “each have a learner with Down’s syndrome in their mainstream classes” […]  “They had between 12 to 38 years of teaching experience but for seven of them it was a first experience with a learner with Down’s syndrome in an inclusive classroom. Only three of the teachers had any formal training in dealing with learners with special needs. They had completed a Further Diploma in Remedial Teaching at an earlier stage.” |
| **Relevance of DD and type** | Focus on Down’s syndrome (ID) |
| **Setting information** | **School grade and type**  mainstream primary schools  “The schools involved in this project are former Model C mainstream schools and although agreeable to accommodate learners with special needs according to Section 5(1) of the S A Schools Act 84 of 1996 (Republic of South Africa, 1996) all have the condition that education within a main- stream classroom can only be continued if learners cope (as under- scored by section 12(4) of the Schools Act).” […]  “Of the learners with Down’s syndrome in the 10 inclusive classrooms five were male and five female. Four of them were in Grade 1, of whom three were eight years old and one nine years of age. The two learners in Grade 2 were both 11 years old. The one learner in Grade 3 was 13 years of age and the three learners in Grade 4 also 13 years old.” |
|  | **Contextual information**  “five teachers in the Western Cape province and five teachers in the Gauteng province of South Africa” […]  “In addition to the learner with Down’s syndrome the teachers had to deal with other learners with special needs in their classes of which 24 experienced learning difficulties, five had a speech impairment, three were vision impaired, two hearing impaired, one had a physical impairment and one an intellectual impairment. Three of the teachers indicated that they had to deal with learners at risk (mostly from broken homes) in their classes, as well.” |
| **Themes / topics** | “On the basis of the TSC questionnaire the following cate- gories were identified:  Administrative issues  Support  Health, safety and hygiene issues  Learner behaviour  Issues related to the classroom  Parents of learners with Down’s syndrome  Professional competence  Personal competence  A few broad themes emerged from a further process of clustering.” […]  “teachers' perceived self-competence” […]  “every learner with Down’s syndrome is an individual in his or her own right” […]  “Meeting the needs of the other learners in the class” […]  “The following three broad themes of coping strategies emerged that teachers chose to employ as resources to combat stress within an inclusive educational approach:  • Problem-focused strategies would include the following:  -Make a plan of action and follow it  -Come up with different solutions to difficult issues  -Concentrate on what has to be done next  • Collaborative strategies would include the following:  -Enlist the help of other learners in the class  -Discuss the situation with your colleagues  -Seek help and resources from other teachers  -Discuss the situation with the learner's parents  -Discuss the situation with your principal  • Emotion-focused strategies would include the following:  -Maintain a sense of humour  -Try to look on the bright side of things S Seek spiritual and religious support” |
| **Extraction method for Results and Discussion** | All extracted for analysis except for initial paragraph on questionnaire results |
| **Quality** | Very Good |

### **Engelbrecht et al. (2003)**

| **Full Reference** | Engelbrecht, P., Oswald, M., Swart, E., & Eloff, I. (2003). Including learners with intellectual disabilities: Stressful for teachers?. *International Journal of Disability, Development and Education*, *50*(3), 293-308. <https://doi.org/10.1080/1034912032000120462> |
| --- | --- |
| **Authors** | Engelbrecht, Petra  Oswald, Marietjie  Swart, Estelle  Eloff, Irma |
| **Year** | 2003 |
| **Country/ies** | South Africa |
| **Aim** | “The purpose of this research was to focus on the stressors experienced by teachers related to the specific situation of including learners with intellectual disabilities in South African mainstream classes and to investigate the relationship between stress and six independent variables, namely age, gender, total number of years teaching, highest qualification held, number of learners in the class, and numbers of years involved with inclusive education.” |
| **Objectives** | N/A |
| **Research question(s)** | N/A |
| **Methodology** | “In this study a combination of quantitative and qualitative methods was employed. The study was conducted in two phases.” [1 quantitative, 2 qualitative] […]  “The qualitative phase of the study was deemed important to gain a more in-depth understanding of teachers’ perceptions of the stressors within an inclusive classroom.” |
| **Data collection methods** | “During phase two, individual interviews were conducted with 10 of the 55 teachers who participated in phase one of the study. […] The interviews were structured with the completed questionnaire forming the basis of the discussion.” […]  “The Teacher Stress and Coping Questionnaire (TSC), as developed by Forlin (1998), relates specifically to stress and coping in inclusive education.” |
| **Data analysis methods** | “The questionnaire as interview guide constituted a descriptive analytical framework for the analysis of the qualitative data (Patton, 1990). The transcribed data of the 10 interviews were carefully examined and compared within the framework of the TSC.” |
| **Rigour** | N/A |
| **Stakeholder group(s)** | Teachers |
| **Recruitment** | “Teachers from two provinces (Gauteng and Western Cape) in South Africa participated in this study. The respective education departments were contacted for data on teachers who were currently including learners with intellectual disabilities in a mainstream classroom. As the implementation of inclusive education is only now in the final process of legislation, it was difficult to identify an adequate number of participants for this research study. Care was, however, taken to purposefully select teachers who were (a) including a learner with a mild to moderate intellectual disability in their mainstream classes, and (b) from a range of schools representative of the whole spectrum of schools in South Africa (i.e., from highly resourced schools to schools from traditionally disadvantaged communities).” […]  “The principals of the selected schools were contacted in order to establish contact with the teacher. Appointments were made to provide a synopsis of the research project, the questionnaire involved, and to deliver the questionnaire to the various schools. The participants were assured of anonymity. Collection dates for the completed questionnaires were mutually agreed upon.” […]  “The ten participating teachers (five each from Gauteng and Western Cape provinces) were purposefully selected as “information-rich cases” and as representative of the whole spectrum of schools in South Africa. When approached to participate in the second phase of this research study they indicated their willingness.” |
| **Participant information** | **Total number**  10 |
|  | **Number per stakeholder group**  10 teachers |
|  | **Demographics**  “Ages ranged from 26 to more than 55 years, with the majority between the ages of 26 and 45 years. The participants included 52 women and three men” |
|  | **Other information**  “Their teaching experience ranged from one to 36 years. Approximately 67% of the participating teachers were highly experienced and had been teaching for at least 10 years. The language of instruction used by the teachers was English or Afrikaans. The number of years teaching experience with learners with any disability in their mainstream classes ranged between zero and 36 years. Fifteen of the participants indicated that they had between zero and four years experience with learners with disabilities in their mainstream classes. In terms of qualifications, most (41) of the participants had completed a teacher’s diploma, six had completed a bachelor’s degree along with a teacher’s diploma, one had completed a bachelor’s degree, and one had completed a master’s degree.” |
| **Relevance of DD and type** | Focus on inclusion for intellectual disability  “55 learners with intellectual disabilities included” in the school  “The categorisation of learners as having an intellectual disability was based on their low level of cognitive functioning as previously determined by psychometric evaluation (where still in use) or on grounds of repeated failure at school. The learners’ intellectual disabilities could be classified as mild to moderate. The majority of learners had to repeat grades presenting a further complicated scenario of over-aged learners.” |
| **Setting information** | **School grade and type**  “An analysis of the biographical data revealed that teachers in the study taught across all grades.” |
|  | **Contextual information**  “The number of learners in the various classes ranged from 24 to 72. The number of learners in the schools ranged between 300 and 1,500. The ages of the 55 learners with intellectual disabilities included by the teachers were between 6 and 23 years. Thirty-four of this group were male and 21 were female.” |
| **Themes / topics** | Most Stressful Issues for Teachers During Inclusion  Least Stressful Issues for Teachers During Inclusion |
| **Extraction method for Results and Discussion** | All extracted for analysis except the findings on “Relationship Between Stress and Independent Variables” (all quantitative). Other presentations and discussions of quantitative findings ignored for analysis. |
| **Quality** | Good |

### **Lopes et al. (2009)**

| **Full Reference** | Lopes, T., Eloff, I., Howie, S., & Maree, J. G. (2009). South African teachers' experiences of children in their classrooms who may have ADHD. *Journal of Psychology in Africa*, *19*(3), 347-354. <https://doi.org/10.1080/14330237.2009.10820301> |
| --- | --- |
| **Authors** | Lopes, Tina  Eloff, Irma  Howie, Sarah  Maree, Jacobus G. |
| **Year** | 2009 |
| **Country/ies** | South Africa |
| **Aim** | “The study sought to gain some understanding of South African teachers’ experiences of children in their classrooms who might have ADHD.” |
| **Objectives** | N/A |
| **Research question(s)** | “The main research question was: How do teachers experience children in their classrooms who may have ADHD? […] The main research question was subdivided into the following sub-questions.  1. How do teachers manage their classrooms with children in their classrooms who may have ADHD?  2. How do teachers experience the teaching and learning of children who may have ADHD?” |
| **Methodology** | “The study used a narrative research design. In such a de- sign, researchers describe the lives of persons, tell stories about people’s lives and write narratives on people’s experiences (Creswell, 2002; Lopes, 2008). The focus of a narrative research design is the collection of data, the description of people’s personal stories and the discussion of the meaning of people’s experiences. Personal accounts were used as a narrative research method in the study (Clandinin & Connelly, 2000; Creswell, 2002). […] A narrative research design was appropriate in this study as it vividly illustrated the teachers’ experiences (and the meanings they attached to their experiences). The narrative, as an easy and accessible format for readers, indicates what educational experiences of teachers should be addressed to ensure academic success.” |
| **Data collection methods** | “Interviews were conducted with 17 teachers in this in-depth, face-to-face qualitative study. The interviews were semi-structured so that the teachers could create and share their own stories of classroom experiences. The interviews were guided by an interview schedule […] based on the research questions (Lopes, 2008).” […]  “The semi-structured interviews allowed the teachers to tell their own stories of their classroom experiences (Graham, 2000). The interviews were not restricted to the research questions – the teachers were encouraged to share all their stories of their classroom experiences. […] In the interviews, the researcher could explore the classroom behaviour of the children who may have had ADHD by probing the teachers for more in-depth experiences and stories. The interviews enabled the researcher to uncover the meaning the teachers attached to their experiences in their classrooms (Kvale, 1996).” […]  “A lot of time was spent with the teachers during the interviews, which facilitated rapport and helped put the teachers at ease. The primary re- searcher, a highly trained and experienced academic, not only in conducting qualitative research but also in implementing anti-bias programmes, conducted the interviews and tried to eliminate bias.” |
| **Data analysis methods** | “Themes were elicited from the textual data; narratives were developed from the themes.” […]  “The textual data underwent classical content analysis (Krippendorf, 1980; Weber, 1990) to facilitate the researcher’s understanding of the text, action and/or narrative through the interpretation of emerging themes (Tesch, 1990).” |
| **Rigour** | “In order to ensure the trustworthiness of the analysed data, certain checks were put in place to verify the data and the analysis (Fade, 2003; Guba in Schurink, Schurink and Poggenpoel, 1998; Pope, Ziebland, & Mays, 2000). The data had to meet the following criteria: (1) credibility, (2) transferability, (3) depend- ability and (4) confirmability (Seale, 1999). […] Trustworthiness was further enhanced by peer examination (the supervisors reviewed the work at all stages), independent coding and comparison of information at different stages of the research.”  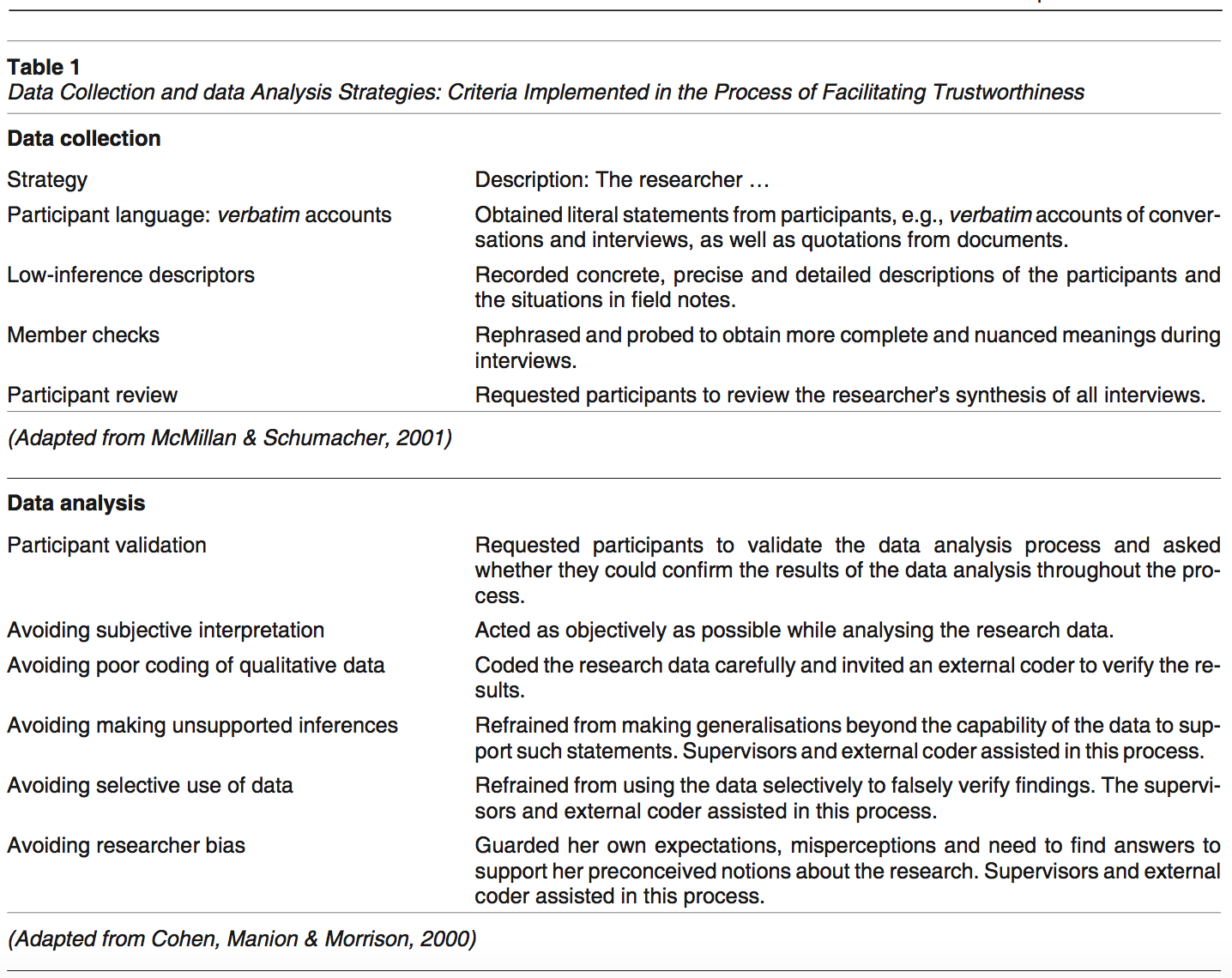 |
| **Stakeholder group(s)** | Teachers |
| **Recruitment** | “Purposive sampling was considered appropriate for the study as the primary researcher and her supervisors had ascertained, during prior site visits, that the particular schools had children with ADHD in their classrooms. Furthermore, the participating schools were situated in different contexts/environments (Gay & Airasian, 2003, Silverman, 2000; Onwuegbuzie & Leech, 2007). […] Convenience sampling was carried out because the primary researcher had previously worked with the schools and teachers on a University of Pretoria project and consequently knew the teachers.” |
| **Participant information** | **Total number**  17 |
|  | **Number per stakeholder group**  17 teachers |
|  | **Demographics**  “All 17 of the teachers/participants in the study were women: 10 were African, four were white, two were Indian and one was coloured.” |
|  | **Other information**  N/A |
| **Relevance of DD and type** | Focus on children “who may have ADHD”  “The expression ‘children who may have ADHD’ was  adopted for the purpose of the study because not all the children had undergone formal diagnostic psychological procedures. Because it is fairly safe to assume that, due to the high prevalence rates of ADHD in children generally, children with ADHD would most probably be present in the classrooms, we opted for a more tentative descriptor.” |
| **Setting information** | **School grade and type**  N/A |
|  | **Contextual information**  “Three schools (17 teachers) in the Pretoria area in Gauteng Province in South Africa participated in the study. One school was situated in suburban Pretoria. Here, the children differed in background and language – some children had emigrated from other countries in Africa and therefore had French, Portuguese and other African languages as their first language. The staff complement was racially mixed.  The second school was situated in an informal settlement where the children and teachers were all Africans at the time of the study. The surrounding community was poor as most of the parents were unemployed and could not pay the school fees. The school grows vegetables to provide meals for some of the children. It has sufficient tables, chairs and textbooks for the children, but there are not enough computers for all the children in a classroom. One teacher said there were also not enough educational products and learning support materials, such as educational toys and books, for the children.  The third school was situated on the western side of the Pretoria municipality. Before 1994, it consisted predominantly of Indian children and teachers, but today it has children and teachers from diverse racial backgrounds.” |
| **Themes / topics** | “Five themes emerged from the data (Table 2). However, for the purpose of this article, only the first three will be discussed here. They are (1) “*Keep them busy*”, (2) *“It does challenge you – we’re not perfect,”* and (3) “*It needs sharing as teachers and help from a specialist.*” “ |
| **Extraction method for Results and Discussion** | All extracted for analysis |
| **Quality** | Excellent |

### **Majoko (2016)**

| **Full Reference** | Majoko, T. (2016). Inclusion of children with autism spectrum disorders: Listening and hearing to voices from the grassroots. *Journal of autism and developmental disorders*, *46*(4), 1429-1440. <https://doi.org/10.1007/s10803-015-2685-1> |
| --- | --- |
| **Authors** | Majoko, Tawanda |
| **Year** | 2016 |
| **Country/ies** | Zimbabwe |
| **Aim** | “The current study investigated social barriers and enablers of inclusion of children with ASD in mainstream primary school classrooms in Zimbabwe according to the perspectives and experiences of regular teachers.” |
| **Objectives** | N/A |
| **Research question(s)** | “Specifically, the current study addressed the following research questions:  1.What are the social barriers to inclusion of children with ASD in Zimbabwean mainstream primary school classrooms?  2.What are the enablers of inclusion of children with ASD in mainstream primary school classrooms in Zimbabwe?” |
| **Methodology** | “In order to establish the social barriers and enablers of inclusion of children with ASD in mainstream primary school classrooms, the current study used a qualitative methodology. Qualitative methodologies are used when information about a researched phenomenon is limited (Silverman 2009) and when the purpose of the study is to describe experiences through identification of themes and developing theories based on the participant’s perception of events (Corbetta 2003; Lewis 2003).” |
| **Data collection methods** | “According to Pierce (2008), conducting interviews with participants is among the methods of collecting data in qualitative research. Thus, the present study used semi-structured interviews with open ended questions to provide a framework for the interview, but encouraged participating regular teachers to describe social barriers and enablers of inclusion of children with ASD in mainstream classrooms.” […]  “The researcher conducted individual in-depth interviews between January 2013 and October 2014, each lasting an average of 41 min. Prior to the beginning of the interview, informed consent was obtained from the participant. The interviews followed an in-depth semi-structured format that explored regular teachers’ perspectives and experiences regarding the inclusion of children with ASD in mainstream primary school classrooms. Participating regular teachers were asked about their experience of working with children with ASD within mainstream primary school classes and their advice about establishing and reinforcing an inclusive environment for these children.” |
| **Data analysis methods** | “Individual in-depth interviews were transcribed verbatim. The transcriptions were read numerous times and coded. Cross-case analysis was subsequently performed for each individual in-depth interview (Lewis 2003) and pattern coding was followed. In pattern coding, interview transcripts were reviewed and themes emerging from each interview cross checked while identifying emerging patterns or themes. During the coding process, negative or contrasting cases and frequencies of themes were identified. Emerging themes were subsequently organised around the aforementioned research questions.” |
| **Rigour** | “Member checking of data collected was conducted so as to establish rigour and trustworthiness in the findings (Pierce 2008). This involved taking interview transcripts to participating individual regular teachers who read them to validate their contents and the meanings generated from them. In the current study, data analysis was a reciprocal process between the researcher and an external reader who had expertise in qualitative research. Analysis documents were presented to the external reader to examine their credibility.” |
| **Stakeholder group(s)** | Teachers |
| **Recruitment** | “A sample of 21 regular teachers purposively drawn from mainstream primary schools participated in the study. Inclusion criteria were established so as to generate a pool of regular teachers who were relatively homogeneous and who were hypothesized to have experiences relevant to the research questions. Regular teachers needed to meet the following inclusion criteria in order to participate in the current study:(1) had at least a 3 year mainstream primary school teachers’ diploma; (2) had at least 3 years of teaching experience in mainstream primary school classroom with children with ASD; and (3) teaching in a mainstream primary school classroom with children with ASD in a public primary school in Harare educational province of Zimbabwe. The adequacy of the sample of regular teachers was determined when theoretical saturation was reached” |
| **Participant information** | **Total number**  21 |
|  | **Number per stakeholder group**  21 teachers |
|  | **Demographics**  “9 males and 12 females”; “aged between 27 and 65 years” |
|  | **Other information**  “Their teaching experience ranged from 4 to 20 years. Eight of the participants had additional qualifications, post-graduate degrees in education.” |
| **Relevance of DD and type** | Focus on inclusion for ASD |
| **Setting information** | **School grade and type**  “wide range of primary school level classes”  “regular primary school” |
|  | **Contextual information**  N/A |
| **Themes / topics** | Social barriers to inclusion:  ‘Self-imposed’ social isolation, Behaviour, Bullying, Rule-bound, Transition, Social rejection  Enablers of inclusion:  Teacher professional preparation and development, Academic/ social development, Teaching and learning strategies, Structure, ASD awareness, Advocacy |
| **Extraction method for Results and Discussion** | All extracted for analysis |
| **Quality** | Average |

### **Majoko (2017)**

| **Full Reference** | Majoko, T. (2017). Practices that support the inclusion of children with autism spectrum disorder in mainstream early childhood education in Zimbabwe. *SAGE Open*, *7*(3), 2158244017730387. [https://doi.org/10.1177/2158244017730387](https://doi.org/10.1177%2F2158244017730387) |
| --- | --- |
| **Authors** | Majoko, Tawanda |
| **Year** | 2017 |
| **Country/ies** | Zimbabwe |
| **Aim** | “This study explored support practices for including learners with autism in Zimbabwean regular ECD classes so as to add to the limited national literature base as well as to glean practices to optimize access, equity, equality, and participation of these learners in education in tandem with the global and national priority.” |
| **Objectives** | N/A |
| **Research question(s)** | “Specifically, the subsequent research question was addressed:  Research Question 1: What are teachers’ support practices for including learners with autism in regular ECD classes in Midlands educational provinces of Zimbabwe?” |
| **Methodology** | “To solicit teachers’ support practices for including learners with autism in regular ECD classes in Midlands educational province in Zimbabwe, this study was embedded in a multiple-case study design. Qualitative research methodology interrogates a given phenomenon from the people experiencing it through textual descriptions that can be analyzed for themes and can induce transferable interpretations (Pierce, 2008; Silverman, 2009; Wiersma & Jurs, 2009). As the study focused on individual participants’ views, experiences, and practices, its methodological approach was entrenched in phenomenology. Phenomenological research is grounded in understanding of daily life situations of individuals (Pierce, 2008). Teachers’ practices for supporting inclusion of learners with autism in mainstream ECD classes constituted components of daily life that were the focus of attention of the researcher.” |
| **Data collection methods** | “Each participating school constituted a unit and unveiled a distinct context of regular ECD setting and culture. To interrogate support practices for including learners with autism in regular ECD classes, similarities and differences from these pedagogical settings were discerned by the researcher. The researchers carried out 18 individual interviews with participants, one interview per participant. Each interview lasted an average of 44 min. […]Audiotaping facilitated more accurate collection of data. It also enabled the researchers’ attention to the participants while interviewing them. English was used in carrying out individual interviews. Participants were interviewed in their classrooms after school hours, and participation was voluntary without any compensation. In spite of the organization and structure during interviewing because of the utilization of the interview guide, the researcher ensured flexibility through context-specific interrogation of issues. The researcher carried out 18 nonparticipant class observations, one per participant. […] Nonparticipant observations were grounded in gleaning teachers’ support practices for including learners with autism in regular ECD classes. These enabled the solicitation of “thick descriptions” for understanding respective teachers’ support practices for including learners with autism in regular classes. These observations lasted 40 min on average. The researcher recorded field notes using an observation guide. The researcher held informal conversations with the participants regarding pedagogy after each observation as a follow-up to ensure clarity.” |
| **Data analysis methods** | “Data that were collected from several sites, methods, and sources were triangulated to illuminate on emerging themes (Creswell, 2009). Interviews, observations, and analyses of documents assisted in assessment of the extent of convergence and complementarity of findings and elaboration on divergences between findings yielded (Silverman, 2009). […] Triangulation of data constituted: identification of the study focus; discerning trends within and across sets of data; generation of initial codes; searching for similarities and differences for identification of initial themes that were overarching; reviewing of themes; definition, and renaming of themes; and report writing. Data organization and interpretation were grounded in the focus of the study. […] Through analysis of data thematically, nine themes relating to support practices for including learners with autism emerged.” |
| **Rigour** | “Upon completion of the preliminary data analysis, the researcher and two critical readers, who were experts in qualitative research, presented the primary themes that emerged to the participants for engagement and ultimately enhanced trustworthiness.” |
| **Stakeholder group(s)** | Teachers |
| **Recruitment** | “There are 766 regular public primary schools in Midlands educational province. To understand teachers’ support practices for including learners with autism in regular ECD classes, public primary schools were purposively sampled from institutions that were inclusive of these learners. The sample constituted 18 public primary schools. Six institutions were drawn from urban, semiurban, and rural settings, respectively. Recruitment of teachers was through contacts with Midlands Provincial Education Offices of Zimbabwe. The researcher distributed information letters to contacts in the designated schools. Upon the head teacher’s approval of the study, information letters were distributed to teachers who were perceived to satisfy the criteria for participation. The criteria for teachers’ participation in the study included the following: at least a primary school teacher’s diploma with an endorsement in ECD and an undergraduate degree in inclusive education or related field of specialization; at least teaching experience of 4 years in a regular ECD class, including a learner/learners with autism; and presently a teacher in a regular ECD class with a learner/learners with autism in Midlands educational province. A total of 18 purposively sampled ECD teachers made up of 11 females and seven males, one per participating institution, participated in the current study. Theoretical saturation informed the adequacy of the number of the study participants.” |
| **Participant information** | **Total number**  18 |
|  | **Number per stakeholder group**  18 teachers |
|  | **Demographics**  11 females, 7 males; 31-57 years old |
|  | **Other information**  “Each of the participants taught in a regular ECD class which had a maximum of 17 four- to five-year-old learners. Each regular ECD class included, at most, two learners who had high functioning autism whose intelligence ranged from average to above average. Participants were between 31 and 57 years old with five to 15 years of teaching experience. In addition to a primary school teacher’s diploma with specialization in ECD, 12 participants had postgraduate qualifications in special needs education. Each of them was a host teacher to a student teacher.” |
| **Relevance of DD and type** | Focus on inclusion for ASD |
| **Setting information** | **School grade and type**  “The current study was executed in regular public primary schools in the Midlands educational province.”  Early Childhood Development grades |
|  | **Contextual information**  “English is the medium of instruction in these institutions. Midlands educational province comprises public primary schools in rural, semiurban, and urban settings of Kwekwe, Gokwe North, Chirumhanzu, Zvishavane, Gokwe South, Gweru, Mberengwa, and Shurugwi districts. One institution was drawn from each one of these settings from each district.” |
| **Themes / topics** | “Through analysis of data thematically, nine themes relating to support practices for including learners with autism emerged. They are as follows: entry competencies informed pedagogy, structured routines, reinforcement, academic modifications, environmental modifications, socialization, communication, management of obsessions and compulsions, and collaboration and discourse.” |
| **Extraction method for Results and Discussion** | All extracted for analysis |
| **Quality** | Very Good |

### **Majoko (2018)**

| **Full Reference** | Majoko T. (2018). Inclusion of Children with Autism Spectrum Disorders in Mainstream Primary School Classrooms: Zimbabwean Teachers’ Experiences. *International Journal of Special Education*, 33(3):630–56. |
| --- | --- |
| **Authors** | Majoko, Tawanda |
| **Year** | 2018 |
| **Country/ies** | Zimbabwe |
| **Aim** | “Although a number of researchers explored teachers’ perceptions of their professional competence as regards teaching children with ASD internationally (Humphrey, & Lewis, 2008), this is the first study to be executed within the Zimbabwean education system. Embedded in qualitative methodology, the present study interrogated teachers’ experiences as a springboard for ascertaining the adequacy of their preparation through self-reported competence.” |
| **Objectives** | N/A |
| **Research question(s)** | “What are the experiences of teachers in inclusion of children with ASD in mainstream primary school classrooms in the Midlands educational province of Zimbabwe?; What issues do teachers confront in inclusion of children with ASD in mainstream primary school classrooms in Midlands educational province of Zimbabwe? and What systems and resources support the inclusion of children with ASD in mainstream primary school classrooms in Midlands educational province of Zimbabwe?” |
| **Methodology** | “In order to address the foregoing research questions, this study used a qualitative phenomenological framework as it enabled the researcher to capture the essences of meaning underlying how individual participants felt about their presented personal experiences (Bednall, 2006; Cohen, Manion & Morrison, 2007). A constant comparative approach was used to uncover the meanings participants attached to the ways in which they dealt with specific aspects of their existence (McMillan & Schumacher, 2006).” |
| **Data collection methods** | “In education, psychology and sociology, the use of an original semi-structured interview schedule in the generation of data for analysis is a common phenomenon (Cohen et al., 2007; Creswell, 2009). This study used interview schedules (see Table 1) based on a review of interview schedules developed previously within the disability literature. These include Eldar et al. (2010), Humphrey & Lewis (2008), McGillicuddy & O’Donnell (2013) and Lindsay et al. (2013). […] Its final version constituted seven questions.” […]  “Interviews were conducted in English in a quiet area of each participant’s school on interview appointment days at a time outside participants’ regular class teaching periods. Before the onset of the interview, each teacher was reminded of the voluntary nature of their participation, the anonymity of all of their responses, their right not to answer any question that they felt were not comfortable with and informed of their right to withdraw from the interview session at any point although none did so. With participants’ informed consent, an audio recorder was used in each interview session. No time limit was set in order to ensure consistency in the process of data collection. The interviews ranged from 45 minutes to 80 minutes.” |
| **Data analysis methods** | “Consistent with the phenomenological approach, inductive thematic content analysis, which is a process utilised to identify and analyse patterns and themes, was used in data analysis (Babbie & Mouton, 2011). In the first level of analysis, the researcher and the two critical readers, who were experts in qualitative research, independently examined individual participant transcripts to determine recurring themes. In order to identify both unique and common themes (Grbich, 2007), the second level of analysis entailed identification of themes across transcripts. Data analysis sought to acquire a sense of overall meaning instead of frequencies (Cohen et al., 2007). In alignment with the qualitative approach, direct quotes were utilised as much as possible in order to entrench study findings and interpretations (Creswell, 2009).” |
| **Rigour** | “Trustworthiness and accuracy issues were addressed numerous times throughout study. In order to maintain consistency with the research questions, the researcher and the critical readers, began by classification of the statements according to the research questions of the study. The researcher and the critical readers also independently analysed the data so as to address the threats of the accuracy of interpretation. The researcher and the critical readers, thereafter, jointly discussed their analyses which resolved discrepancies through reviewing relevant data. Further, accuracy of interpretation was addressed through colleague checks. Additionally, participants were requested to review the transcripts and, when necessary, add further explanatory information.” |
| **Stakeholder group(s)** | Teachers |
| **Recruitment** | “Twenty-four (15 male and nine female) teachers […] participated in the study. They were purposively drawn from three public primary schools. […] All 24 participants met the inclusion criteria to participate in the study particularly (1) at least an undergraduate qualification with endorsement in primary school education; (2) at least five years’ experience in teaching within mainstream primary school classrooms; (3) at least two years’ experience in teaching children with ASD in mainstream primary school classrooms; and (4) are presently teaching in a mainstream primary school classroom with a child/children with ASD.” […]  “Participants for this study were purposively sampled through contacts with Midlands provincial education offices. In order to establish eligibility and to schedule interview settings and times, individual participants were screened by telephone. Informed consent was sought and obtained from the participants prior to the onset of the interviews.” |
| **Participant information** | **Total number**  24 teachers |
|  | **Number per stakeholder group**  N/A |
|  | **Demographics**  15 males and 9 females |
|  | **Other information**  “The mainstream teaching experience of the participants ranged from seven to over 21 years and their experience in teaching children with ASD ranged from four years to thirteen years.” |
| **Relevance of DD and type** | “Inclusion of Children with Autism Spectrum Disorders in Mainstream Primary School Classrooms”  “Participants based their interview question responses on five female and nineteen male children with ASD that they were teaching.” |
| **Setting information** | **School grade and type**  “Twenty-four (15 male and nine female) teachers, comprising three Early Childhood Education and Development, two Grade 1, four Grade 2, three Grade 3, two Grade 4, five Grade 5, one Grade 6 and four Grade 7, participated in the study. They were purposively drawn from three public primary schools” |
|  | **Contextual information**  “They were purposively drawn from three public primary schools located in low to high socio-economic statuses settings particularly farm, rural and urban areas in the Midlands educational province of Zimbabwe. Each school had an average enrolment of 600 children.” |
| **Themes / topics** | 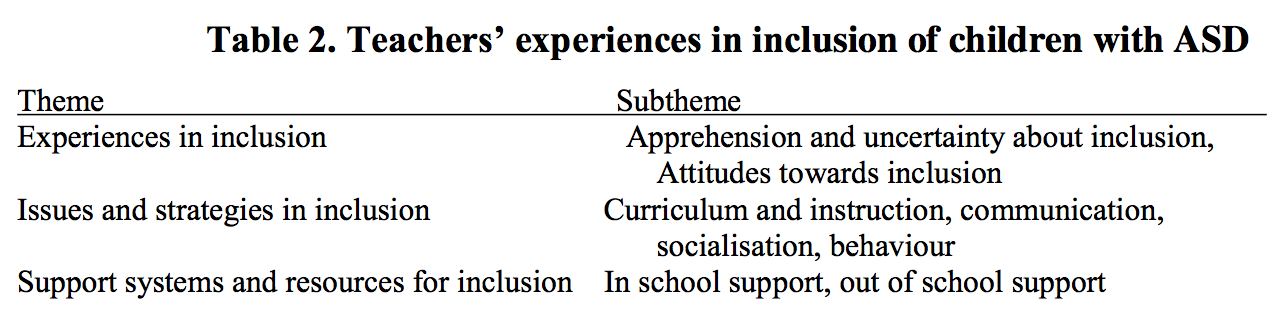 |
| **Extraction method for Results and Discussion** | All extracted for analysis |
| **Quality** | Very Good |

###

### **Majoko (2019)**

| **Full Reference** | Majoko, T. (2019). Teacher key competencies for inclusive education: Tapping pragmatic realities of Zimbabwean special needs education teachers. *Sage Open*, *9*(1), 2158244018823455. <https://doi.org/10.1177/2158244018823455> |
| --- | --- |
| **Authors** | Majoko, Tawanda |
| **Year** | 2019 |
| **Country/ies** | Zimbabwe |
| **Aim** | “The current study was perhaps the first of its kind in Zimbabwe that used qualitative research methodology to interrogate teachers’ perceptions on key competencies for inclusive education.” |
| **Objectives** | N/A |
| **Research question(s)** | “Specifically, this study addressed the following research question:  Research Question 1: What are the teaching competencies special educational needs teachers in Midlands educational province of Zimbabwe perceive as key for inclusive education?” |
| **Methodology** | “This study utilized interpretive qualitative research methodology. Qualitative research methodology enabled the researcher to solicit detailed data from a small group of informants (Cohen, Manion, & Morrison, 2007; Creswell, 2009; McMillan & Schumacher, 2006). The use of qualitative research methodology was also influenced by the researcher’s focus, concern with process instead of outcome, descriptive nature of the data, essential concern with meaning, and inductive analysis of data (Grbich, 2007; Pierce, 2008; Silverman, 2009).” |
| **Data collection methods** | “An individual interview is a fundamental instrument for the qualitative researcher because it allows participants to express their perceptions and opinions about a phenomenon being investigated using their own words (Cohen et al., 2007; Pierce, 2008; Silverman, 2009). The interview protocol for this study constituted open-ended, semi-structured questions that explored teachers’ perceptions regarding key competencies for successful inclusive education. […] All interviews were audiotaped and transcribed verbatim with the consent of the individual participants. […] All the interviews were executed in English in participants’ classrooms after school hours with their voluntary participation and without any compensation. Despite the organization and structure during interviews, as a result of the use of the interview guide, flexibility was ensured through the interviewer’s context-specific questioning of issues raised by the interviewees. The individual interviews were executed between November 2016 and August 2017, each lasting on average 75 min.” |
| **Data analysis methods** | “Upon its completion, each tape-recorded individual interview was transcribed verbatim. Individual interviews were read many times and coded individually by the researcher and two critical readers who are experts in qualitative data analysis. Cross-case analysis was subsequently executed for all individual interviews. Pattern coding was conducted in alignment with the suggestion of Miles and Huberman (1994). In pattern coding, the researcher and the two critical readers reviewed the interview transcripts and cross-checked the emerging themes from each individual interview while identifying emerging patterns grounded in collective consensus (Pierce, 2008). Contrasting or negative cases and frequencies of themes were attended to during the process of coding (Grbich, 2007). Emerging themes were subsequently organized around the research question of the study. Within and across case analyses, the researcher and the critical readers endeavored to understand how teachers explained and contextualized their narratives.” |
| **Rigour** | “Data were member checked to establish rigor and trustworthiness in the findings (Cohen et al., 2007; Creswell, 2009; Silverman, 2009). This was achieved by giving interview transcripts to individual interview participants who read the transcription of the interview to validate its content and the meanings derived from it. Analysis of data was a reciprocal process among the researcher and the two critical readers. The critical readers were presented with the whole set of analysis documents to examine its credibility.” |
| **Stakeholder group(s)** | Teachers |
| **Recruitment** | “A sample of 24 public primary schools, purposively selected from urban, semi-urban, and rural settings of the respective districts in Midlands educational province, participated in this study. Teachers were recruited through contacts with Midlands provincial education offices. Information letters were distributed to contacts in the designated institutions. Upon the head teachers’ approval of the execution of the study, the researcher distributed information letters to teachers who were perceived to meet the inclusion criteria below. Teachers who were interested in taking part in the study contacted the researcher to organize a convenient time to be individually interviewed. One teacher was purposively drawn from each of the urban, semi-urban, and rural settings of the respective districts in Midlands educational province to aid transferability of findings. To ascertain eligibility for participation in the study and to schedule interview time, individual teachers were screened over the telephone. The inclusion criteria for the teachers comprised at least a primary school teachers’ diploma; a bachelor’s degree in special needs/special education/inclusive education; teaching experience of 5 years in regular primary school classrooms, including a child/children with disabilities; currently teaching in a public regular primary school classroom that includes a child/children with disabilities in Midlands educational province; and informed consent to participate.” […]  “Theoretical saturation, which occurred when no relevant or new data emerged, regarding a category and categories were well developed with respect to their properties, dimensions, and variations and determined the adequacy of the sample (Cohen et al., 2007; Silverman, 2009).” |
| **Participant information** | **Total number**  24 |
|  | **Number per stakeholder group**  24 teachers |
|  | **Demographics**  “18 males, 6 females” |
|  | **Other information**  “Each one of them taught a regular class that had a maximum of forty-one 5- to 12-year-old children without assistant teachers.” […]  “All the participants had 3-year primary school teachers’ diplomas which constituted theory of education, a main subject, professional studies, and teaching practice. Inclusive education was infused in disciplines of theory of education and professional studies, in particular psychology of education and subject didactics. Participants had trained in categories of disabilities, behavior management, classroom management, collaboration, curriculum differentiation, national and international policies and legislation on inclusive education, and curriculum management in inclusive education. The overall philosophy underpinning their inclusive education training was “quality education for all.” All participants had also obtained a Bachelor of Education (Special Needs Education) Degree. This was a 2-year program offered to in-service teachers who had at least a 3-year mainstream primary school teachers’ diploma from any of the associated teachers colleges of the University of Zimbabwe. […] Participants’ teaching experience ranged from 7 to 15 years and they were aged between 31 and 58 years.” |
| **Relevance of DD and type** | Among the disability categories in the classrooms there are attention deficit hyperactivity disorder, learning disabilities, and emotional/behavioural disabilities.  Results consistently refer to cognitive and learning impairments, developmental delays and disorders and behavioural challenges. |
| **Setting information** | **School grade and type**  “This study was carried out in selected regular primary schools in Midlands educational province.” […]  “Three teachers per grade from Early Childhood Development class to Grade 7.” |
|  | **Contextual information**  “These schools use English as medium of instruction although local languages, namely, Shona and Ndebele, are sometimes used in teaching and learning for ease of understanding. Midlands educational province constitutes primary schools in the urban, semi-urban, and rural settings of Shurugwi, Mberengwa, Gweru, Gokwe North, Gokwe South, Zvishavane, Chirumhanzu, and Kwekwe districts (Munjanganja & Machawira, 2015).” […]  “These classes were inclusive of children with at least at three of the following disabilities: seizures, attention deficit hyperactivity disorder, learning disabilities, visual impairment, physical disabilities, hearing impairment, and emotional/behavioral disabilities. These children had low to medium level of support needs as diagnosed by the Department of School Psychological Services and Special Needs Education of Zimbabwe as revealed in teachers’ record books. The Department of School Psychological Services and Special Needs Education of Zimbabwe provide in-service training on inclusive education to these teachers. The teachers also attend at least one staff development session of inclusive education per term at their schools. They further invite resource persons to provide staff development on topics in inclusive education that they perceive to be challenging in their management of daily teaching and learning in regular classrooms.” |
| **Themes / topics** | Screening and Assessment  Differentiation of Instruction  Classroom and behavior Management  Collaboration |
| **Extraction method for Results and Discussion** | All extracted for analysis |
| **Quality** | Very Good |

### **Mangope (2017)**

| **Full Reference** | Mangope, B. (2017). Inclusive practices for learners with intellectual disabilities in primary schools in Botswana: What are teachers doing to enhance inclusion. *Mosenodi Journal*, *20*(1), 32-47. |
| --- | --- |
| **Authors** | Mangope, Boitumelo |
| **Year** | 2017 |
| **Country/ies** | Botswana |
| **Aim** | “the aim of this study was to find out the sort of inclusive education teaching strategies employed to cater for students with ID in primary schools in Botswana. The vital intent of this study was to improve programming efforts in inclusive education in Botswana.” |
| **Objectives** | N/A |
| **Research question(s)** | “(a) What knowledge of inclusive education teaching strategies for students with ID do special education teachers have?  (b) How do teachers employ inclusive teaching strategies in the special unit classrooms to teach students with ID?” |
| **Methodology** | “The Interpretivist paradigm guided this research. Interpretivism contends that reality is not independent but is socially constructed and can have varied meanings. Thus knowledge is constructed through interaction of people (researchers included) and objects of inquiry (Lotz-Sisitka, Fien & Ketlhoilwe 2013). In other words, the findings of this research were a culmination of the interaction that the researcher had with special education unit teachers. This enabled the researcher to understand their meaning of ‘inclusive education’ and their interpretation of how they implement government policy and pedagogical practices applicable to inclusive education. Interpretivism supports the use of case studies, narrative inquiry and interviews as used in this study to understand inclusive education as a phenomenon.” […]  “This study used a qualitative research methodology in the form of a multiple case study research design. Merriam (1998) posits that in order to derive an in-depth understanding of a situation and its contextual factors, a case study design is often the most appropriate, which in this case is inclusive education, learners and teachers. Each special unit classroom became a case and hence multiple case sites were investigated within the four schools.” |
| **Data collection methods** | “Since this study focused on understanding inclusive education as a phenomenon, the researcher had to understand the participants’ point of view, and hence allowed them to tell a story in their own words. This made interviews a very important data collection method. As noted by Long and Godfrey (2004), qualitative research gathers and analyses data using visual and verbal (conceptual or thematic) techniques, thus explaining why it became a method of choice. This is why methods such as interviewing, observation and document analysis (analysis of policies, classroom documents) were all employed in this research.” […]  “Data was collected using semi-structured interviews, observation checklists and document analysis. An interview instrument, entailing 30 semi-structured items was adopted to collect data from all teachers. The instrument was developed through an examination of studies concerning inclusive teaching practices for students with intellectual disabilities and was further checked by colleagues in the special education section of the University of Botswana to verify its trustworthiness. All the eight teachers were interviewed face-to-face to gain insights on the inclusive intervention strategies practiced in the classrooms. An interview for each teacher was audio recorded and lasted about fifty minutes.  Classroom observations were also conducted for each teacher in each school using an adapted Effective Teaching Observation checklist by Kuyini (2004). The main aim was to find out how teachers implement inclusive practices in their classrooms; that is, what sort of activities they did, and how they were implementing the curriculum. One special unit classroom from each school was observed twice. Moreover, informal follow-up discussions were held with each teacher about their lessons for further illumination.  Document analysis of government policy documents, lesson plans, Individualized Educational Programs (IEPs) and class tests was also used to extract information on inclusion.” |
| **Data analysis methods** | “Content analysis was used to analyse data. The audio-recorded data was first transcribed word-for-word by the researcher. The transcripts were then thoroughly analysed, creating themes and categories. Themes were subsequently developed through a constant comparison method of data analysis (Strauss & Corbin, 1990). No software was employed to analyse the data since the sample size was manageable. As an initial step, interview items were paraphrased to produce an outline of the transcripts. This helped in compressing chunks of data into manageable items. Each transcript was then studied distinctly in the second step, and new themes were underlined every time they appeared. Step three involved further identification and comparing of themes within and across the transcripts. Ultimately, the fourth and final step entailed the development of the major themes.” |
| **Rigour** | “The different data collection methods formed a methodological triangulation, which made the data more trustworthy and credible as one source was checked against the other (McMillan & Schumacher, 1993, p. 497; Lincoln & Guba, 1985, p. 283).” […]  “With regard to credibility of the findings addressed in this study, the researcher used member checks to confirm trustworthiness. This was done by allowing the participants to confirm or disconfirm their interpretations after a preliminary analysis of data had been conducted. This enhanced a better understanding of the data and aided in precise interpretation of the ideas. Moreover, audio recordings were used to cross check the data several times to enhance credibility. This was also carried out through discussions with colleagues in the Special Education Section of the University of Botswana.” |
| **Stakeholder group(s)** | Special education teachers |
| **Recruitment** | “Eight special education teachers who are teaching in the special education unit classrooms were purposively selected from the four identified government primary schools in the South Central section of Botswana. According to the teaching establishment register (Government of Botswana, 2008), each school with a unit classroom should have a maximum of two qualified special education teachers, and thus two teachers per school participated in the study.” […]  “Given the small population of special education teachers per district in Botswana, and that the aim of the study was not to generalise but to learn and understand, the eight teacher participants were sufficient for the study.” |
| **Participant information** | **Total number**  8 |
|  | **Number per stakeholder group**  8 teachers |
|  | **Demographics**  “The teachers in the study had an age range of 35–45.” |
|  | **Other information**  “All of the participants selected for the study had more than five years of teaching experience in the unit classrooms, had minimum qualifications of a diploma in special education, and most of them had acquired their training from the 1990s to the early 2000s.” |
| **Relevance of DD and type** | Focus on ID |
| **Setting information** | **School grade and type**  general education government primary schools |
|  | **Contextual information**  “Four special education unit classrooms from four general education government primary schools in South Central section of Botswana were chosen purposively. Each school was selected from urban, semi-urban, rural and remote areas. The criteria or bracketing that was used in the selection was that the unit program must have been in operation for at least five years or more, must be within a government-funded primary school; and the program should be for students with intellectual disabilities.” |
| **Themes / topics** | “lesson planning and presentation, adaptive instruction, curriculum, accommodations, effective inclusive strategies and experiences” |
| **Extraction method for Results and Discussion** | All extracted for analysis |
| **Quality** | Average |

### **Mangope et al. (2018)**

| **Full Reference** | Mangope, B., Otukile-Mongwaketse, M., Dinama, B., & Kuyini, A. B. (2018). Teaching Practice Experiences in Inclusive Classrooms: The Voices of University of Botswana Special Education Student Teachers. *International Journal of Whole Schooling*, *14*(1), 57-92. |
| --- | --- |
| **Authors** | Mangope, Boitumelo  Otukile-Mongwaketse, Mpho  Dinama, Baamphatlha  Kuyini, Ahmed Bawa |
| **Year** | 2018 |
| **Country/ies** | Botswana |
| **Aim** | “The purpose of this study was to explore the teaching practice experiences of the University of Botswana special education student teachers, with the aim to understand the challenges they experienced in the teaching of learners with special needs in inclusive Botswana secondary school classrooms” |
| **Objectives** | N/A |
| **Research question(s)** | “The following research questions guided the study:  1. What are the experiences of the student teachers in relation to teaching in inclusive  secondary schools’ classrooms?  2. How do these experiences impact their beliefs and attitudes (toward) about the teaching practice program at the University of Botswana?” |
| **Methodology** | “A qualitative research approach was considered suitable because this research is an exploratory study aimed at gaining understanding of student teachers’ experiences during teaching practice in inclusive classrooms. Given the focus on student teachers’ views and experiences, it was possible to construe the methodological approach of the study as phenomenological in nature. In general, phenomenological research aims at clarifying individuals’ situations in everyday life (Giorgi & Giorgi, 2003).” |
| **Data collection methods** | “Following informed consent and reassurance of confidentiality and anonymity, data were collected through in-depth semi-structured face-to-face interviews and two focus group discussions. Both the individual and group interview questions focused on student teachers’ experiences of teaching practice, particularly in inclusive classrooms and how such experiences impacted on their attitudes and beliefs about the TP program at UB. The interviews lasted between 45 to 60 minutes, while focus groups lasted between 1 and 1.5 hours each and were audio taped and later transcribed verbatim. The interviews aimed at eliciting student teachers’ personal views on the teaching practice as a learning experience and to give suggestions for improving this practical activity.” |
| **Data analysis methods** | “The data was analysed using content analysis of transcribed textual data. This was done in stages. In the first stage the researchers transcribed data from all the audio-tapes verbatim. Data were then consolidated into three broad categories, namely 'positive experiences’, ‘negative experiences’ and ‘impact on the attitudes’. During the second stage, the researchers read through the transcribed interviews to familiarize themselves with the transcript contents. In the third stage, researchers identified, classified, organized, and encoded sections of the interview that were identified as major themes. In the fourth stage, data were synthesized in order to identify commonalities within similar units of meaning identified within each theme (Merriam, 1988). In the last stage of analysis, themes were compared in relation to positive and negative experiences, impacts on attitudes, and connections were drawn between them in order to gain a holistic picture.” |
| **Rigour** | “Member checking was used to ensure the trustworthiness of the information gathered from participants (Merriam, 1998). In this case, participants were given access to the data analysis records and asked to give feedback, rectify any errors or provide more information in instances where they felt they were misrepresented. This process helped to confirm or disconfirm the consistency of the interpretations derived from the data (Merriam, 1998).” |
| **Stakeholder group(s)** | Student teachers |
| **Recruitment** | “A purposive sampling technique was used to select schools that were regarded as “inclusive”. The aim was to have information-rich cases, which could provide meaningful insights. Bearing in mind that there were no formally accepted criteria for “inclusive schools” in Botswana, a six-member panel of experts consisting of two special education government officials, two special education teacher trainers from the four colleges of education and two special education lecturers from the University of Botswana were involved in the selection of the schools which they perceived as being “inclusive”. Among the panel members, a strong consensus emerged and a list of six schools was provided as being inclusive. Four of the six schools were selected.” […]  “The participants for the present study consisted of 23 student teachers in the third and fourth year of the special education program purposively drawn from the four schools in the southern region of the country.” |
| **Participant information** | **Total number**  23 |
|  | **Number per stakeholder group**  23 student teachers |
|  | **Demographics**  9 males, 14 females; aged 21-25 |
|  | **Other information**  “They were all enrolled in the B. Ed Special Education program with specialisation in Intellectual Disabilities, Learning Disabilities, Visual and Hearing impairments. The researchers engaged third -year and fourth - year students because they had been engaged in continuous teaching practice in inclusive classrooms for more than a year and were therefore likely to provide more informed and considered responses to the questions.” |
| **Relevance of DD and type** | 5 teachers (22%) specialising in ID and 11 (48%) in learning disabilities |
| **Setting information** | **School grade and type**  “This study was carried out in selected junior secondary schools located in the Southern region. The junior secondary schools were chosen because this particular cohort of student teachers was the first group of pre-service teachers being purposely prepared to teach in junior secondary schools. No research about pre-service TP has focused solely on this group before.” |
|  | **Contextual information**  “The sampled schools were homogeneous in terms of the fact that they had learners with special needs who transited from various primary schools with special units classrooms and were now included in the regular secondary school classrooms. Two schools were located in urban and the other two schools were located in semi-urban settings. The four schools had been practicing inclusion for more than three years. All selected schools had student populations of about 600 students, with an average of 2-3 learners with special educational needs in each classroom.” |
| **Themes / topics** | The following major themes emerged from the data analysis: Exposure to real life situations, limited skills and pedagogical knowledge, mentor teachers’ negative attitudes, relevance of the portfolios, and large class sizes. |
| **Extraction method for Results and Discussion** | All extracted for analysis |
| **Quality** | Very Good |

### **Mapuranga et al. (2015)**

| **Full Reference** | Mapuranga, B., Dumba, O., & Musodza, B. (2015). The Impact of Inclusive Education (IE) on the Rights of Children with Intellectual Disabilities (IDs) in Chegutu. *Journal of Education and Practice*, *6*(30), 214-223. |
| --- | --- |
| **Authors** | Mapuranga, Barbra  Dumba, Oswald  Musodza, Blessing |
| **Year** | 2015 |
| **Country/ies** | Zimbabwe |
| **Aim** | “The inclusion of children with intellectual disabilities (IDs) in regular schools prompted the research to investigate the impact of inclusive education (IE) on the rights of ID children in Zimbabwe’s secondary schools.” |
| **Objectives** | N/A |
| **Research question(s)** | N/A |
| **Methodology** | “The study used both quantitative and qualitative approaches to investigate the phenomenon under study. It focused on the impact of inclusive education on children with ID in secondary schools. The respondents who used both the questionnaires and interviews as instruments to gather and other school children, in this regard the study concerned with the new and perceptions of parents, teachers and children on the impact of IE in as far as children with ID is concerned. Masuku (1999:24) argues that ‘qualitative research design has the natural setting as the direct source of information the conclusion are arrived at after considering any recurrent patterns which may emerge’. In view of this qualitative research was the best method to use” |
| **Data collection methods** | “The respondents who used both the questionnaires and interviews as instruments to gather and other school children, in this regard the study concerned with the new and perceptions of parents, teachers and children on the impact of IE in as far as children with ID is concerned.” […]  “For the research to answer the main research question, the investigation had four research questions which were answered by the respondents in the questionnaires and interviews during data collections.” |
| **Data analysis methods** | N/A |
| **Rigour** | N/A |
| **Stakeholder group(s)** | Teachers, children, parents |
| **Recruitment** | “Random sampling was used to choose the sample group from a total population of 80.” […]  “Thirty respondents […] from the five secondary schools constituted the sample group.” |
| **Participant information** | **Total number**  30 |
|  | **Number per stakeholder group**  10 teachers, 10 children and 10 parents |
|  | **Demographics**  N/A |
|  | **Other information**  N/A |
| **Relevance of DD and type** | Intellectual disabilities |
| **Setting information** | **School grade and type**  “five secondary schools” |
|  | **Contextual information**  N/A |
| **Themes / topics** | Question1: What is your understanding of children with IDs?  Question 2: Which rights are being observed by employing I.E?  Question 3: What challenges are faced after the recognition of the right of children with disabilities in I.E? |
| **Extraction method for Results and Discussion** | All extracted for analysis |
| **Quality** | Very Poor |

### **Matsenjwa et al. (2020)**

| **Full Reference** | Matsenjwa, H., Ntinda, K., & Makondo, D. (2020). Teachers’ experiences of learners with intellectual disabilities in primary schools of Eswatini. *UNESWA Journal of Education (UJOE)*. |
| --- | --- |
| **Authors** | Matsenjwa, Hlob’sile  Ntinda, Kayi  Makondo, Davison |
| **Year** | 2020 |
| **Country/ies** | Eswatini |
| **Aim** | “The study sought to explore the teachers’ experiences of learners with intellectual disabilities (IDs), collaborating with parents in mainstream primary schools in the Lubombo region in Eswatini.” |
| **Objectives** | N/A |
| **Research question(s)** | “What collaboration strategies do teachers of learners with IDs use with parents in mainstream primary schools of Siteki, in the Lubombo region of Eswatini?” |
| **Methodology** | “The phenomenological research design which creates an opportunity for multiple socially constructed worldviews (Creswell, 2013) was employed in this study. The phenomenological design allows for a deep understanding of commonality of lived experiences within a particular group and provides abundant data about real life of people (Maxwell, 2013; Leedy & Ormrod, 2014). The approach was utilized because it provided an opportunity to explore the innermost deliberation of the lived experiences of the participants on the phenomenon under study (Alase, 2017).” |
| **Data collection methods** | “Focus group discussions and in-depth interviews were used to collect data, and interview guides were generated and used to guide the collection of data (Vosloo, 2014). All interviews were conducted in English though participants were allowed to respond in both English and SiSwati. All the participants took part in the Focus group discussions. Three (3) focus group discussions comprising of 8 participants each were conducted in three (3) different schools for an average of sixty (45) minutes per session to identify trends in the different views of the participants (Daniel, 2016). […] Probe stems were used to ask participants to describe aspects that constituted their work collaboration with parents in support of learners with IDs. Participants who provided detailed responses were recruited for individual in-depth interview and 12 teachers participated. In-depth interviews created opportunities for emerging questions and teacher participants were encouraged to feel free to give elaborate responses that enhanced data collection (Jamshed, 2014). In-depth interviews were also conducted in three (3) conveniently selected schools and lasted for approximately 30 minutes per interview. All interviews were audio-recorded and then transcribed.” |
| **Data analysis methods** | “Participants’ responses were coded and recorded in accordance with the interview guide” […]  “The researchers conducted data analysis concurrently for both the focus group discussions and in-depth interviews. Data analysis was done on regular bases immediately after each interview session. Thematic analysis by Braun and Clarke (2006) was used and it allowed for an opportunity to identify patterns and themes of the phenomenon beyond the participants’ perceptions (Rabinovich & Kacen, 2013). This enabled the researchers to become intimately familiar with the data by reading and re-reading the data; and listening to audio recorded data at least once if relevant (Braun & Clarke, 2012). Initial codes were identified and compared. Then similar codes were grouped into categories that led into the development of one major theme and sub-themes which were crucial for the purpose of the study.” |
| **Rigour** | “Member checks with the participants were done in this study to observe credibility and the accuracy of responses from each participant (Khan, 2014). The process of member checking gave the participants an opportunity to correct flaws from responses, comments, and to interpret and solidify some of the findings. In addition, participants were encouraged to be as honest as possible and open during discussions to ensure dependability. There was consistent recording and reporting during the discussions.” |
| **Stakeholder group(s)** | Special teachers |
| **Recruitment** | “teacher participants were sampled from six (6) conveniently selected mainstream primary schools in Siteki, Eswatini. […] teachers that met the inclusion criteria participated in the study. […] Purposive sampling technique was employed to select participants based on their, teaching experience, qualification and training. The inclusion criteria were having: at least two or more years teaching experience, Primary Teachers’ Diploma with specialisation in Special Education Needs Training and teaching learners diagnosed with IDs (6 - 12 years).” […]  “Participants who provided detailed responses were recruited for individual in-depth interview and 12 teachers participated.” |
| **Participant information** | **Total number**  24 (12 interviews) |
|  | **Number per stakeholder group**  24 teachers |
|  | **Demographics**  15 female; 9 male  10 aged 25-35, 10 aged 26-36, 4 aged 47-60 |
|  | **Other information**  “the inclusion criteria were having: at least two or more years teaching experience, Primary Teachers’ Diploma with specialisation in Special Education Needs Training and teaching learners diagnosed with IDs (6 - 12 years).”  21 with 2-5 years SEN experience, 3 with 6+ experience |
| **Relevance of DD and type** | Focus on ID |
| **Setting information** | **School grade and type**  mainstream primary schools |
|  | **Contextual information**  “Siteki, in theLubombo region of Eswatini” |
| **Themes / topics** | Collaboration strategies (Journaling, Personal skills development, Creation of time for school work, Provision of basic needs) |
| **Extraction method for Results and Discussion** | All extracted for analysis |
| **Quality** | Average |

### **Mohamed & Laher (2012)**

| **Full Reference** | Mohamed, Z., & Laher, S. (2012). Exploring foundation phase school teachers’ perceptions of learning difficulties in two Johannesburg schools. *Journal of Child & Adolescent Mental Health*, *24*(2), 133-147. <https://doi.org/10.2989/17280583.2012.735500> |
| --- | --- |
| **Authors** | Mohamed, Zaakirah  Laher, Sumaya |
| **Year** | 2012 |
| **Country/ies** | South Africa |
| **Aim** | “If teachers are to identify learners with LD they must understand it. This is important as this study investigated primary school teachers’ understanding of LD to help improve the education of learners with LD.” |
| **Objectives** | N/A |
| **Research question(s)** | N/A |
| **Methodology** | “This study adopted a broad based outlook on teachers’ perception of LD as it explored the general perceptions of the teachers. Thus a qualitative methodology was most appropriate.” |
| **Data collection methods** | “Semi-structured interviews were conducted with the teachers. The interview schedule consisted of 17 primarily open-ended questions. The questions were developed from the literature reviewed. The interview schedule was designed to explore the teacher’s perceptions of LD and the issues relating to these perceptions. Five questions aimed at contextualising teacher experience. Four questions focussed on teacher’s understanding of LD. Two questions explored the impact LD has on learners and parents. The final six questions focussed on teacher responses to LD. The interview schedule was piloted using two lecturers familiar with the LD field and changes were made based on the pilot. […] Each interview was approximately 45–60 minutes long. All of the interviews were tape-recorded.” |
| **Data analysis methods** | “The data were analysed using thematic content analysis as described by Braun and Clarke (2006). As per the analysis method, the initial step involved reading and rereading the transcripts. Initial codes were generated from the data. The codes were then combined to form themes which were refined, defined and named. The themes were then interpreted, as described in the results and discussion sections.” |
| **Rigour** | N/A |
| **Stakeholder group(s)** | Teachers |
| **Recruitment** | “The study used a non-probability convenience sample of eight foundation phase teachers working in two mainstream government schools in the Johannesburg West region.” […]  “Permission was also requested from the principals of the schools. Information was made available to the teachers regarding the study at a staff meeting and individuals were asked to volunteer to participate in the study. All teachers who volunteered were contacted to arrange a convenient time for them to be interviewed.” |
| **Participant information** | **Total number**  8 |
|  | **Number per stakeholder group**  8 teachers |
|  | **Demographics**  “All eight participants were Black, female teachers.” |
|  | **Other information**  “Six of the teachers’ interviewed had over 10 years of teaching experience. Two of the teachers interviewed have taught for three years or less. […] Six of the teachers had only taught foundation phase. One teacher taught intermediate phase as well and another teacher was trained as a high school art teacher.” |
| **Relevance of DD and type** | Focus on inclusion for LD:  “Learning difficulties/disorders/disabilities may be viewed on a continuum from less to most severe. Given this, we use the abbreviation LD throughout the study to include this continuum.  Children with LD have common characteristics such as poor learning strategies, perceptual and information processing, mathematics and language (spoken and written) difficulties and inability to pay attention (Lerner 1997).” […]  “All of the teachers had taught learners with LD.” |
| **Setting information** | **School grade and type**  “two mainstream government schools in the Johannesburg West region. […] Two of the participants were Grade 1 teachers, three of the participants were Grade 2 teachers and three of the participants were Grade 3 teachers.” |
|  | **Contextual information**  N/A |
| **Themes / topics** | Seven themes emerged from coding the data using thematic content analysis. These themes were: understanding of LD, aetiologies of LD, symptoms of LD, impact LD has on a learner, stigmatisation of learners with LD, degree of parental participation and inclusive education. |
| **Extraction method for Results and Discussion** | All extracted for analysis |
| **Quality** | Average |

### **Mokobane (2011)**

| **Full Reference** | Mokobane, S. Z. (2011). The academic engagement of intellectually challenged learners in inclusive schools: a case study. *Cypriot Journal of Educational Sciences*, *6*(2), 83-90. |
| --- | --- |
| **Authors** | Mokobane, Sonti Zelma |
| **Year** | 2011 |
| **Country/ies** | South Africa |
| **Aim** | “The purpose of the study is to explore intellectually challenged learners’ engagement in inclusive schools and to suggest strategies that can improve their effective engagement in inclusive schools.” |
| **Objectives** | N/A |
| **Research question(s)** | N/A |
| **Methodology** | “The qualitative single case study type was adopted for this paper because it helps in answering questions about the complex nature of the phenomena, often with the purpose of describing and understanding the phenomenon from the participant’s point of view (Greig & Taylor, 1999). The fact that it looks at a single case, namely an inclusive school in a rural area, helps in understanding a complex nature of a phenomenon, in this case the academic engagement of intellectually challenged learners.” |
| **Data collection methods** | “Data was collected using focus group interviews and one-on-one interviews with teachers. Learners were asked to complete diaries and one-on-one interviews were also conducted with learners. The interviews were conducted in the selected school and took place after school activities. There were questions to guide the interviews, which were recorded on audio tape with the participants’ permission.” |
| **Data analysis methods** | “Data was analyzed following the spiral method of Creswell(1994), taking the following steps  a) Organize data: Large bodies of text were broken down into different concepts, to try to get sense of possible themes.  b) Perusal: Data gathered was perused to get a sense of what it contained, jotting down memos and simultaneously classifying and pairing similar concepts.  c) Classification: Classification of data was carried out according to the following themes:  I. Engagement  II. Factors that contributes to the engagement of differently-abled learners.  III. Strategies that can be used to improve the engagement of differently-abled learners.  d) Synthesis: Data was gathered and integrated, also looking at the triangulation of data, for readers and drawing conclusions.” |
| **Rigour** | N/A |
| **Stakeholder group(s)** | Teachers and “intellectually challenged learners” |
| **Recruitment** | “The selection of participants was purposive because participants were identified and known. As stated by Krathwohl (1993), purposive sampling is assembled by intentionally seeking individuals for a situation likely to yield new instances and greater understanding of a dimension or concept of interest. Participation was voluntary, following a thorough explanation of the study and research ethics, namely participants’ rights to withdraw from the study and their being made aware that there was no remuneration for participating. Consent was obtained from parents allowing their children to participate and their confidentiality was assured.” |
| **Participant information** | **Total number**  20 |
|  | **Number per stakeholder group**  10 teachers, 10 learners |
|  | **Demographics**  N/A |
|  | **Other information**  N/A |
| **Relevance of DD and type** | Focus on including pupils with ID, who are also included as participants |
| **Setting information** | **School grade and type**  “an inclusive school that admits differently-abled learners (learners with disabilities), including those with intellectual disabilities. The school consists of grades 7, 8 and 9” […]  “a middle school, in Tshwane-North, Gauteng province” |
|  | **Contextual information**  “The research was carried out in a school regarded as a model of inclusion. Situated in Gauteng province, in the northern part of Tshwane, it is an inclusive school that admits differently-abled learners (learners with disabilities), including those with intellectual disabilities. The school consists of grades 7, 8 and 9; is situated in a semi-rural area and employs 36 educators, including the principal, two deputy principals and six heads of department.” |
| **Themes / topics** | “Classification of data was carried out according to the following themes:  I. Engagement  II. Factors that contributes to the engagement of differently-abled learners.  III. Strategies that can be used to improve the engagement of differently-abled learners.”[…]  Themes:  Immediate Feedback  Language of Instruction  Teaching Strategies |
| **Extraction method for Results and Discussion** | All extracted for analysis |
| **Quality** | Poor |

###

### **Mukhopadhyay et al. (2019)**

| **Full Reference** | Mukhopadhyay, S., Mangope, B., & Moorad, F. (2019). Voices of the voiceless: Inclusion of learners with special education needs in Botswana primary schools. *Exceptionality*, *27*(3), 232-246. <https://doi.org/10.1080/09362835.2018.1470446> |
| --- | --- |
| **Authors** | Mukhopadhyay, Sourav  Mangope, Boitumelo  Moorad, Fazlur |
| **Year** | 2019 |
| **Country/ies** | Botswana |
| **Aim** | “The purpose of this present study was to examine the experiences of inclusion by learners with SENs in Botswana’s primary schools.” […]  “The purpose of this research, therefore, was to investigate the learners’ perceptions about inclusive education and their experiences in participating in school activities.” |
| **Objectives** | N/A |
| **Research question(s)** | “The following research questions were asked:  What are the learners’ experiences in the inclusive classrooms?  What strategies should be put in place in order to accommodate all learners in the inclusive classroom?” |
| **Methodology** | “A qualitative research methodology was employed for this study and focus group discussions with learners with and without SENs were used. This particular research methodology was chosen for this study because of three main reasons. First, there has been limited research exploring views of learners with SENs at the primary level in Botswana. Second, this method offered a platform for understanding the plight of learners with SENs (Lincoln & Guba, 2000). Third, the researchers were themselves interested in hearing the voices of learners with SENs” |
| **Data collection methods** | “Two semi-structured focus group interview guides were specifically designed for this study: one for learners with SENs and the other for learners without SENs. The interview guide incorporated broad areas and issues for learners pertaining to schooling such as overall experiences, major barriers, availability of resources, infrastructure and service facilities, attitudes of peers, and their suggestions to improve the current situation of the school system. In addition a school and classroom observation guides were also designed. […] Twelve focus group discussions (six for learners with SENs and six for learners without SENs) were organized, i.e. there were two focus groups per school. Focus group discussions were carried out in Setswana (national language of Botswana) throughout the interview sessions. The researchers made sure that the composition of the group was optimally varied to gather ‘rich and thick’ data from participants. The focus group sessions lasted for one hour and thirty minutes. […] In addition, the researchers conducted both school and classroom observations. Six nonparticipant classroom observations were done to get an insight into the practice of inclusive education. During the observations, the researcher sat at the back of the classroom so that classroom activities were not affected. […] Classroom observations took between 35–40 minutes. […] After each observation, informal follow-up conversations with teachers about the lesson were held for clarity.” |
| **Data analysis methods** | “A constant-comparison method (Corbin & Strauss, 2014) was used to examine the similarities and differences in reflections across the participants. Data were entered into AtlasTi as primary documents and analyzed in a step-by-step fashion. In step one, the data from each focus group was analyzed employing line by line open-coding. In step two, each transcript was examined separately, and whenever a new theme emerged, it was highlighted. The identified themes within the transcripts were then compared across transcripts in step three. Overall themes or super-codes were then developed in step four. A summary of all transcripts was compiled in which subthemes were compared so as to come up with overall themes that were later used to report the findings of this study.” |
| **Rigour** | “This process of data analysis was carried out by the individual researchers and then compared among them to agree on the final themes. This process enhanced the reliability of the data analysis.” |
| **Stakeholder group(s)** | Primary school pupils with and without SEN |
| **Recruitment** | “Participants included 36 learners with SENs and 36 learners without SENs from six primary schools that voluntarily participated in this research. Class teachers helped in selecting both groups of participants, who were between the age group of 8–14 years. Learners with SENs are not homogeneous, given that there are various types of learners with SENs. The researchers purposively selected different categories of learners with disability to maintain this heterogeneity. […] With regard to learners without SENs, we selected those who had a peer with SENs in the same class.” |
| **Participant information** | **Total number**  74 |
|  | **Number per stakeholder group**  “36 learners with SENs and 36 learners without SENs” |
|  | **Demographics**  “between the age group of 8–14 years”  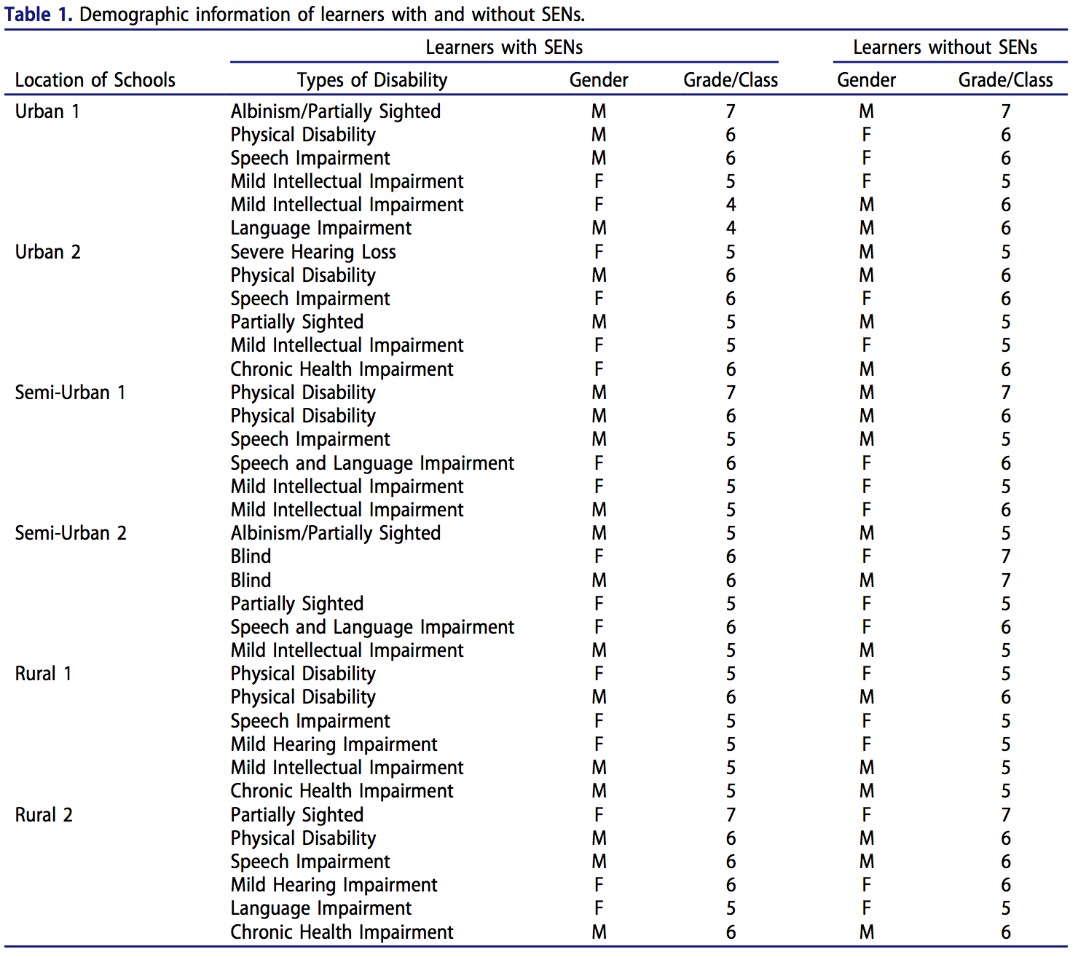 |
|  | **Other information**  N/A |
| **Relevance of DD and type** | 7/36 children with SEN (19.4%) with intellectual impairment, 9/36 (25%) with language and/or speech impairment |
| **Setting information** | **School grade and type**  “This research was carried out in six primary schools in the South Central regions of Botswana.” |
|  | **Contextual information**  N/A |
| **Themes / topics** | Accessing curriculum and instruction  Inclusive schooling  Attitudes of teachers and peers  Peer interactions |
| **Extraction method for Results and Discussion** | All extracted for analysis |
| **Quality** | Good |

### **Ngcobo & Muthukrishna (2011)**

| **Full Reference** | Ngcobo, J., & Muthukrishna, N. (2011). The geographies of inclusion of students with disabilities in an ordinary school. *South African journal of education*, *31*(3), 357-368. <https://doi.org/10.15700/saje.v31n3a541> |
| --- | --- |
| **Authors** | Ngcobo, Jabulani  Muthukrishna, Nithi |
| **Year** | 2011 |
| **Country/ies** | South Africa |
| **Aim** | “Drawing upon an in-depth case study, we use a geographic focus to unpack the visual, political and ideological dimensions of school spaces as they are experienced in the everyday lives of disabled students in an ordinary school.” |
| **Objectives** | “This paper has two broad aims. It highlights the experiential aspects of disabled children’s inclusion in an ordinary public school, and it examines the processes that maintain or challenge the spatial conditions under which inclusion and the identity of disabled children are produced.” |
| **Research question(s)** | N/A |
| **Methodology** | Qualitative (not specified) |
| **Data collection methods** | “Semi-structured interviews were held with the school principal and five teachers who had disabled learners in their classes. […] Participants were interviewed individually for about one hour. Interviews were audio-taped. Observations were done in four classrooms over three full days each in order to try to gain a sense of the nature of interactions between disabled learners, able-bodied learners and their teachers. Non-participatory observation was used with an intention to reduce any interaction with the participants and to focus the attention on the events (Burns, 2000). Observations were also conducted at three staff meetings to document the conversations teachers held about their experiences of including learners with disabilities in the school. During observations, field notes were taken and cross-checked with the teacher participants to ensure that interactions and activities were correctly recorded and interpreted. The school was willing to allow us access to its documents on condition that they remained confidential. A careful examination was made of the relevant documents, including various school policies, children’s workbooks, snap survey reports, admission forms, learners’ progress reports and departmental correspondence. Documents and artefacts were used to triangulate data elicited through other methods and to enable a rich and deep understanding of issues explored (McMillan & Schumacher, 2001). The audio-taped interviews were translated from isiZulu into English and transcribed. Transcribed interviews were validated through a process of member checking involving participants.” |
| **Data analysis methods** | “The transcriptions were analysed using a thematic analysis approach. Initial coding involved identifying broad categories of issues. Both emic and etic categories were defined (Headland, Pike & Harris, 1990). Emic categories represented insider’s views such as terms, actions and explanations that are distinct to the setting. The aim was to represent the situation from the participants’ perspective (Merton, 1972; Pike, 1967). Etic categories represented the researchers’ interpretations, concepts and explanations. The categories came from the researcher’s personal research experiences or from prevailing literature (Merton, 1972). The second phase of data analysis involved theorising the coded data in search of themes that would exemplify the dimensions of school spaces significant to the inclusion of children with disabilities. This process involved a merging of insights from literature on the notion of geographies and spatialities and inclusion/exclusion debates.” |
| **Rigour** | N/A |
| **Stakeholder group(s)** | Teachers, principal |
| **Recruitment** | N/A |
| **Participant information** | **Total number**  6 |
|  | **Number per stakeholder group**  1 school principal and five teachers |
|  | **Demographics**  N/A |
|  | **Other information**  “Two of the teachers were studying towards an honours degree in special needs education. The teachers had teaching experience ranging from six to thirty years, and they had been teaching at this school for a minimum of six years. They were all black African women teachers.” |
| **Relevance of DD and type** | 73 out of 93 students with special needs enrolled at the school (78.5%) had learning difficulties, 1 (1.1%) autism and 3 (3.23%) cerebral palsy |
| **Setting information** | **School grade and type**  “ordinary public school”  “The school offered classes from preschool to Grade 4.” |
|  | **Contextual information**  “The school is situated in a densely populated semi-rural township in the northern part of the province of KwaZulu-Natal, more than 25 km away from the nearest town. The community experiences high levels of unemployment and poverty. At the time of the study, the school had a learner population of 1,250. The age range of learners was between 3 and 17 years. The school offered classes from preschool to Grade 4. Over the years, the learner population had become very diverse. There were 95 over-age learners, who should have been at high school according to the departmental regulations for admission.  Disabled learners were admitted when the school took a conscious decision to open its door to ‘out of school’ disabled learners in the community. There were 93 learners classified as learners with special needs: 10 deaf, two epileptic, four physically disabled, three cerebral palsied, one autistic and 73 experiencing various forms of learning difficulty. These learners ranged in age from 6 years to 17 years. They had been placed in Grades 1 to 4. All learners were black African, and the majority came from the area in which the school is located. The language of learning at the school was English for hearing learners and South African Sign Language for deaf learners. The school had one teacher proficient in South African Sign Language. The home languages of learners included South African Sign Language, IsiZulu and Sesotho.” |
| **Themes / topics** | Teachers and ideological spaces  Interrogating the internal spatiality of the classroom  The policy-practice chasm |
| **Extraction method for Results and Discussion** | All extracted for analysis |
| **Quality** | Average |

### **Okyere et al. (2019a)**

| **Full Reference** | Okyere, C., Aldersey, H. M., & Lysaght, R. (2019a). The experiences of children with intellectual and developmental disabilities in inclusive schools in Accra, Ghana. *African journal of disability*, *8*(1), 1-11. <https://doi.org/10520/EJC-18caea5621> |
| --- | --- |
| **Authors** | Okyere, Christiana  Aldersey, Heather Michelle  Lysaght, Rosemary |
| **Year** | 2019 |
| **Country/ies** | Ghana |
| **Aim** | “We sought to engage children with IDD [DD] to learn about their experiences in inclusive schools.” |
| **Objectives** | N/A |
| **Research question(s)** | “What are the experiences of children with IDD in inclusive schools in Accra, Ghana?” |
| **Methodology** | “We utilised a qualitative descriptive design as described by Sandelowski (2010), which incorporates overtones or techniques of other qualitative approaches to ensure rigour (Sandelowski 2010). The qualitative descriptive approach stays close to the data and provides a straightforward, rich description and accurate account of the meanings participants ascribe to events (Neergaard et al. 2009). We used this design because it allows flexibility in utilising diverse data collection methods (i.e. observations, drawings and interviews) to derive a detailed account of participants' experiences about a phenomenon (Sandelowski 2010).” |
| **Data collection methods** | “Data were collected using structured observations, the draw-and-write technique (McWhirter 2014) and interviews. Data collection began with observations in the classroom for an average of 3 hours utilising McIntosh's (1994) observation categories for students with learning disabilities as a guide […]. Classroom observations and interviews were conducted on the school grounds. Observations included learning environments, teacher and student interactions, teaching adaptations and strategies, and participant behaviours. After each observation, field notes and memos were diarised for later transfer into Microsoft Word files at the end of each day. Writing analytic memos was instrumental in determining concepts requiring further exploration and development (Corbin & Strauss 2008). To facilitate children's participation in ways that resonated with them, we utilised the draw-and-write technique described by McWhirter (2014) as an icebreaker to solicit children's experiences. The technique entails asking children to draw a picture related to a specific topic and write about what is happening in the drawing (McWhirter 2014). Participants were invited to draw images of themselves in school and write about what is happening in the drawing. […] The results were instrumental in triggering discussions as the task was participatory and participants found it enjoyable and comfortable. We used semi-structured interviews with participants to build upon emerging themes already identified through observations and drawings and to ask new questions beyond what was observed. […] Audio-recorded interviews were conducted within 20-25 min of observations, and memos were documented at the end of each day. Writing analytic memos was instrumental to the critical thinking process and in determining concepts requiring further exploration and development (Corbin & Strauss 2008).” |
| **Data analysis methods** | “The theory that guided our study is the bioecological theory of human development, which was first proposed in the 1970s by Urie Bronfenbrenner (Bronfenbrenner & Morris 1998; Rosa & Tudge 2013 […] The theory depicts that forces at various levels - biosystem (individual child), microsystem (immediate environment), mesosystem (interactional patterns amongst two microsystems), exosystem (indirect environment), macrosystem (social values) and chronosystem (changes over a period of time) - affect the development of the child (Bronfenbrenner & Morris 1998). […] Based on its relevance to an understanding of the personal characteristics and all the contextual factors that influence the inclusion of children with disabilities, we utilised the theory to guide the organisation of the themes that emerged from the data. In this process, we mapped emergent themes onto the various levels of Bronfenbrenner's bioecological theory of human development.” […]  “We analysed data concurrently with data collection and systematically” […]  “All observations, interview transcripts, field notes and analytic memos were imported into a computerised qualitative data management software program (Nvivo 2011) to assist in the organisation of data, identification of categories and development of themes. Specifically, we shared the database and analyses of observations and interviews amongst the team. In this process, we became familiar with the data, identified and compared initial codes and grouped similar codes into categories and developed themes central to the purpose of the study.” |
| **Rigour** | “On a regular basis, the authors shared observations, drawing activities, transcribed interviews and analytic memos with each other during biweekly debriefing sessions for ideas and detailed directions for subsequent interviews. These sessions occurred throughout the data collection period and challenged the authors to think critically about the data while documenting reflections and information relevant to yielding rich data. The debriefing process was also significant to the exposure and critical evaluation of biases and positionality throughout data collection and analyses. Furthermore, the process brought different perspectives to the data and identified emerging themes that formed the basis of additional probes and checks for upcoming observations and interviews” […]  “We employed triangulation, peer debriefing, member checks and reflexivity throughout data collection and analysis to ensure trustworthiness and credibility in the study finding. We triangulated data using several data collection techniques (observations, drawings and interviews) from participants in different school settings. The first two authors engaged in peer-debriefing sessions and explored each other's views and perspectives (Pandey & Patnaik 2014). We conducted member checks wherein we consistently repeated each participant's responses during interviews to confirm their agreement prior to asking the next or follow-up questions (Pandey & Patnaik 2014). Additionally, at the end of the data collection process, we organised a closing session where we chatted with participants on emerging themes and key findings. Considering the age of the participants, we used a member-checking approach recommended by Simpson and Quigley (2016) for use with young participants. In this process, we asked participants the same questions asked in previous interviews and compared responses. All of participants' responses reflected those in previous interviews.  Reflexivity entails researchers consciously examining their biases because of previous experiences, knowledge or connections with the study population (Råheim et al. 2016). Being a Ghanaian, one of the authors approached this study as an insider with the same identity as participants and cultural knowledge of the study context. Further, this author came to this study with an enthusiasm and empathy for children with IDD demonstrated through 5 years' work for their inclusion in Ghana. Specifically, during this time, the author assisted in enrolling and relocating children with IDD into schools and half-way homes. The author's insider perspectives, experiences with the study population and the importance attached to their accessing quality education may influence the ability to ask further meaningful and insightful questions and also interpret results from a non-biased culture perspective. Subsequently, the author practised reflexivity wherein there are discussions about the knowledge and experiences of working with children with IDD with the co-authors who recommended strategies (i.e. asking questions in diverse ways, probing for further clarifications) to reduce bias. The authors engaged each other in preliminary data analysis and interpretation of the data, wherein all authors coded these same transcripts independently and came together to compare codes and identify any gaps or divergences.” |
| **Stakeholder group(s)** | Pupils with “Intellectual and developmental disabilities (IDD)” [DD] |
| **Recruitment** | “We employed a purposive sampling strategy whereby we approached participants based on specific characteristics such as age, grade, gender and number of years in an inclusive school. We recruited 16 participants from four inclusive schools in Accra, Ghana. Sampled schools were selected through the country's Special Education Ministry. We identified participants in 14 different classes in the four schools sampled. […] Participants were included in the study if (1) they provided assent and their parents' consent, (2) they had been in sampled schools for at least a year, (3) they were in the mild to moderate range of IDD and (4) after a review of their available student files including medical reports provided by school heads.” […]  “All students who met the inclusion criteria in a specific class were included in the study.” |
| **Participant information** | **Total number**  16 |
|  | **Number per stakeholder group**  16 children |
|  | **Demographics**  “A total of eight girls and eight boys participated in the study.” |
|  | **Other information**  “With the exception of one participant who lived with his father and stepmother, all participants lived with both parents” |
| **Relevance of DD and type** | Focused on inclusion for children “in the mild to moderate range of IDD” [DD] […]  “All participants met the school district's inclusive education team's criteria for IDD, which is diagnosed by a screening stage (based on child observations), an achievement test (evidence of academic achievements) and a series of tests conducted in the district assessment centre by a clinical psychologist to confirm the presence of IDD.” |
| **Setting information** | **School grade and type**  “four inclusive schools” |
|  | **Contextual information**  “district of the country's capital that has well-resourced schools compared to rural settings” |
| **Themes / topics** | “ (1) characteristics and struggles at the individual level, highlighting bio- and microsystemic factors, (2) characteristics at the environment level, highlighting macrosystemic factors, and (3) interactional patterns also highlighting microsystemic factors within the Bronfenbrenner's bioecological systemic framework.” |
| **Extraction method for Results and Discussion** | All extracted for analysis, except participant information in the Results section |
| **Quality** | Very Good |

###

### **Okyere et al. (2019b)**

| **Full Reference** | Okyere, C., Aldersey, H. M., & Lysaght, R. (2019b). The experiences of teachers of children with intellectual and developmental disabilities in inclusive schools in Accra, Ghana. *Journal of Research in Special Educational Needs*, *19*(4), 283-294. <https://doi.org/10.1111/1471-3802.12447> |
| --- | --- |
| **Authors** | Okyere, Christiana  Aldersey, Heather Michelle  Lysaght, Rosemary |
| **Year** | 2019 |
| **Country/ies** | Ghana |
| **Aim** | “an in‐depth examination of their *(teachers)* experiences involving children with IDD in classroom activities” |
| **Objectives** | N/A |
| **Research question(s)** | “This study sought to answer the question: what are the lived experiences of teachers who are delivering education to children with IDD in inclusive schools in Accra, Ghana?” |
| **Methodology** | “We followed the qualitative descriptive design as described by Sandelowski (2010) that draws strategies from other qualitative approaches to ensure rigour.” |
| **Data collection methods** | “Face‐to‐face, semi‐structured individual interviews were employed as the primary data collection method. [...] The first author interviewed participants at times that were convenient to participants during school hours each day. The face‐to‐face semi‐structured interviews were conducted in the offices of head teachers located in the school setting where only the participant and the first author were present. The interview guide focused on participants’ everyday experiences in the context of inclusion with children with IDD and supports available to assist them in the general education classroom. All interviews were audio recorded and lasted from 45 to 90 minutes.  Immediately after each interview, the first author took detailed field notes and memos using Microsoft Word. The field notes and memos incorporated participant description, interactions and non‐verbal cues (i.e. physical expressions, gestures) and were instrumental in determining follow‐up questions for further clarification and expansion. Furthermore, all field notes and analytical memos were integrated into transcripts for analysis (Crist and Tanner, 2003; Patton, 1990).” |
| **Data analysis methods** | “We employed Bronfenbrenner's bioecological theory of human development in the theoretical organisation of our findings (Bronfenbrenner and Morris, 1998). The theory posits that the interactions between environmental aspects affect the development of an individual. These environmental aspects include: biosystems (individual child) microsystems (i.e. home, school), mesosystems (i.e. interactions between home and school), exosystems (larger social systems i.e. school policies), macrosystems (socio‐cultural values) and chronosystems (adjustments over time).” […]  “In congruence with qualitative description, we utilised thematic analysis as described by Braun and Clarke due to its detailed and systematic approach to analysing qualitative data (Braun and Clarke, 2006).” […]”  “The first and second authors familiarised themselves with the data, independently identified and compared initial codes, and grouped identical codes into themes relevant to participants’ experiences.” |
| **Rigour** | “We ensured credibility in this study using five strategies: triangulation, peer debriefing, audit trails, member checks and reflexivity (Creswell and Miller, 2000; Koch, 2006; Pandey and Patnaik, 2014).” |
| **Stakeholder group(s)** | Teachers |
| **Recruitment** | “We identified 18 participants […] from 14 classrooms within four inclusive schools in Accra, Ghana who had taught children with IDD in inclusive schools for at least 1 year. We purposively selected participants based on gender, years of teaching experience and grade level taught. For example, we strategically selected teachers who had taught children with IDD in an inclusive setting for at least a year. Also, being part of a larger study that engaged children with IDD, we deliberately sought out general and special educators who taught children with IDD between grade levels two and six. […] We obtained the district and school administrative authorities’ written permission to conduct the study in the sampled schools.” […]  “Prior to the interviews, the head teachers in the sampled schools introduced the first author to participants as a researcher interested in learning about their experiences teaching children with IDD in the general education classroom” |
| **Participant information** | **Total number**  18 |
|  | **Number per stakeholder group**  “16 general educators and two special educators” |
|  | **Demographics**  “A total of 15 females and three males (representing 83% females and 17% males) participated in the study.” |
|  | **Other information**  “All participants in the study had previous experiences with students, family members or friends/acquaintances with various types of disabilities. Except for the two special educators who had attained university degrees in special education, all other participants’ highest level of education was a teacher training college certificate.”  4-40 years of teaching experience; 1-8 years of teaching experience in inclusive schools  7 (38.9%) had periodical training on disability and inclusion |
| **Relevance of DD and type** | Focused on inclusion for “Intellectual and developmental disabilities (IDD)”  “Children with IDD have ‘a group of developmental conditions characterised by significant impairment of cognitive functions, which are associated with limitations of learning, adaptive behaviour and skills’ (Salvador‐Carulla, Reed, Vaez‐Azizi, et al., 2011, p. 177).” |
| **Setting information** | **School grade and type**  four inclusive schools in Accra  “we deliberately sought out general and special educators who taught children with IDD between grade levels two and six.” |
|  | **Contextual information**  N/A |
| **Themes / topics** | “Three themes that emerged through clustering meanings: (i) experiences with the individual child (biosystems), (ii) experiences with parents of the individual child (microsystems), and (iii) experiences with supportive resources and services (microsystems and mesosystems).” |
| **Extraction method for Results and Discussion** | All extracted for analysis, except participant information in the Results section |
| **Quality** | Very Good |

### **Otukile-Mongwaketse et al. (2016)**

| **Full Reference** | Otukile‐Mongwaketse, M., Mangope, B., & Kuyini, A. B. (2016). Teachers' understandings of curriculum adaptations for learners with learning difficulties in primary schools in Botswana: issues and challenges of inclusive education. *Journal of Research in Special Educational Needs*, *16*(3), 169-177. <https://doi.org/10.1111/1471-3802.12069> |
| --- | --- |
| **Authors** | Otukile-Mongwaketse, Mpho  Mangope, Boitumelo  Kuyini, Ahmed Bawa |
| **Year** | 2016 |
| **Country/ies** | Botswana |
| **Aim** | “The aim of this study was to gain an in-depth understanding and insight into what Botswana teachers do in their classrooms or schools to meet educational needs of learners who have LD in inclusive primary school settings. It was also aimed at examining how teachers understood curriculum adaptations and how their understanding impacted on access to the curriculum by learners who have LD.” |
| **Objectives** | [See questions] |
| **Research question(s)** | “The following research questions guided the study:  What are teachers' understandings of curriculum adaptations?  What do teachers do in their classrooms to adapt the curriculum?  How does this understanding impact on access to the curriculum by learners with LD?” |
| **Methodology** | Qualitative (not specified) |
| **Data collection methods** | “The study used classroom observations and interviews to collect qualitative data. During classroom observation, the observer focused on the classroom context, the activities of teachers and students, including lesson content and instructional process, and student interactions. Teachers were observed in their classrooms for 40 minutes. These observations were intended to provide data for the purpose of describing the settings, activities, people and the meanings of what is observed from the perspective of the participants. The data also became the basis for the interviews with the teacher participants later on. Interviews were conducted in order to try to understand the issue under study from the perspective of the participants and to explore the meaning of their experiences. The interview content was based around core concepts such as inclusive education, curriculum adaptations and learners with LD using a memoire. […] All teachers interviewed were also observed in their classrooms. After each interview, participants listened to the tapes to verify points as well as ensuring accuracy and clarity (this happened outside the 15–25 minutes of interview schedule).” |
| **Data analysis methods** | “Having collected all the data from all schools, it was organised and interpreted guided by levels of coding from Creswell (2005) which are (1) open coding, (2) axial coding and (3) selective coding.  Level 1: […] Each page of transcriptions and field notes was coded in the upper right hand corner for easy identification of various sources. We then reread transcripts several times in order to get a sense of the whole data before breaking it into parts. The next stage was to write memos in the margins of the texts which were key phrases, ideas and concepts occurring in the data. […] We read and reread the data in order to develop categories and themes, and as Miles and Huberman (1994) explain, such coding allowed us to fracture the data, thereby reaching higher levels of abstraction by seeing the data in different groupings.  Level 2: The second step was to apply these codes and relationships and to compare the commonalities across all schools. This was the beginning of emergence of themes common to all schools. Thereafter, we collated the data into one dataset so that we could easily manage it from one point. This procedure enabled us to establish the relationship between categories and helped us to group them into coding families.” |
| **Rigour** | “With regard to credibility of the study, member checking was implemented. According to Erlandson et al. (1993), member checking provides for credibility by allowing members of stake‐holding groups to test categories, interpretations and conclusions. For this particular study, member checking was carried out informally by allowing participants at the end of interviews to immediately correct errors of fact or challenge interpretations. This allowed for a better understanding of the data rather than implying what participants meant and it helped in interpreting the ideas accurately. Additionally, a recoding technique was used where data were checked several times and cross‐checked to enhance possibility of new understandings. This was also carried out through discussions with research supervisors.” |
| **Stakeholder group(s)** | Teachers |
| **Recruitment** | “Two schools were purposively chosen (Burgess, 1984) from urban, semi‐urban and rural areas. The reason for choosing schools in different areas was to find out if teaching in such areas had any impact on teachers' ways of approaching curriculum and/or teaching in inclusive settings. The schools were chosen based on information provided by education officers.” […]  “The choice of teacher participants in each school was made with the advice of head teachers after they had completed consent forms. Teachers who participated were purposely chosen from different sections […] For example, each section had 2–4 classes and only one teacher was selected from a section. […] The reason for choosing participants teaching different standards was to gather balanced information on whether or not teaching a particular standard has an impact on how teachers implement inclusive education.” |
| **Participant information** | **Total number**  12 |
|  | **Number per stakeholder group**  12 teachers |
|  | **Demographics**  “The teachers in the study were in the age range of 30–48” |
|  | **Other information**  “The teachers in the study […] had mostly acquired their training in the 1980s and the early 1990s. Some participants had training in special education, others had no training in special education, even though they had some awareness of special education.” |
| **Relevance of DD and type** | “Teachers' understandings of curriculum adaptations for learners with learning difficulties” |
| **Setting information** | **School grade and type**  “The study was conducted in six primary schools in Botswana, situated in urban, semi‐urban and rural areas. All were inclusive schools with a population ranging from 150 to 950 learners.” […]  “Teachers who participated were purposely chosen from different sections: lower standard (STD 1–3), middle standard (STD 4–5) and upper standard (STD 6–7).” |
|  | **Contextual information**  “Two schools were purposively chosen (Burgess, 1984) from urban, semi‐urban and rural areas […] in the Gaborone area” |
| **Themes / topics** | 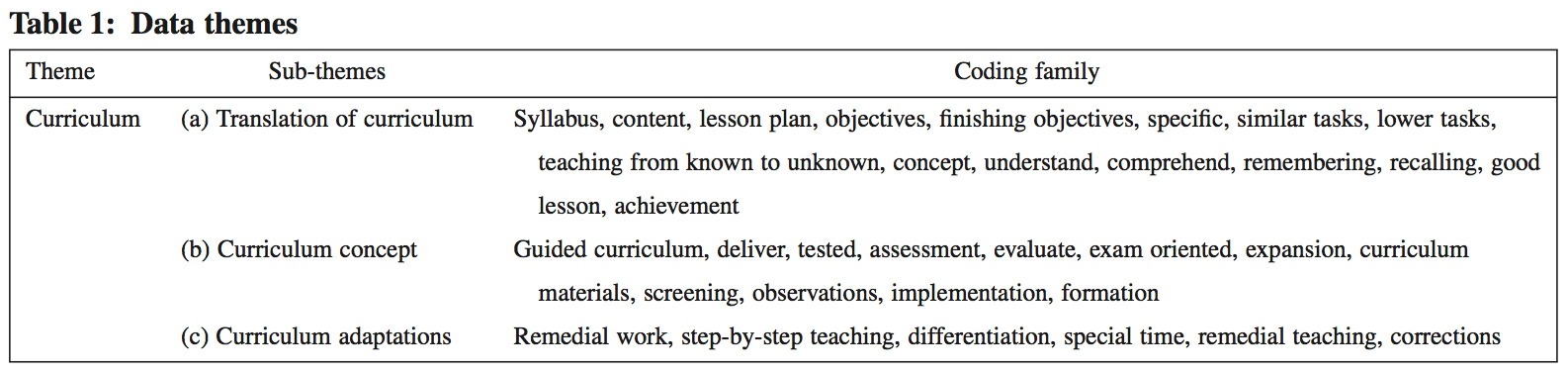 |
| **Extraction method for Results and Discussion** | All extracted for analysis |
| **Quality** | Good |

### **Potgieter-Groot et al. (2012)**

| **Full Reference** | Potgieter-Groot, L., Visser, M., & Lubbe-de Beer, C. (2012). Emotional and behavioural barriers to learning and development in the inclusive education classrooms in South Africa: Developing a training programme for teachers. *Journal of Child & Adolescent Mental Health*, *24*(1), 59-71. <https://doi.org/10.2989/17280583.2011.639775> |
| --- | --- |
| **Authors** | Potgieter-Groot, Lucia  Visser, Maretha  Lubbe-De Beer, Carien |
| **Year** | 2012 |
| **Country/ies** | South Africa |
| **Aim** | “This paper describes the development of an in-service training programme to enhance teachers’ abilities to work with learners experiencing emotional and behavioural barriers in an inclusive education environment in South Africa. The paper highlights some of the dilemmas of teachers and the strategies they found to be effective in the classroom to deal with learners experiencing barriers to learning.” |
| **Objectives** | N/A |
| **Research question(s)** | N/A |
| **Methodology** | “Action research was used to develop the programme because of its development philosophy and emphasis on the utilisation of local knowledge (Somekh and Zeichner 2009). […] The process often starts with a question on how a situation that is significant to all participants can be improved and is driven by personal commitment to contribute to human flourishing (Reason and Bradbury 2008a). Action research seeks to create participation and to engage all stakeholders as agents of transformation, rather than passive research subjects (Reason and Bradbury 2008b, Swantz 2008). In this case the wealth of knowledge and commitment in the teaching community was used to facilitate change. Action research is therefore the process of solving practical problems through participation of those involved in the situation. The researcher becomes a facilitator of this process and the participants act as co-researchers (Swantz 2008). Action research typically implies systematic cycles of action that involve evaluation, planning, action/intervention and reflection/evaluation (Armstrong and Moore 2004). “ |
| **Data collection methods** | 1. “The action research process started with a needs assessment at each of the two schools. Data were collected through four focus group discussions involving the teachers from both schools. Open-ended exploratory questions were asked about the behaviour of learners in their classes, class management strategies they use and their need for training to deal with learners with barriers to learning.” 2. “Using the results of the needs assessment and a thorough literature study on evidence-based strategies to deal with learners experiencing emotional and behaviour barriers, the initial three-session intervention was developed as in-service training for teachers. […] The researcher presented the three-session programme bi-weekly in one school and once a term in the other (as preferred by the specific school). Two external observers were present at every session to observe the intervention process and to provide feedback. After each session, the teachers had to implement what they had learned and report on these experiences in feedback forms and during the next session. […] Data were obtained from the feedback forms that the participants completed after each session and from the researcher’s and external observers’ notes made during and after each session.” |
| **Data analysis methods** | 1. “The focus group discussions were tape-recorded, transcribed and analysed using thematic analysis (Braun and Clarke 2006). The needs of teachers were identified and are included as part of the results section.” 2. “In action research data interpretation takes place throughout the research process. This enabled the researcher to make adjustments to the intervention as the needs of the educators became apparent during the process (Armstrong and Moore 2004, Holly, Arhar and Kasten 2005). […]   Qualitative data analysis was used to capture the perceptions and experiences of the participants, and to make sense of the large volume of data by restructuring and reducing the information (Miles and Huberman 1994). Data from the reports of the researcher, observers and participants were analysed separately and eventually integrated in a process of triangulation. Categories were first coded using open coding and then combined and interpreted in terms of themes. Patterns were identified from the different themes and also from the changes that occurred (Braun and Clarke 2006, Schwandt 2007). The systems theory was used as a theoretical framework (Bronfenbrenner 2005) to understand the experiences and changes that took place on different levels of the educational system. The presentation of the themes and patterns is organised in terms of the different systems levels.” |
| **Rigour** | “Emphasis was placed on the quality of data analysis. This was done by having more than one researcher who interpreted the data and using the cyclic process of action research to verify the interpretation of the experiences of teachers in their classrooms.” |
| **Stakeholder group(s)** | Teachers, school staff |
| **Recruitment** | “The research was conducted during 2008 and 2009 in two mainstream primary schools (providing education from foundational to senior phase — Grade 1 to 7) in the Johannesburg South Education District. Initially four schools were selected to participate in the research. They were purposely selected in collaboration with staff at the Johannesburg South Education District Office using two criteria: (1) schools that most needed training for teachers to deal with learners experiencing emotional and behaviour barriers, as indicated in the schools’ development plans submitted to the district office in 2008; and (2) schools that accommodate learners from a diversity of backgrounds. Purposeful sampling was used, as background information about schools was used in the selection (Babbie 2008). Two of the schools did not participate in the research: one school was not interested and the other school started but withdrew later because of non-related internal conflict.” […]  “After the principals and school governing bodies of the identified schools agreed to the research, the researcher visited the schools. Teachers were informed about the research and invited to participate voluntarily. Forty-nine teachers volunteered and gave informed consent.” |
| **Participant information** | **Total number**  49. “Forty-seven participants attended all three sessions, while two attended only the first session.” |
|  | **Number per stakeholder group**  “Most of the participants (72%) were teachers, and 28% formed part of different management structures at the schools.” |
|  | **Demographics**  “Most of the participants (81%) were females” |
|  | **Other information**  N/A |
| **Relevance of DD and type** | “The Individuals with Disabilities Act (IDEA) defines emotional and behavioural barriers as: a condition exhibiting one or more of the following characteristics over a long period of time that adversely affects a child’s educational performance: (*1) An inability to learn that cannot be explained by intellectual, sensory or health factors; (2) An inability to build and maintain satisfactory interpersonal relationships with peers and teachers; (3) Inappropriate types of behaviour or feelings under normal circumstances; (4) A general pervasive mood of unhappiness or depression; (4) A tendency to develop physical symptoms and fears associ-ated with personal or school problems* (Kavale, Forness and Mostert 2005:46, Hannell 2006:37/38).” […]  “This paper describes the development of an in-service training programme to enhance teachers’ abilities to work with learners experiencing emotional and behavioural barriers in an inclusive education environment in South Africa.” |
| **Setting information** | **School grade and type**  “The research was conducted during 2008 and 2009 in two mainstream primary schools (providing education from foundational to senior phase — Grade 1 to 7) in the Johannesburg South Education District.” |
|  | **Contextual information**  “(1) schools that most needed training for teachers to deal with learners experiencing emotional and behaviour barriers, as indicated in the schools’ development plans submitted to the district office in 2008; and (2) schools that accommodate learners from a diversity of backgrounds.” […]  “Of the two schools that participated in the research, one is situated in a low socio-economic community and also provides education to learners from a nearby informal settlement (very low socio-economic community). Learners who attend this school grow up in severe poverty as a consequence of high unemployment levels. The second school is situated in the southern suburbs of Johannesburg in a middle class environment. About 70% of the learners from this school come from a middle class background, while 30% are either immigrants from other African countries or children who commute from the lower socio-economic neighbouring communities in Soweto. In both school communities social ills like drug abuse and family disintegration are high." |
| **Themes / topics** | Needs analysis (learner behaviour, teacher strategies, training needs)  Feedback after implementation of the intervention (new knowledge gained, change in teachers’ perspectives, in learners behaviour, in the school environment, in the relationship between the school and parents, in the education system) |
| **Extraction method for Results and Discussion** | All extracted for analysis |
| **Quality** | Very Good |

### **Seabi (2010)**

| **Full Reference** | Seabi, J. (2010). Foundation phase educators' perceptions of attention deficit hyperactivity disorder at a mainstream primary school. *South African Journal of Higher Education*, *24*(4), 616-629. <https://doi.org/10520/EJC37627> |
| --- | --- |
| **Authors** | Seabi Joseph |
| **Year** | 2010 |
| **Country/ies** | South Africa |
| **Aim** | “This study intended to examine educators’ perceptions surrounding ADHD. This included their thoughts on the incidence rate, causes and possible interventions for children presenting with ADHD.” |
| **Objectives** | N/A |
| **Research question(s)** | N/A |
| **Methodology** | Qualitative |
| **Data collection methods** | “Semi-structured interviews were conducted by the researcher. In the interviews the researcher asked for more clarity or detail on specific responses so that a comprehensive understanding may be achieved. The interview schedule was piloted on two educators from the same phase, and from a similar school, as the sample.” |
| **Data analysis methods** | “The main categories and themes that emerged were identified using the process of thematic content analysis. Thematic content analysis consists of ‘burrowing through written records in order to discover their characteristics’ (Rosnow and Rosenthal 1996, 81). In this study the categories for analyses were based on the aims and research questions of the study, while the themes were based on the pattern of responses that emerged. Qualitative data was gained from the semi-structured interviews and was analyzed according to the pre-set categories and the emerging themes. The frequency of these themes was then analyzed quantitatively to support the qualitative data.” |
| **Rigour** | N/A |
| **Stakeholder group(s)** | Teachers |
| **Recruitment** | “The six foundation educators from the school were invited to participate in the study. Of the six educators, five indicated a willingness to participate in the study by giving written informed consent. Arrangements were then made to conduct the individual interviews at a time that was suitable for the educators.” |
| **Participant information** | **Total number**  5 |
|  | **Number per stakeholder group**  5 teachers |
|  | **Demographics**  N/A |
|  | **Other information**  N/A |
| **Relevance of DD and type** | Focus on ADHD |
| **Setting information** | **School grade and type**  Mainstream  Foundation |
|  | **Contextual information**  “The research was conducted in a private mainstream Christian school. The school comprises of approximately 280 learners and does accommodate learners from all races (White, Coloured, Black, Indian, and Asian). However the majority of the learners at the school are white. Of the fifteen permanent teaching staff, two are coloured and the other thirteen are white. It is interesting to note that there is one male educator on staff, the principal.” |
| **Themes / topics** | “The results have been arranged into four distinctive categories based on the aims of the study, namely, educators’ understanding of ADHD, perceptions on the causes of ADHD, intervention strategies and incidence rate.” |
| **Extraction method for Results and Discussion** | All extracted for analysis |
| **Quality** | Poor |

### **Uba & Nwoga (2016)**

| **Full Reference** | Uba, C. D., & Nwoga, K. A. (2016). Understanding stigma from a sociocultural context: mothers’ experience of stigma directed towards children with special educational needs. *International journal of inclusive education*, *20*(9), 975-994. <https://doi.org/10.1080/13603116.2016.1145259> |
| --- | --- |
| **Authors** | Uba, Chijioke Dike  Nwoga, Kechinyerem Amaka |
| **Year** | 2016 |
| **Country/ies** | Nigeria |
| **Aim** | “The aim of the study was to explore how parents’ interpretation of stigma directed towards their children with SEN affects the educational decision they make on behalf of these children.” |
| **Objectives** | “Specifically, it explores mothers’ experience of stigma directed at their children with SEN and the coping strategies they adopt to negotiate the pressures and stress arising from their stigma experience. It also explores how their interpretations of this stigma experience may underpin the educational choices and decision made on behalf of their children.” |
| **Research question(s)** | N/A |
| **Methodology** | “Focusing on the individual's narratives is a good way of uncovering the individual's claims about self and identities (Orbuch 1997; Bamberg 2004). Narratives are individuals’ self-explicated accounts of connected events and/or the point of view individuals adopt in representing selves and negotiating identities (Bamberg 2004). […] Thus, narrative inquiry is considered relevant in uncovering mothers’ accounts and interpretation of their (courtesy) stigma experience, the mechanisms they adopt (e.g. countering master narratives on stigma) in coping with their stigma experience and how these may determine their representation of selves, experiences and decisions.” |
| **Data collection methods** | “Since the study's main objective is to explore the mothers’ interpretation of the stigma experienced by their SEN children and how this affects choice made for their children, narrative interviews were considered appropriate for our study. Narrative interviews are useful when the researcher, as in our case, seeks to gain insight into how respondents make sense of their everyday lives in relation to the research objectives (Bates 2005).” […]  “A total of eight in-depth interviews were conducted” […]  “The interviews started with a clarification of the stigma concept (in line with the explication of Link and Phelan, 2001) to ensure that all respondents understood the concept and that data collected were consistent across board. Consistent with the study's objectives, mothers were asked direct questions that centre on their experiences and interpretation of stigma directed towards their children with SEN. Following from this, they were asked questions that required them to account for how the stigma experience affects the education-related decisions (for instance, to send or not send their children to school) made on behalf of their children. […] The interviews lasted between 45 minutes and 1 hour. This time frame has been used in a significant amount of studies and is assumed sufficient for discussions that will yield in-depth data (Massey 2010).” |
| **Data analysis methods** | “Bamberg's (2004) idea of ‘positioning’ was combined with thematic analysis at semantic and latent levels (Braun and Clarke 2006) in the analysis of the interview data. First, we draw from Bamberg's (2004) ideas of positioning to analyse data at two levels. The first level corresponds with analysis at the semantic level (Braun and Clarke 2006). Here, the focus is on the analysis of narrative content, that is, respondents’ accounts and responses to the interview questions and how these are consistent or inconsistent with the dominant societal perspectives (master narratives) about stigma. The second level focuses on establishing how respondents ‘position themselves in relation to discourses by which they are positioned’ (Bamberg 2004, 367). This is aligned to thematic analysis at the latent level. At the latent level, a deeper interpretative approach is employed. This approach goes above the semantic, in that it is concerned with uncovering the underlying ideas, assumptions and conceptualisations that are theorised as shaping or informing the semantic content of the data. Specifically, analysis at this level allowed us to uncover how respondents own narratives are employed to negotiate the dominant worldviews or master narratives (sometimes by countering them) about stigma in their bid to (re)position self and (re)present identities in line with their situation as mothers of SEN children.” |
| **Rigour** | N/A |
| **Stakeholder group(s)** | Parents. “The study focuses extensively on the experiences and narratives of mother of children with SEN. […] The role of mothers as the prominent childcare provider in the family qualifies them as key stakeholder in the overall education of the child. This prominent role makes them an interesting and relevant category of respondents for studies aiming to explore aspects of their children's education.” |
| **Recruitment** | “Initial telephone contact with three private and three public schools was made by the researchers from UK. Subsequently, one of the researchers visited the schools to arrange interviews with mothers. The researcher was advised by the school head teachers to approach mothers as they dropped off their children to the school in the morning. Initial response rate was poor. None of the mothers was willing to grant interviews in three of the schools (one public and two private) initially visited. However, three respondents agreed to be interviewed in a different International School where one of the researchers had previously worked. We attribute this favourable response rate to the sensitive nature of the topic whereby respondents’ familiarity with this researcher played a part in their recruitment. Given the sensitive nature of the topic and the problem of recruiting respondents, a snowballing method was then applied. The first set of mothers who were recruited were requested to recommend other mothers of SEN children from any other schools to participate in the study. The researcher then contacted the snowballed mothers from other schools and arranged interview dates and time over the phone. Interestingly, one of the mothers who turned down the initial interview request when she was met by the researcher at her child's school later agreed to be interviewed when contacted by one of the initially recruited mothers.” […]  “The principle of data saturation (Clisett 2008) was applied to ascertain if there was need for further interviews. Consistent with this principle, data collection was stopped when nothing new was being discovered after the eighth interview.” |
| **Participant information** | **Total number**  “A total of eight in-depth interviews were conducted with mothers of learners with SEN” |
|  | **Number per stakeholder group**  8 mothers |
|  | **Demographics**  “The age range of respondents is 35–54 while the age range of their children is 6–15.” |
|  | **Other information**  “Save for one child who was supposedly home-schooled, all other children attended either private primary/secondary or public primary/secondary schools. Respondents were evenly split along the line of their marital status (four married and four single).”  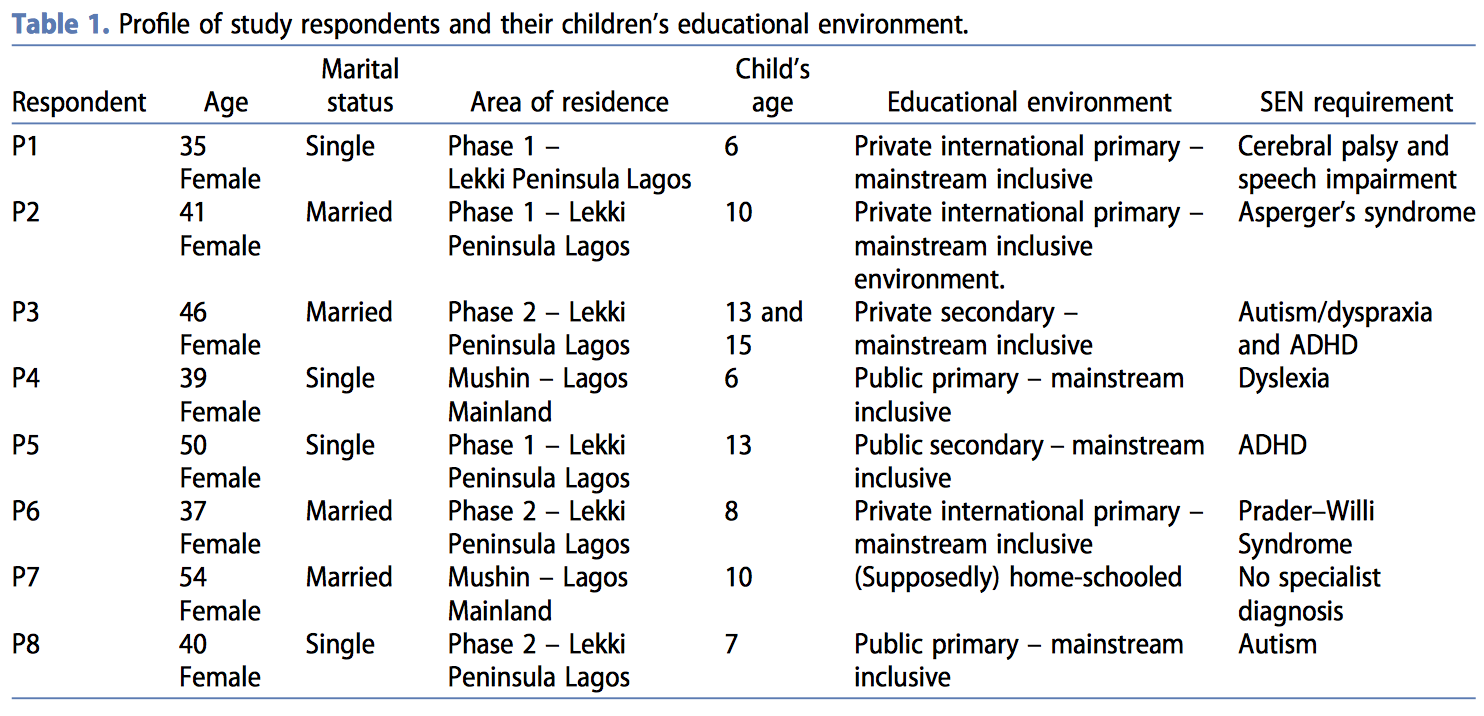  “The mothers interviewed at their workplaces were top managers in different organisations and resided in Lekki Peninsular. The rest were traders (two) and an unemployed housewife, one trader resident in Lekki and the other two in Mushin, Lagos” |
| **Relevance of DD and type** | The children with SEN of 4 out of 8 parents (50%) have ASD and/or ADHD; 2 children (25%) had a syndrome associated with intellectual disabilities and speech disorders (cerebral palsy and Prader-Willi syndrome) and only 2 (25%) had another disability or unknown |
| **Setting information** | **School grade and type**  three different International Primary Schools and a public primary/secondary school in Lagos. |
|  | **Contextual information**  “The majority of respondents (six) were currently residing in estates or duplexes in the Lekki Peninsular area of Lagos. This area is the fastest real estate market in the whole of Africa and monthly rents (approximately 500–700 USD as at 2012) are consistently on the increase (Iroham et al. 2014). The average rental price is significantly higher than the average monthly income (480–645 USD) of middle-income earners in the metropolis (see Robertson, Ndebele, and Mhango 2011). According to Babawale and Olusola (2012, 7691), ‘house prices and rents represent some of the highest in the metropolis and are, in some cases, denominated in dollars’. In addition, ‘residents of this area share similar locational characteristics and income groups’.” […]  “The interviews were conducted at respondents’ choice locations and time. Five interviews were conducted at respondents’ workplace, one in the market place and two at the respondents’ residence.” |
| **Themes / topics** | “The study findings are presented and discussed under four themes: the social construction of stigma in Nigeria, stigma experiences within the school environment, mothers’ interpretation of their child's stigma experience and the coping mechanisms (different ways of dealing with stigmatisation of children with special needs) adopted in dealing with stigma stress.” |
| **Extraction method for Results and Discussion** | Theme 1, on “The social constructions of stigma in Nigeria” in the community, rather than in education, and theme 2, "Mothers’ awareness and interpretation of their stigma experience", are not extracted for analysis. Themes 1 and 4 are extracted for analysis. Sections of theme 4 which are not relevant to education will not be considered for analysis. |
| **Quality** | Good |

### **Van Schalkwyk & Marais (2017)**

| **Full Reference** | Van Schalkwyk, I., & Marais, S. (2017). Educators’ relational experiences with learners identified with fetal alcohol spectrum disorder. *South African Journal of Education*, *37*(3), 1-9. <https://doi.org/10520/EJC-9b4a69aa3> |
| --- | --- |
| **Authors** | Van Schalkwyk, Izanette  Marais, Sandra |
| **Year** | 2017 |
| **Country/ies** | South Africa |
| **Aim** | “This study sought to attain an in-depth understanding of a group of South African educators’ relational experiences with FASD learners in a rural area” |
| **Objectives** | N/A |
| **Research question(s)** | “What is the nature of educators’ relational experiences with FASD learners?” |
| **Methodology** | “In collecting data, a qualitative research approach was chosen, as this approach creates a deeper understanding of the meaning of experiences (Rubin & Babbie, 2014).” |
| **Data collection methods** | “Data was firstly gathered via individual semi-structured interviews, as the main purpose of these person-to-person conversations is to access specialised information (Merriam & Tisdell, 2016:108). During the personal interviews participants responded to five open-ended requests […] Next, focus groups were conducted […] This form of group interaction allowed the educators to comment on each other’s experiences and points of view. The same participants who took part in the individual interviews also participated in two focus groups (seven participants per focus group). The focus group discussions of approximately 90 minutes each were held with the participants. Questions used for the focus group interviews were linked to the information obtained during the personal interviews.” |
| **Data analysis methods** | “The study was conducted from a positive psychology approach, because South African researchers working within this theoretical framework, placing emphasise on the importance of relational, personal and collective wellbeing (Geldenhuys, 2016; Prilleltensky, 2012).” […]  “The qualitative data collected via semi-structured interviews and focus groups, formed the ‘text’ for analysis. Using 14 participants allowed for data saturation and diverse perspectives. As an interpretation trustworthiness check, interpretations were checked with the individuals who were interviewed (Merriam & Tisdell, 2016). A thematic analysis, as described by Braun and Clarke (2013), was conducted, which involved repeatedly going through the entire data set in order to discern patterns of meanings. This involved six steps: becoming familiar with the data; generating initial codes; searching for themes; reviewing themes; defining and naming themes; and producing the report.” |
| **Rigour** | “Collecting data in two phases using different methods ensured the richness of the data and credibility of the information gathered (Ellingson, 2009; Tracy, 2010).” […]  “As an interpretation trustworthiness check, interpretations were checked with the individuals who were interviewed (Merriam & Tisdell, 2016).” |
| **Stakeholder group(s)** | Teachers |
| **Recruitment** | “Purposive sampling was employed to intentionally select participants with specific features (Strydom, 2011). For the purpose of the current study, fourteen participants were invited, who are educators with the needed experience of working with FASD learners, since they were regarded as holders of the data necessary for the study (Maree & Van der Westhuizen, 2009). Inclusion criteria entailed that these participants were educators at one primary school in the Cape Winelands District of South Africa. The particular school is situated in a rural area where the incidence of FASD is extremely high.” […]  “This study is limited to one primary school community located in a particular South African community, with a high prevalence of alcohol-affected children. The selection of participants could also have been biased because the educators were selected by the principal of the school.” […] “Issues regarding age and gender did not exclude any participants.” |
| **Participant information** | **Total number**  14 |
|  | **Number per stakeholder group**  14 teachers |
|  | **Demographics**  The sample comprised of 11 female and three male educators. |
|  | **Other information**  N/A |
| **Relevance of DD and type** | Focused on inclusion for FASD.  “The clinical diagnosis of FASD learners was conducted by FASER (May et al., 2013).” […]  “The sample was drawn from educators who teach in classes with presumed FASD learners in the selected primary school.” |
| **Setting information** | **School grade and type**  “Inclusion criteria entailed that these participants were educators at one primary school in the Cape Winelands District of South Africa.” |
|  | **Contextual information**  “A rural area where the incidence of FASD is extremely high.” |
| **Themes / topics** | Theme 1: Relational Qualities of Educators’ Experiences with FASD Learners Theme 2: Risks Associated with Educators’ Personal Resources, Functioning and Job Satisfaction  Theme 3: Educators’ Unique Competencies and Challenges with FASD Learners |
| **Extraction method for Results and Discussion** | All extracted for analysis |
| **Quality** | Good |

### **Walton & Rusznyak (2014)**

| **Full Reference** | Walton, E., & Rusznyak, L. (2014). Affordances and limitations of a special school practicum as a means to prepare pre-service teachers for inclusive education. *International Journal of Inclusive Education*, *18*(9), 957-974. <https://doi.org/10.1080/13603116.2013.872203> |
| --- | --- |
| **Authors** | Walton, Elizabeth  Rusznyak, Lee |
| **Year** | 2014 |
| **Country/ies** | South Africa |
| **Aim** | “This article contributes to this body of knowledge by reporting on a practice-based study concerned to understand the extent to which practicum placements in special schools can contribute to pre-service teacher learning for inclusive education.” […]  “To explore pre-service teachers’ expectations, experiences and what they report learning from practicum placements in special schools” |
| **Objectives** | “During the first set of interviews (before the practicum in special schools),we explored participants’ reasons for requesting a special school placement and their expectations of the practicum. The second set of interview questions (after the pre-service teachers completed their special school practicum) asked participants to reflect on their experiences and learning in during their time observing and teaching in a special school. The third set of interviews (after a subsequent practicum in a main-stream school) sought to investigate how, if at all, pre-service teachers believed their learning or experience in the special school practicum had impacted or influenced their teaching in a mainstream school.” |
| **Research question(s)** | “With inclusive education being explicitly and implicitly taught in coursework throughout our B.Ed. programme, we are interested in discovering the extent to which learning for inclusive education is possible in the context of a special school practicum” |
| **Methodology** | “We designed a qualitative study within an interpretive tradition (Merriam 2009) to explore pre-service teachers’ expectations, experiences and what they report learning from practicum placements in special schools” |
| **Data collection methods** | “This article reflects our analysis of the data obtained from three sets of focus group interviews. The first were held with the participants before the practicum in the special school, the second were immediately on their return to the university and the third were nine months later after they had completed another practicum in a fairly well resourced and functional public school. By the third focus group interviews, they had completed the coursework component in inclusive education and so had had the opportunity to use the theory of the coursework to reflect on their experience of practice. We draw primarily on what pre-service teachers revealed about their learning from the special schools in the third and final focus group interviews.” […]  “The three sets of focus group interviews were convened at the convenience of the participants and we used semi-structured and open-ended question.” […]  “The value of focus group interviews is its time efficiency and its potential to generate rich data as participants build on one another’s ideas as they contribute to a discussion (Stewart, Shamdasani, and Rook2007; Hesse-Biber and Leavy2011).” |
| **Data analysis methods** | “The focus group interviews were audio recorded and transcribed for accuracy and to enable us to make extensive use of participants’ own words. The transcriptions were our primary data, which we subjected to an explicitly deductive analysis (Mason 2002) in that we borrowed categories from the seven areas that Loreman (2010a) regarded as essential for pre-service teacher education for inclusion. We sorted the data into these categories (Merriam 2009).” |
| **Rigour** | “We recognise the potential for participants to dominate the discussion in ways that may make one participant’s perspective influence others. We therefore took care in the presentation of our findings to include examples of students’ quotes only where that perspective was shared by participants in at least two of the focus group interviews held. We also recognise the potential for bias because of the unequal power relations between the participants and us as both teacher educators and researchers. To reduce the impact of these unequal power relations, we presented ourselves to the participants as researchers who wanted to learn from them about their perspectives and their experiences with the aim of strengthening the practicum experiences for future cohorts of pre-service teachers. We are also mindful that participating in an interview may increase pressure on participants to offer socially acceptable, rather than honest responses (Nederhof1985; Fisher1993). While this potential for bias could not be completely eliminated from the study, we sought to reduce its impact by keeping the focus groups small and in guaranteeing participants of their anonymity in the publication of the research.” […]  “To enhance the trustworthiness and credibility of our findings, we provide details below about participants and the placement contexts to enable readers to consider the extent to which findings are transferable” |
| **Stakeholder group(s)** | Student teachers |
| **Recruitment** | “Having secured approval for our study from the university ethics committee, we invited the 19 pre-service teachers who had requested a practicum placement within a special school in their third year of study to participate in the study. Fifteen pre-service teachers agreed to participate” |
| **Participant information** | **Total number**  15 |
|  | **Number per stakeholder group**  15 student teachers |
|  | **Demographics**  “Fifteen pre-service teachers agreed to participate, 14 of whom were women. The participants represented diverse race groups” |
|  | **Other information**  N/A |
| **Relevance of DD and type** | 9 participants (60%): attended practicum in school 1 (learning difficulties and other)  1 participant (6.7%): attended practicum in school 2 (non-specified other support needs)  5 participants (33.3%): attended practicum in school 1 (cognitive disabilities and other) |
| **Setting information** | **School grade and type**  “All but one were specialising in primary and junior high school (grades 4–9) teaching”  Special school praticum |
|  | **Contextual information**  “Our university, situated in Johannesburg, offers a four year pre-service teacher education qualification – a Bachelor of Education (B.Ed.). Pre-service teachers are taught the principles of inclusive education through a hybrid of an infusion approach with dedicated coursework within a compulsory third-year education theory course called ‘Diversity, Inclusion and Pedagogy’ […] In each of the four years, pre-service teachers undertake two practicum sessions which are supervised and assessed by university teacher educators. In their first, second and fourth years, pre-service teachers complete a practicum in mainstream schools in and around Johannesburg. In their third year, however, pre-service teachers who have shown acceptable levels of classroom competence are encouraged to undertake a practicum placement in a ‘diverse’ context or in a school with ‘diverse’ students. In these cases, supervision is provided by teachers in the schools, and pre-service teachers are required to complete a number of reflective and analytical tasks in addition to their regular lesson preparation, all of which are submitted for evaluation on their return to the university.”  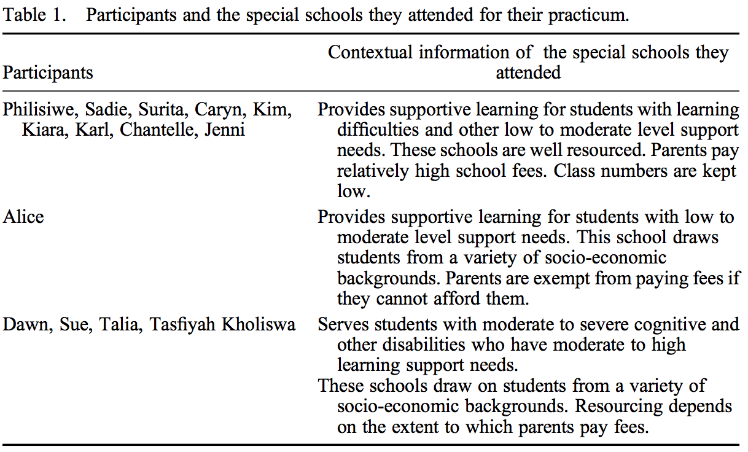 |
| **Themes / topics** | Understandings of and attitudes to diversity and inclusion  Inclusive instructional planning  Instructing in ways conducive to inclusion  Collaboration with stakeholders  Engaging in meaningful assessment, lifelong learning, and fostering a productive social climate  Critical sensibilities |
| **Extraction method for Results and Discussion** | All extracted for analysis |
| **Quality** | Excellent |

### **Yoro et al. (2020)**

| **Full Reference** | Yoro, A. J., Fourie, J. V., & van der Merwe, M. (2020). Learning support strategies for learners with neurodevelopmental disorders: Perspectives of recently qualified teachers. *African Journal of Disability (Online)*, *9*, 1-10. <https://doi.org/10.4102/ajod.v9i0.561> |
| --- | --- |
| **Authors** | Yoro, Amarachi J  Fourie, Jean V.  van der Merwe, Martyn |
| **Year** | 2020 |
| **Country/ies** | South Africa |
| **Aim** | “Given the potential challenges newly qualified teachers may experience when teaching learners presenting with NDD, this study explored the understanding and experience of recently qualified teachers as well as the support strategies applied in accommodating and supporting learners with NDDs in mainstream classrooms in their first year of teaching.” |
| **Objectives** | N/A |
| **Research question(s)** | N/A |
| **Methodology** | “Using an interpretative, generic qualitative design (Merriam 2009)” |
| **Data collection methods** | “Data were collected over a period of 6 months.” […]  “Data were collected using three different methods: semi-structured interviews, observations and critical incident reports. Each of the interviews was carried out individually and lasted for about 45–60 min. The interview guide consisted of open-ended questions focusing on three broad themes. The first theme elicited the participants’ knowledge and understanding of NDDs. The nature and kind of NDDs was the focus of the second theme. The third theme described the support strategies used by the teachers in their classrooms to support learners with NDD. The questions asked were direct and flexible, which ensured credibility in interviewing participants (Babbie & Mouton 2007). One of the major advantages of semi-structured interviews is the comprehensiveness, detail and depth of information generated from the participants (Creswell & Poth 2018). The observation was used in collecting data as it is a major technique that offers a first-hand account of the study situation (Merriam 2009). Four out of the six participants were observed, and all observations were done within a 45-min class period. The observations were guided by a checklist that focused on the teacher’s experience, kinds of NDDs in the classroom, support strategies and other extra notes. […] The final phase of data collection was done in the absence of the researcher as participants were given a critical incident report document to complete to enable them to record any incident that occurred with learners exhibiting NDD in the absence of the researcher.” |
| **Data analysis methods** | “Six steps of thematic content analysis were applied in analysing the raw data to generate themes (Braun & Clarke 2006). [the authors then described the six steps in detail]” |
| **Rigour** | “Trustworthiness is a way of ensuring thoroughness in qualitative research without Losing its relevance (Mahlo 2011). The principles of trustworthiness were adhered to throughout the research using informed consent, checking and confirming transcribed data with participants, and clarity of methods used for data collection.” |
| **Stakeholder group(s)** | Teachers |
| **Recruitment** | “Using an interpretative, generic qualitative design (Merriam 2009) newly qualified teachers from six mainstream, secondary schools in Gauteng province of South Africa were purposefully invited to participate in the study.” […]  “Written informed consent was obtained from the participants and pseudonyms were used to ensure their anonymity.” |
| **Participant information** | **Total number**  6 |
|  | **Number per stakeholder group**  6 teachers |
|  | **Demographics**  “The participants in this study consisted of five women and one man. The participants (aged 22–28 years) were representative of each racial group” |
|  | **Other information**  “Newly qualified teachers. These teachers had recently completed their Postgraduate Certificate in Education and were teaching in inclusive mainstream classroom” […]  “The participants […] had been teaching in the mainstream classroom for over 8 months.” |
| **Relevance of DD and type** | Inclusion of children with DD (mostly ADHD) |
| **Setting information** | **School grade and type**  six mainstream, secondary schools |
|  | **Contextual information**  “Inclusive mainstream classrooms, where some of the learners manifested with NDDs such as specific learning disability (SLD) and ADHD.” […]  “Three schools were located in an urban area serving a high socio-economic class, and the other three schools were in a township area serving low socio-economic class. These schools were purposefully selected because they had learners with NDD in their classrooms with teachers who were recently qualified.” |
| **Themes / topics** | Theme 1: Teachers’ understanding of neurodevelopmental disorders  Theme 2: Teachers’ experiences of the different types of neurodevelopmental disorders (ADHD and specific learning disorders)  Theme 3: Support strategies |
| **Extraction method for Results and Discussion** | The section exclusively relative to specific learning disorders not extracted. Everything else extracted for analysis. |
| **Quality** | Good |

### **Yssel et al. (2007)**

| **Full Reference** | Yssel, N., Engelbrecht, P., Oswald, M. M., Eloff, I., & Swart, E. (2007). Views of inclusion: A comparative study of parents' perceptions in South Africa and the United States. *Remedial and Special Education*, *28*(6), 356-365. <https://doi.org/10.1177/07419325070280060501> |
| --- | --- |
| **Authors** | Yssel, Nina  Engelbrecht, Petra  Oswald, Marietjie Magdalena  Eloff, Irma  Swart, Estelle |
| **Year** | 2007 |
| **Country/ies** | South Africa (76.2%) and United States |
| **Aim** | “The purpose of this collaborative research study by researchers at three South African universities and a midwestern university in the United States was to compare the perceptions of parents regarding inclusion across three geographical and cultural regions.” |
| **Objectives** | N/A |
| **Research question(s)** | “Do different political, cultural, and ethnic backgrounds affect parents’ expectations and experiences of their children’s education? Finally, what can we learn from the voices of parents globally to develop a successful home–school partnership that will benefit all children’s education?” |
| **Methodology** | Qualitative (not specified) |
| **Data collection methods** | “South African parents from urban school districts in the Western Cape and Gauteng provinces participated in six focus group discussions. The two focus group discussions in the United States were conducted in two school districts (one urban, the other including urban and rural schools) in different parts of a Midwestern state. [..] The leading question at each interview, ‘Tell me about your experiences regarding your child’s inclusion’ was followed by specific questions about the placement process and the choices that parents made regarding placement, acceptance by general education peers, and the nature of their collaboration with teachers. All focus group interviews were facilitated by the researchers—two at each discussion in South Africa, one researcher at both focus group discussions in the United States. The researchers also transcribed the audiotaped interviews. Focus group interviews lasted approximately 90 min each, and facilitators ensured that each parent participated” |
| **Data analysis methods** | “We analyzed the data for themes using the constant comparative method as developed by Glaser and Strauss (1967, as cited in Merriam, 1998; Morse & Richards, 2002). The constant comparative method consists of a simultaneous process of inductive category coding and a comparison of all units of meaning. The focus of this analysis was on perceptions of parents regarding the inclusion of their children with disabilities. The units of meaning were compared with each other and subsequently grouped with similar units of meaning (i.e., categorized). New categories were formed as new units of meaning emerged. South African researchers analyzed their data during a 2-day work session attended by all four members of the team; their American colleague analyzed the data from the two focus groups independently and sent the raw data and the analysis to the South African collaborators.” |
| **Rigour** | “Data collected by different researchers may have resulted in nuanced differences, especially because the questions were open ended. However, the data were examined collectively, and every attempt was made to ensure interrater reliability during the process.” |
| **Stakeholder group(s)** | Parents: “Parents of children with disabilities in inclusive education” |
| **Recruitment** | “Principals, district personnel, and support groups identified the participants in South Africa; in the United States, special education directors identified the parents. In both countries, parents were selected who were informed about inclusion and who would be willing to share information about their experiences. We invited parents to contact us if they were interested in participating and explained the purpose and nature of the project. Parents were assured that participation was voluntary, and they signed consent forms.” |
| **Participant information** | **Total number**  42 (32 SSA) |
|  | **Number per stakeholder group**  42 parents (32 SSA) |
|  | **Demographics**  “In South Africa, volunteers were 32 parents (7 fathers  and 25 mothers); in the United States, we had 10 parents (5 mothers from each school district).” |
|  | **Other information**  “In all cases, we interviewed parents of children who were either fully included in general education classes or were in self-contained settings in general education schools and were included to a lesser degree in some classes.”  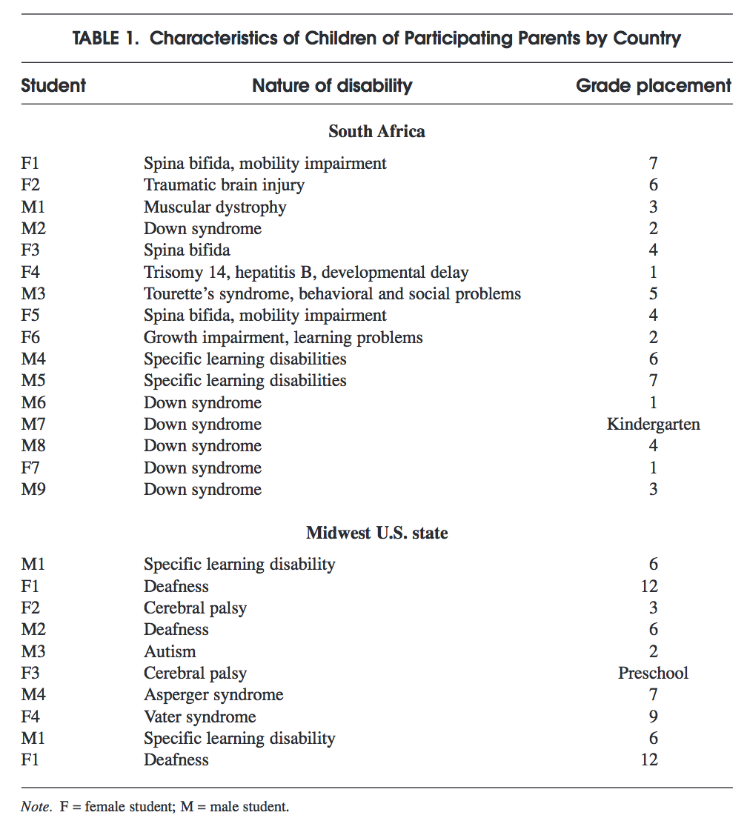 |
| **Relevance of DD and type** | In SA: 6/16 (37.5%) Down syndrome, 1/16 (6.3%) Tourette syndrome, 2/16 (12.5%) other generic learning problems or developmental delay  In US: 2 cerebral palsy (20%), 2 (20%) ASD  Total: 50% DD |
| **Setting information** | **School grade and type**  In SA: 1 (6.3%) Kindergarten, 15 (93.7%) grades 1-7 (primary and secondary)  In US: 1 (10%) Preschool, 9 (90%) grades 2-12 (primary and secondary) |
|  | **Contextual information**  In South Africa, volunteers were 32 parents (7 fathers  and 25 mothers); in the United States, we had 10 parents (5 mothers from each school district). |
| **Themes / topics** | “The major themes in this comparative study include parents’ rights, parental advocacy, social aspects, placement decisions, resilience, general education teachers, general education stu- dents’ acceptance, and having a child with a disability..” |
| **Extraction method for Results and Discussion** | All extracted for analysis. Discussions of findings limited to the US will be ignored in the analysis. |
| **Quality** | Poor |
