# Supplementary material for: Stakeholder experiences, attitudes and perspectives on inclusive education for children with developmental disabilities in sub-Saharan Africa: A systematic review of qualitative studies
Source: Autism. 2022 May 30;26(7):1606–25. doi: 10.1177/13623613221096208 (PMC9483198; doi:10.1177/13623613221096208)
Supplement: sj-docx-4-aut-10.1177_13623613221096208 – Supplemental material for Stakeholder experiences, attitudes and perspectives on inclusive education for children with developmental disabilities in sub-Saharan Africa: A systematic review of qualitative studies [file sj-docx-4-aut-10.1177_13623613221096208.docx]

## Supplementary Material D: Summary of compiled Critical Appraisal Skills Programme (CASP) checklists

| **Study** | **Was there a clear statement of the aims of the research?** | **Is a qualitative methodology appropriate?** | **Was the research design appropriate to address the aims of the research?** | **Was the recruitment strategy appropriate to**  **the aims of the research?** | **Was the data collected in a way that addressed the**  **research issue?** | **Has the relationship between researcher and**  **participants been adequately considered?** | **Have ethical issues been taken into consideration?** | **Was the data analysis sufficiently rigorous?** | **Is there a clear statement of findings?** | **Quality judgement** |
| --- | --- | --- | --- | --- | --- | --- | --- | --- | --- | --- |
| Alhassan (2017) | Yes | Yes | Yes | Can’t tell | Yes | No | Can’t tell | Can’t tell | Yes | Average |
| Bannink (2016) | Yes | Yes | Yes | Yes | Yes | No | Yes | Can’t tell | Yes | Good |
| Bannink (2020) | Yes | Yes | Yes | Yes | Yes | No | Yes | Yes | Yes | Very Good |
| Bannink (2020) | Yes | Yes | Yes | Yes | Yes | Yes | Yes | Yes | Yes | Excellent |
| Brydges (2020) | Yes | Yes | Yes | Yes | Yes | No | Yes | Can’t tell | Yes | Good |
| de Jager (2011) | Yes | Yes | Yes | Yes | Can’t tell | No | Yes | No | Yes | Average |
| de Jager (2017) | Yes | Yes | Yes | Yes | Can’t tell | No | Yes | Yes | Yes | Good |
| Engelbrecht (2001) | Yes | Yes | Yes | Yes | Yes | Yes | Can’t tell | Yes | Yes | Very Good |
| Engelbrecht (2003) | Yes | Yes | Yes | Yes | Yes | No | Yes | No | Yes | Good |
| Lopes (2009) | Yes | Yes | Yes | Yes | Yes | Yes | Yes | Yes | Yes | Excellent |
|  |  |  |  |  |  |  |  |  |  | Continued… |
| Majoko (2016) | Yes | Yes | Can’t tell | Yes | Yes | No | Yes | Yes | Can’t tell | Average |
| Majoko (2017) | Yes | Yes | Yes | Yes | Yes | No | Yes | Yes | Yes | Very Good |
| Majoko (2018) | Yes | Yes | Yes | Yes | Yes | No | Yes | Yes | Yes | Very Good |
| Majoko (2019) | Yes | Yes | Yes | Yes | Yes | No | Yes | Yes | Yes | Very Good |
| Mangope (2017) | Yes | Yes | Yes | No | Yes | No | Can’t tell | Yes | Yes | Average |
| Mangope (2018) | Yes | Yes | Yes | Yes | Yes | No | Yes | Yes | Yes | Very Good |
| Mapuranga (2015) | Yes | Yes | Can’t tell | No | Can’t tell | No | No | No | Yes | Very Poor |
| Matsenjwa (2020) | Yes | Yes | Yes | Yes | Yes | No | Yes | No | Yes | Average |
| Mohamed (2012) | Yes | Yes | Can’t tell | No | Yes | No | Yes | Yes | Yes | Average |
| Mokobane (2011) | Yes | Yes | Yes | Yes | Can’t tell | No | Yes | No | Can’t tell | Poor |
| Mukhopadhyay (2019) | Yes | Yes | Can’t tell | Yes | Yes | No | Can’t tell | Yes | Yes | Good |
| Ngcobo (2011) | Yes | Yes | Can’t tell | Can’t tell | Yes | No | Yes | Can’t tell | Yes | Average |
| Okyere (2019a) | Yes | Yes | Yes | Can’t tell | Yes | Yes | Yes | Yes | Yes | Very Good |
| Okyere (2019b) | Yes | Yes | Yes | Yes | Yes | Can’t tell | Yes | Yes | Yes | Very Good |
| Otukile-Mongwaketse (2016) | Yes | Yes | Can’t tell | Yes | Yes | No | Yes | Yes | Yes | Good |
|  |  |  |  |  |  |  |  |  |  | Continued… |
| Potgieter-Groot (2012) | Yes | Yes | Yes | Yes | Yes | Can’t tell | Yes | Yes | Yes | Very Good |
| Seabi (2010) | Yes | Yes | Can’t tell | No | Yes | No | Can’t tell | No | Yes | Poor |
| Uba (2016) | Yes | Yes | Yes | Yes | Yes | No | Can’t tell | Can’t tell | Yes | Good |
| Van Schalkwyk (2017) | Yes | Yes | Can’t tell | Yes | Yes | No | Yes | Yes | Yes | Good |
| Walton (2014) | Yes | Yes | Yes | Yes | Yes | Yes | Yes | Yes | Yes | Excellent |
| Yoro (2020) | Yes | Yes | Can’t tell | Yes | Yes | No | Yes | Yes | Yes | Good |
| Yssel (2007) | Yes | Yes | Yes | Yes | Yes | No | Can’t tell | No | No | Poor |
