# Supplementary material for: Stakeholder experiences, attitudes and perspectives on inclusive education for children with developmental disabilities in sub-Saharan Africa: A systematic review of qualitative studies
Source: Autism. 2022 May 30;26(7):1606–25. doi: 10.1177/13623613221096208 (PMC9483198; doi:10.1177/13623613221096208)
Supplement: sj-docx-5-aut-10.1177_13623613221096208 – Supplemental material for Stakeholder experiences, attitudes and perspectives on inclusive education for children with developmental disabilities in sub-Saharan Africa: A systematic review of qualitative studies [file sj-docx-5-aut-10.1177_13623613221096208.docx]

## Supplementary Material E: Summary of Codebook

| Name of Code | Files | References |
| --- | --- | --- |
| CHARACTERISTICS OF INDIVIDUALS | 0 | 0 |
| Teachers' knowledge and beliefs about inclusion | 1 | 1 |
| good understanding of inclusion | 10 | 21 |
| poor understanding of inclusion | 12 | 24 |
| limited adaptations and consideration of individual needs | 10 | 30 |
| required skills | 0 | 0 |
| curriculum and teaching adaptation | 14 | 23 |
| managing behavioural challenges | 16 | 42 |
| recognition of learners' needs | 11 | 27 |
| teachers' negative attitudes and affects towards inclusion | 13 | 42 |
| teachers' positive attitudes and affects towards inclusion | 10 | 14 |
| Teachers' self-efficacy and state of change | 19 | 107 |
| teachers' (lack of) confidence | 8 | 19 |
| teachers' lack of skills for inclusion | 15 | 38 |
| teachers' strategies | 6 | 6 |
| adaptations and task differentiation | 13 | 29 |
| alternative modes of teaching | 12 | 27 |
| visual aids | 8 | 16 |
| communication strategies | 4 | 9 |
| direct teaching of social and life skills | 5 | 18 |
| leadership roles | 6 | 8 |
| peer to peer | 0 | 0 |
| collaborative learning | 10 | 18 |
| peer to peer mentoring | 6 | 10 |
| reinforcement | 8 | 22 |
| slower pace | 16 | 52 |
| teachers' extra attention | 13 | 30 |
| teachers' strategies to cope with stress | 1 | 1 |
| teachers’ progression towards inclusive education | 14 | 34 |
| Teachers’ relational patterns with pupils | 0 | 0 |
| general comments on relationships | 3 | 3 |
| negative relationships | 6 | 10 |
| nuanced relationships | 4 | 5 |
| positive relationships and empathy | 8 | 18 |
| INNER SETTING | 0 | 0 |
| Compatibility | 0 | 0 |
| class sizes impede meeting special needs | 12 | 21 |
| inclusion as requiring additional time | 12 | 17 |
| inclusion as stressful and-or requiring more effort | 13 | 34 |
| infrastracture and similar challenges | 10 | 29 |
| lack of human resources | 9 | 12 |
| Inner inner setting | 0 | 0 |
| behavioural challenges | 9 | 16 |
| aggression | 10 | 15 |
| disruption | 10 | 23 |
| emotional and temper reactions | 6 | 8 |
| in-class exclusion | 10 | 25 |
| child's disengagement | 12 | 20 |
| peers | 0 | 0 |
| bullying, teasing and exclusion | 13 | 48 |
| friendships and positive relationships | 7 | 19 |
| peer support and models | 12 | 27 |
| promotion of a positive and orderly environment | 16 | 48 |
| Readiness for implementation | 0 | 0 |
| lack of resources | 12 | 31 |
| training | 30 | 130 |
| benefits of helpful training | 8 | 22 |
| poor or limited training provided | 13 | 32 |
| teachers' training needs | 30 | 76 |
| School culture | 0 | 0 |
| corporal punishment | 4 | 9 |
| exclusionary and discriminatory practices in mainstream schools | 14 | 37 |
| gender and age discrimination | 6 | 10 |
| good practice and whole-school approach to inclusion | 8 | 15 |
| low teachers' commitment | 3 | 4 |
| normal vs not normal | 13 | 29 |
| traditional teaching | 3 | 6 |
| Tension for change | 0 | 0 |
| importance of meeting learning needs | 15 | 24 |
| teachers' perceptions of the need for inclusion | 3 | 6 |
| teachers' will to meet special needs | 14 | 21 |
| Within-school networks and collaboration | 0 | 0 |
| lack of peer support | 5 | 7 |
| support of SE teachers and assistants | 5 | 16 |
| teacher-teacher support and sharing | 12 | 24 |
| whole-school collaboration for inclusion | 6 | 7 |
| INTERVENTION CHARACTERISTICS | 0 | 0 |
| Framing of IE | 13 | 21 |
| indigenous frameworks of disability & inclusion | 2 | 10 |
| inclusion as consequence of merit and-or belonging | 2 | 7 |
| IE as a right and mandated from governments | 12 | 22 |
| IE as desirable and beneficial | 0 | 0 |
| alternatives to poor inclusion | 4 | 11 |
| children and parents' preference for IE | 6 | 18 |
| general benefits | 9 | 30 |
| IE promotes integration with peers and the community | 8 | 19 |
| negative effects of poor inclusion | 9 | 25 |
| OUTER SETTING | 0 | 0 |
| External policy & incentives | 0 | 0 |
| in-service training by government (DoE or MoE) | 3 | 6 |
| in-service training by NGOs | 3 | 6 |
| lack of support, incentives and supervision from authorities | 10 | 25 |
| policies, awareness and implementation | 16 | 32 |
| Needs of children with DD | 0 | 0 |
| home and community background | 0 | 0 |
| (lack of) parental support to children | 10 | 27 |
| financial and other home challenges | 17 | 37 |
| financial possibilities and schooling | 6 | 10 |
| symptoms and challenges | 0 | 0 |
| emotional and social challenges | 12 | 27 |
| functional challenges, skill development | 7 | 17 |
| learning and attention challenges in DD | 21 | 65 |
| sensory difficulties and rigidity | 5 | 21 |
| teachers' knowledge | 1 | 2 |
| knowledge of ADHD | 2 | 11 |
| knowledge of ASD or FASD | 5 | 13 |
| knowledge of ID and LD | 3 | 11 |
| limited understanding | 8 | 26 |
| PROCESS | 0 | 0 |
| Engaging stakeholders | 0 | 0 |
| community involvement | 1 | 7 |
| engaging authorities | 9 | 13 |
| importance of stakeholders' collaboration | 17 | 40 |
| networks across schools | 4 | 7 |
| parents | 0 | 0 |
| importance of parents involvement | 20 | 43 |
| negative teacher-parent collaboration | 9 | 24 |
| parents' advocacy | 5 | 12 |
| positive teacher-parent collaboration | 12 | 32 |
| therapists' role | 10 | 24 |
